# Genomic relatedness of colonizing and invasive disease *Klebsiella pneumoniae* isolates in South African infants

**DOI:** 10.1038/s41598-025-92517-4

**Published:** 2025-03-07

**Authors:** Courtney P. Olwagen, Alane Izu, Shama Khan, Lara Van der Merwe, Nicholas J. Dean, Fikile C. Mabena, Stephanie Jones, Gaurav Kwatra, Lubomira Andrew, Urvi Rajyaguru, Robert G. K. Donald, Raphael Simon, Mohamed Said, Firdose L. Nakwa, Jeannette Wadula, Renate Strehlau, Anika M. van Niekerk, Niree Naidoo, Yogandree Ramsamy, Sithembiso C. Velaphi, Ziyaad Dangor, Shabir A. Madhi

**Affiliations:** 1 South Africa Medical Research Council Vaccines and Infectious Diseases Analytics Research Unit, Faculty of Health Science, University of the Witwatersrand, Johannesburg, South Africa; 2 Division of Infectious Diseases, Department of Pediatrics, Cincinnati Children’s Hospital Medical Center, University of Cincinnati, Cincinnati, OH USA; 3 Department of Clinical Microbiology, Christian Medical College, Vellore, India; 4 Vaccine Research and Development, Pfizer, Pearl River, NY USA; 5 Department of Medical Microbiology, University of Pretoria, Pretoria, South Africa; 6 National Health Laboratory Services, Tshwane Academic Division, Tshwane, South Africa; 7 Department of Paediatrics and Child Health, Chris Hani Baragwanath Academic Hospital, School of Clinical Medicine, Faculty of Health Sciences, University of the Witwatersrand, Johannesburg, South Africa; 8 Department of Clinical Microbiology & Infectious Diseases, National Health Laboratory Services, Faculty of Health Science, University of Witwatersrand, Johannesburg, South Africa; 9 Wits VIDA Nkanyezi research unit, Department of Paediatrics and Child Health, School of Clinical Medicine, University of the Witwatersrand, Johannesburg, South Africa; 10 Mowbray Maternity Hospital. Division of Neonatal Medicine, Department of Paediatrics, Faculty of Health Sciences, University of Cape Town, Cape Town, South Africa; 11 Antimicrobial Research Unit, University of KwaZulu-Natal, South Africa, Durban, South Africa; 12 Department of Medical Microbiology, National Health Laboratory Service, Prince Mshiyeni Hospital, Umlazi, KwaZulu-Natal South Africa; 13 Wits Infectious Diseases and Oncology Research Institute, Faculty of Health Science, University of the Witwatersrand, Johannesburg, South Africa

**Keywords:** Invasive disease potential, *Klebsiella pneumonaie*, Colonization, Invasive disease, Africa, Bacteria, Neonatal sepsis, Whole genome amplification

## Abstract

*Klebsiella pneumoniae* (KPn) colonizes multiple anatomical sites and is a leading cause of invasive disease and death in African children; however, there is no comparative genomic analysis between colonizing and invasive strains. This study investigated the genomic relatedness of KPn colonizing and invasive isolates in South African infants; and evaluated the relative invasiveness of KPn isolates based on sequence types (ST), capsular (KL), and lipopolysaccharide (O) loci by calculating case-carrier ratios (CCRs). There was less genomic diversity amongst invasive (22 ST, 17 K-loci) than colonizing isolates (31 ST, 29 K-loci), with invasive isolates being 8.59-fold and 3.49-fold more likely to harbour genes encoding for multi-drug resistance and yersiniabactin production compared with colonizing isolates. The CCRs for KL102 and O1/O2v2 were > 1, and < 1 for KL8, ST1414, and O1O2v1. Identifying high-risk strains, including KL102 and O1O2v2, that may have a higher potential to cause invasive disease, could enhance risk assessment and management strategies in vulnerable populations.

**Supplementary Information:**

The online version contains supplementary material available at 10.1038/s41598-025-92517-4.

## Introduction

Sub-Saharan Africa reports the highest rates of neonatal morbidity and mortality worldwide, with *Klebsiella pneumoniae* (KPn) being a leading infective cause^1–3^. Recent findings from the Global Burden of Disease Study reported that in 2019, The disability-adjusted Life Years (DALYs) associated with KPn in children under 5 years of age in Sub-Saharan Africa were 5, 637.3 (4, 254.7–7, 317.9) per 100, 000 population^4^.

*KPn* colonizes multiple anatomical sites including the skin, respiratory, and gastrointestinal mucosa^5,6^. Although the prevalence of KPn colonization varies geographically^7^, data on colonization in African infants is limited, primarily due to challenges in surveillance and diagnostic capabilities, highlighting the urgent need for enhanced epidemiological surveillance programs in this setting. Nevertheless, we recently reported that 59% of hospitalized neonates became colonized by KPn in at least one of the three anatomical sites investigated (skin, rectal, and nasopharynx) during their hospital stay in a microbial surveillance study undertaken in a regional South African hospital^8^. Similarly, findings from Kenya (55%), Ghana (42%), and Ethiopia (68%) also report a high prevalence of KPn colonization in neonates hospitalized for more than 2–3 days^9–11^, most likely due to the high exposure to KPn in the neonatal units of these hospitals.

Colonization with KPn has been reported as a risk factor for invasive disease, particularly in vulnerable populations including neonates, the elderly, and those with weakened immune systems^5,12^. Nevertheless, only a small subset (4–6%) of colonized individuals progress to developing invasive KPn disease^8,12,13^. The dynamics of colonization with KPn and the link between carriage and subsequent infections are yet to be determined. Further, there is a paucity of comparative genomic analysis of KPn isolates from colonizing compared with invasive disease isolates, with no data available from African infants.

The objectives of our study were to investigate the genomic relatedness of KPn isolates associated with invasive disease compared with colonizing isolates in hospitalized South African infants up to 90 days of age; and to evaluate for the relative invasiveness of KPn isolates based on the sequence types (ST), capsular (KL) and lipopolysaccharide (O) loci.

## Methods

### Study population

The study characterized the genomes of colonizing KPn isolates (carriers) identified through longitudinal microbial surveillance of infants hospitalized post-delivery at the Thelle Mogoerane Regional Hospital (TMRH) in Johannesburg, South Africa between October 25th, 2020, and April 30th, 2021 as previously described^8^. Briefly, rectal, nasal, and skin swabs were collected from 102 neonates within 24 h of hospital admission and then every 48–96 h until discharge or death. All swab samples were cultured for multiple organisms including KPn, and all isolates were stored at – 80 °C for downstream whole genome sequencing (WGS). Sequencing was preformed on all isolates when KPn was identified across multiple anatomical sites in the same infant.

Of the infants colonized with KPn, 5% (*n* = 3/59) developed invasive bacterial disease. Sequencing was not undertaken for one child who developed invasive disease; however, genomic analysis revealed that the colonizing (child 1:ST2:KL9:O1/O2v2; child 2: ST17:KL25:05) and invasive (child 1:ST17:KL62:O1O2v2; child 2:ST307:KL102:O1/O2v2) Kpn isolates differed for each of the two children who developed invasive disease and sequencing was undertaken^8^. These two invasive disease isolates collected from TMRH were not included in the invasive cohort described below.

The genomes of invasive disease isolates (cases) identified between March 4th, 2019, and February 27th, 2021 in an observational study on invasive bacterial disease, as described^14^, were sequenced. Briefly, KPn isolates from blood or CSF were collected from infants < 90 days of age through surveillance undertaken at four centres in Gauteng Province (Chris Hani Baragwanath Academic Hospital (CHBAH), Rahima Moosa Mother and Child Hospital (RMMCH), Charlotte Maxeke Johannesburg Academic Hospital (CMJAH), Tshwane Academic Laboratory Network (TALM)], one in Kwazulu-Natal province [Prince Mshiyeni Memorial Hospital (PMMH)], and one in the Western Cape province [Mowbray Maternity Hospital (MMH)], in South Africa. In some instances, repeat blood and CSF cultures were undertaken during hospitalization at the attending physician’s discretion to evaluate for response to antibiotic therapy or to investigate newly suspected sepsis events. Cultures collected more than 7 days apart were considered to be a new septic event and underwent sequencing.

### Whole genome sequencing of *K. pneumoniae* isolates

Archived colonizing and invasive KPn isolates were sub-cultured and genomic DNA was extracted from a single colony, that had undergone mass growth, using standard protocols. Following extraction, NexteraXT libraries were prepared (Illumina, San Diego, CA) and sequenced on the MiSeq platform, producing 300-base paired-end reads (Illumina, San Diego, CA). After sequencing, FASTQC version v0.12.1 was used to determine the quality of the raw reads and the paired-end (PE) reads were processed with the Jekesa pipeline with “-s option” to identify the MLST schema “kpneumoniae” for KPn WGS typing. Trim Galore was used to remove adaptors, ambiguous reads, and low-quality bases (Q > 30 and length > 50 bp), and de novo assemblies were generated using SPAdes and Shovill packages. The assembly quality was then assessed with QUAST 5 and assembly metrics were computed. Processed sequences were then cross-referenced with the Pathogenwatch6 database to confirm species identification. Sequence contamination was verified using Confinder.

For WGS-based sequence typing, PE reads were used for in silico prediction of multi-locus sequence types (MLST) based on the *Klebsiella* PasteurMLST scheme. Additionally, BIGSdb-Pasteur was used to assign core-genome MLST (cgMLST) to the KPn isolates. The Kaptive tool was used for Capsule K and O-Serotype predictions. To profile antimicrobial resistance, antimicrobial resistance genes (ARGs) were identified in the PE reads using Kleborate against the curated Comprehensive Antibiotic Resistance Database (CARD), including ResFinder 9. Plasmid types were identified with PlasmidFinder v2.1, while Virulence genes were detected using Kleborate, which searches for key virulence loci (ybt, iuc, iro, clb, and rmpADC) via BLASTn search. Lastly, the whole genome phylogenetic relationship of KPn isolates with a reference strain was assessed and included identifying single nucleotide polymorphisms (SNPs) through De novo assemblies aligned against the reference strain. The phylogeny of all KPn isolates was determined through wgMLST and cgMLST. The sARGs and plasmid types were investigated through ResFinder and PlasmidFinder databases respectively. iTol12 was used to generate and visualize a tailored phylogenetic tree.

### Data availability

The raw PE reads are available through GenBank under BioProject PRJNA1175467 (https://dataview.ncbi.nlm.nih.gov/object/PRJNA1175467?reviewer=95hpon1d4f0n56qvljcfqq6mi9). Associated metadata will be made available to researchers who provide a methodologically sound proposal. Data will only be made available if approval is granted from the Human Research Ethics Committee, University of Witwatersrand, Johannesburg, South Africa. Further, all requesters will need to sign a data transfer agreement. Requests should be directed to the corresponding author.

### Statistical analysis

Colonizing and invasive disease isolates collected more than 7 days apart were sequenced and included in the final analysis only if the genomic analysis revealed a different KPn strain from the first isolate sequenced. Colonizing isolates collected from multiple anatomical sites in the same infant were only included if genomic analysis revealed that different strains colonized different sites. Multi-drug resistance (MDR) was defined as resistance to ≥ 3 acquired classes of antimicrobials^15^. As a proxy for the relative invasiveness of KPn isolates, the invasiveness was calculated by measuring the case carrier ratio of the odds (CCR) for each ST, K-locus, O-locus, and O-antigen type by dividing the number of isolates with the genetic characteristic of interest by the number of isolates without the characteristic of interest^16^. The CCRs were only calculated for isolates represented by KPn-specific ST, K-locus, O-Locus, or O-antigen disease, and where the same was present in colonizing isolates. A logistic regression model was used to compare the presence of antimicrobial resistance markers (ARM) and virulence factors between invasive and colonizing isolates. The prevalence of each ST, K-locus, O-locus, and O-antigen is reported along with odds ratios (OR) and 95% confidence intervals (CI). Where too few observations were identified in either invasive or colonizing isolates, the significance between the groups was determined using Fisher’s exact test. P-values of < 0.05 were considered statistically significant and no adjustment was undertaken for multiplicity in this hypothesis-generating study.

Data was analyzed with STATA Version 11.0 (StataCorp, Texas, USA) and R Version 4.1.1 (Vienna, Austria).

### Role of the funding source

The study was co-funded by The Bill & Melinda Gates Foundation (BMGF: INV005773 and INV-062172) and Pfizer who sponsored the sequencing of a subset of isolates which was undertaken by co-authors (L.A.; U.R.; R.G.K.D.; R.S.) employed by Pfizer. The Pfizer co-authors were involved in the data collection and analysis of the study. The BMGF played no role in the study design, analysis, or interpretations of the results. The corresponding author was responsible for the final decision to submit the publication and has full access to the study data.

### Ethics approval

The Human Medical Human Research Ethics Committee of the University of Witwatersrand granted ethics consent for all studies and relevant approvals from hospital management were obtained before study initiation. Written, informed consent was obtained from the mother/guardian of all the study participants at the time of initial enrolment. All studies were conducted in compliance with Harmonisation Good Clinical Practice guidelines and the ethical principles of the Declaration of Helsinki methods.

## Results

**Fig. 1:**
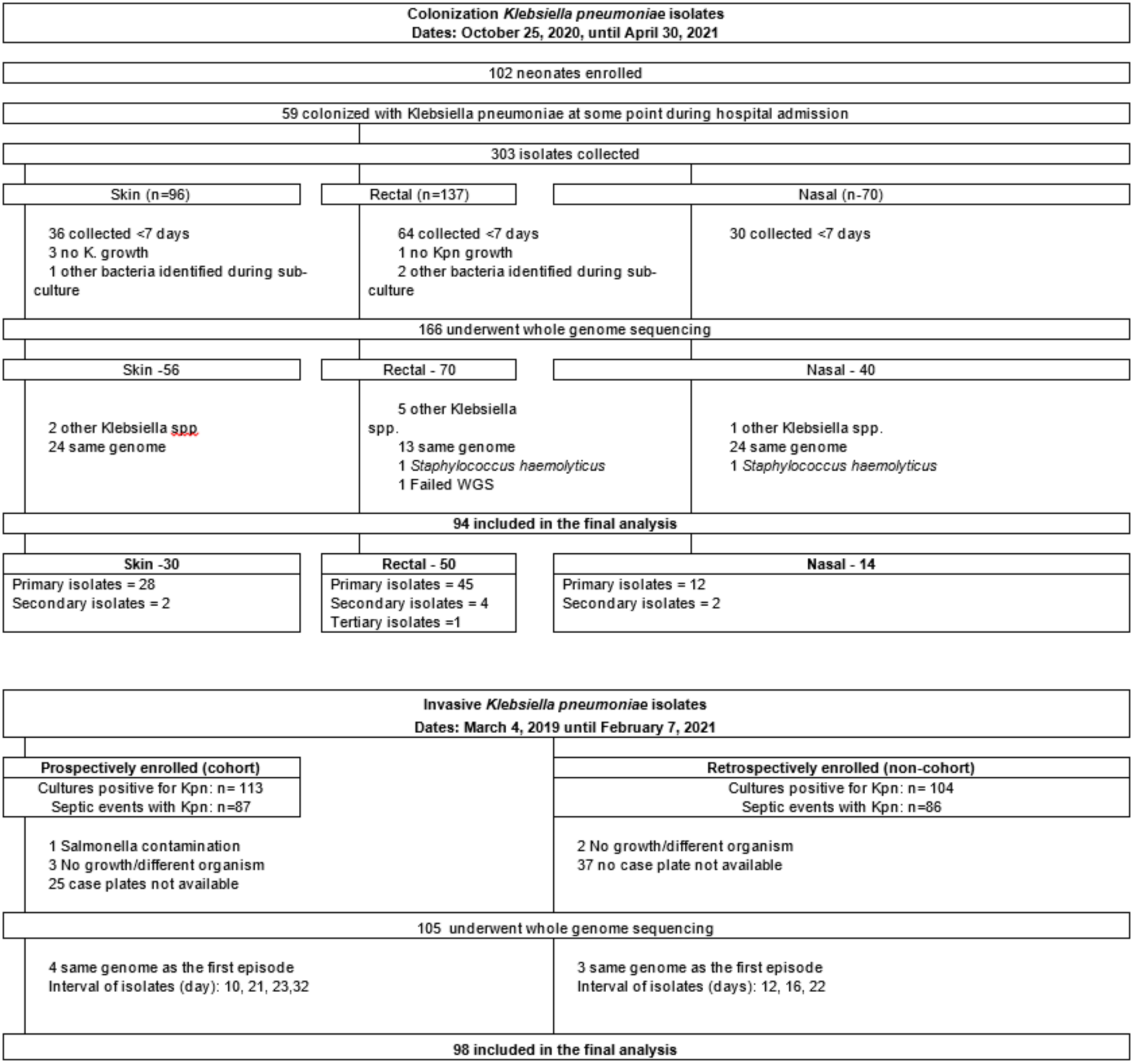
Study profile. KPn, *Klebsiella pneumonia. Klebsiella pneumoniae* isolates collected more than 7 days apart were considered a separate septic event and were sequenced. Isolates were only included in the final analysis if genomic analysis revealed a different KPn strain from the first isolate sequenced.

Overall, 303 KPn colonizing isolates were collected from the 59 infants who were colonized with KPn, in the longitudinal surveillance study, following hospitalization after delivery at the TMRH. Whole genome sequencing was undertaken in 166 (55% of 303) of the colonizing isolates. Reasons for sequencing not being undertaken included 130 isolates collected within 7 days of an earlier KPn isolate and therefore more likely to be an identical strain as the earlier isolate, and KPn not identified during sub-culture for seven isolates. Following genotypic analysis, one isolate failed WGS, eight isolates were determined to be another Klebsiella species, and two isolates were determined to be *Staphylococcus haemolyticus*—all of which were excluded from the final analysis. The final analysis was limited to one KPn strain per participant, and thus an additional 25 isolates collected from 14 infants were excluded from the final analysis as their genotypes were identical to an earlier isolate sequenced that was collected > 7 days apart from the same infant. Also, 36 isolates collected from a different anatomical site from the same infant (*n* = 30) at the same sampling time point, that were genetically identical, were excluded, with the included isolates being chosen based on a hierarchical approach of first including rectal, nasal, and lastly skin swab samples (Fig. 1).

A total of 173 culture-confirmed invasive KPn cases were enrolled during surveillance of invasive bacterial disease. Sequencing was undertaken in 105 (61%) of the invasive isolates. Reasons for sequencing not being undertaken include 62 isolates not being retrieved from the laboratories and KPn not identified during sub-culture for six isolates. Following genotypic analysis, an additional seven isolates were determined to be due to the same septic event, based on no genotypic differences from an earlier invasive isolate sequenced from the same infant despite being collected more than seven days apart, were also excluded from further analysis (Fig. 1). The interval between collection for the six isolates from the same participant were 10, 12, 16, 21, 22, 23, and 32 days, respectively. One participant had isolates included from two distinct septic events, which were collected 45 days apart.

One hundred and ninety-two isolates (94 colonizing, 98 invasive) were included in the final analysis. 56% (107/192) of all KPn isolates were from males and 31% (60/192) were in infants born to women living with HIV (Table 1). Overall, 98% (96/98) of the invasive isolates were from blood and 2% (2/98) from CSF culture. Fifty-three percent (50/94) of the colonizing isolates were from rectal swab samples, 32% (30/94) from nasal swabs, and 15% (14/94) from skin swab samples. A higher proportion of infants who developed invasive KPn disease were born prematurely at < 34 weeks gestation (76%, 71/98 vs. 44%, 41/94; *p* < 0.0001) and consequently had lower birthweight (1238 g, IQR 998–1812 vs. 1950 g, IQR 1380–2898; *p* < 0.0001) compared with infants colonized but not infected with KPn (Table 1). Other differences included cases being hospitalized for a longer duration (43 days, IQR 15–81) compared with the carriers (13 days, IQR 7–31; *p* < 0.0001). Additionally, the case fatality risk was 42% (35/98) in the invasive disease cases compared with 7% (7/94; *p* < 0.0001) in the colonization cohort (which excluded the three invasive disease cases).

**Table 1:** Clinical characteristics of infants up to 90 days of age with *K. pneumoniae* isolates that were sequenced.

	Overall (*n* = 192)	Invasive (*n* = 98)	Colonization (*n* = 94)	*p*-value
Gender n (%)				
Female	84 (44%)	42 (43.3%)	42 (44.7%)	
Male	107 (56%)	55 (56.7%)	52 (55.3%)	0.885
Birth weight, n (%)				
< 1500 g	90 (47.6%)	62 (65.3%)	28 (29.8%)	
1500–< 2500 g	53 (28%)	19 (20%)	34 (36.2%)	
2500–<3500 g	35 (18.5%)	9 (9.5%)	26 (27.7%)	
≥ 3500 g	11 (5.8%)	5 (5.3%)	6 (6.4%)	< 0.001
Median birthweight (IQR)	1570 (1100–2430). *n* = 189	1238 (997.5–1812.5). *n* = 95	1950 (1380–2897.5). *n* = 94	< 0.001
Gestational age, n (%)				
< 34 weeks	112 (59.6%)	71 (75.5%)	41 (43.6%)	
34–< 37 weeks	26 (13.8%)	9 (9.6%)	17 (18.1%)	
≥ 37 weeks	50 (26.6%)	14 (14.9%)	36 (38.3%)	< 0.001
Median gestational age (IQR)	32 (29–37). *n* = 188	30 (28–33). *n* = 94	34 (31–37). *n* = 94	< 0.001
Mode of delivery, n (%)				
Caesarean section	105 (54.7%)	61 (62.2%)	44 (46.8%)	
Vaginal cephalic	87 (45.3%)	37 (37.8%)	50 (53.2%)	0.042
Mothers HIV status, n (%)				
HIV−	132 (68.8%)	67 (68.4%)	65 (69.1%)	
HIV+	60 (31.2%)	31 (31.6%)	29 (30.9%)	> 0.999
Median length of hospital stay in days (IQR)	24 (9–53). *n* = 176	42.5 (14.75–80.5). *n* = 84	13 (6.75–31). *n* = 92	< 0.001
Infant outcome, n (%)				
Demised	42 (23.6%)	35 (41.7%)	7 (7.4%)	
Discharged home	135 (75.8%)	49 (58.3%)	86 (91.5%)	
Transferred	1 (0.6%)	0 (0%)	1 (1.1%)	< 0.001

Participant demographic characteristics were summarized using frequency distributions for categorical variables and mean with standard deviations for continuous variables. The chi-square test or student T-test was used to compare cases and carriers as appropriate.

P-values were calculated by comparing invasive and colonizing isolates and p of < 0.05 were considered statistically significant.

### Phylogenetic analysis

**Fig. 2:**
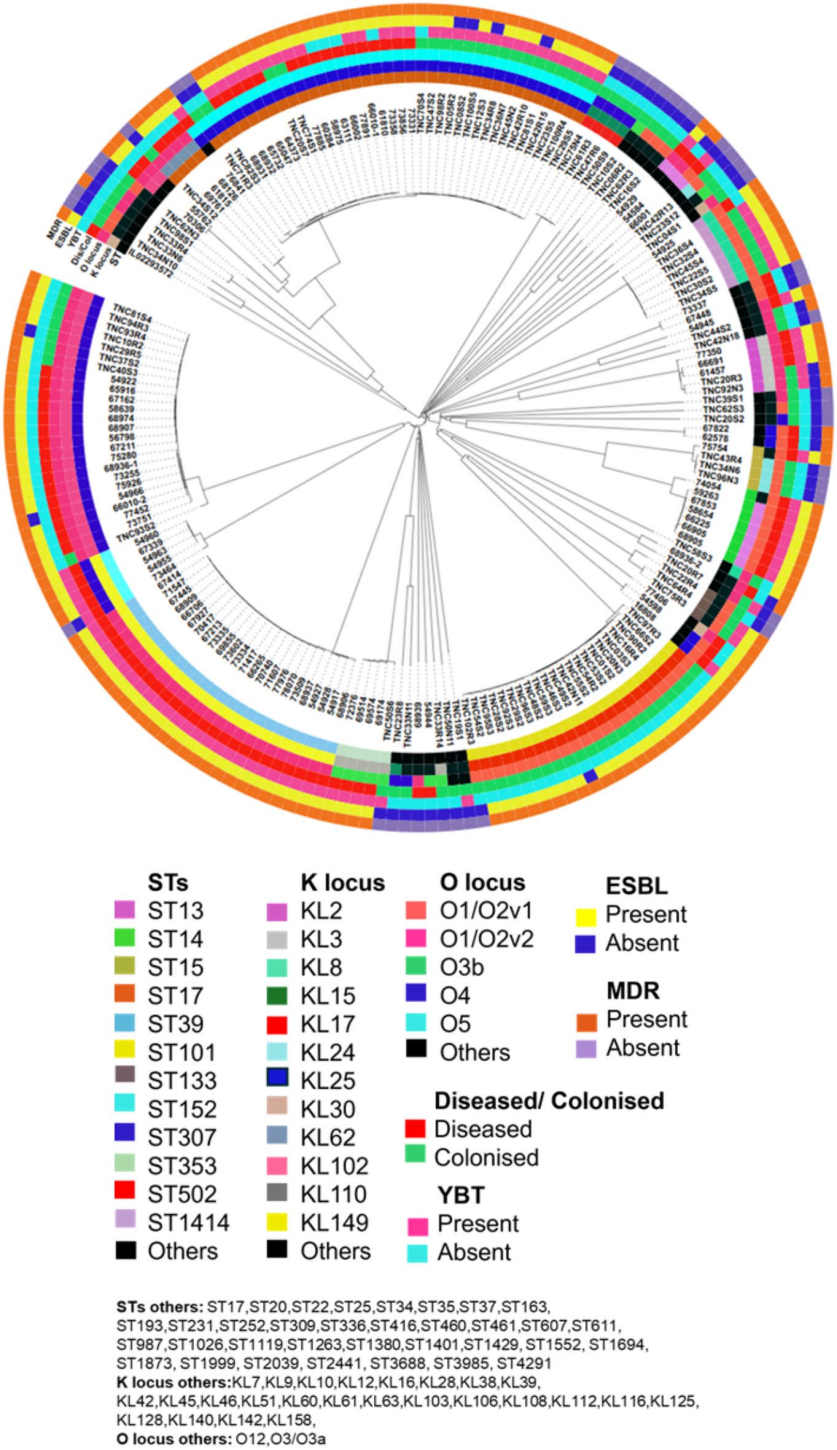
Phylogenetic tree of *K. pneumoniae* strains amongst invasive and colonization isolates in South African infants up to 90 days of age. The circle starting from inside indicates (i) ST (ii) K-loci (iii) O-loci (iv) carrier or case (v) yersiniabactin, (vi) ESBL, and (vii) multi-drug resistance (MDR). The clustering indicates that strains within a clade are probably part of a distinct lineage or have recently diverged due to shared selective pressures including host colonization or adapting antimicrobial exposure. This pattern may highlight the potential for colonizing strains to acquire additional factors that enable pathogenesis, such as resistance or virulence genes. ST17 was found in both invasive and colonizing isolates, suggesting it is a lineage capable of transitioning between asymptomatic carriage and invasive infection. This could reflect the presence of key virulence genes or host adaptation mechanisms. ST101 is often associated with multidrug resistance and has been implicated in nosocomial outbreaks, making its dual role significant for surveillance and treatment strategies. Other common prevalent STs include ST39 and ST307, reflecting their considerable role in this pathogen’s regional epidemiology. These STs are often linked to AMR contributing to their spread in hospital settings. The clustering of these prevalent STs across both invasive and colonizing isolates reflects the adaption capacity of these lineages to distinct niches. This tree also indicates the isolates with MDR or ESBL genes may form clusters with shared characteristics, indicating transmission of resistant strains or genetic elements. The absence of MDR/ESBLs in certain isolates suggests they may belong to more susceptible or non-pathogenic clades. Clusters with similar MDR/ESBL profiles might correspond to outbreaks or clonal expansions, driven by environmental pressures such as antibiotic use in healthcare or agriculture. The ring in the tree indicates the presence and absence of the yersiniabactin gene cluster (ybt) in each isolate. The ybt is a siderophore system used by Kpn for iron acquisition, contributing to virulence. Clades or clusters with ybt presence indicate lineages with higher pathogenic potential as ybt enhances survival and proliferation in the host. The absence of ybt suggests that isolates lacking the yersiniabactin gene cluster may have a reduced capacity for iron acquisition potentially limiting their virulence.

Phylogenetic analysis (Fig. 2) grouped the KPn strains into multiple clades confirming the high genetic diversity. The closely grouped isolates on the tree are more genetically similar and clusters between the colonized and invasive isolates are indicative of a closer evolutionary history or a recent common ancestor. Such as in the case of ST17 and ST101 which were common in invasive and colonizing isolates with shared lineages.

### Sequence types

**Fig. 3:**
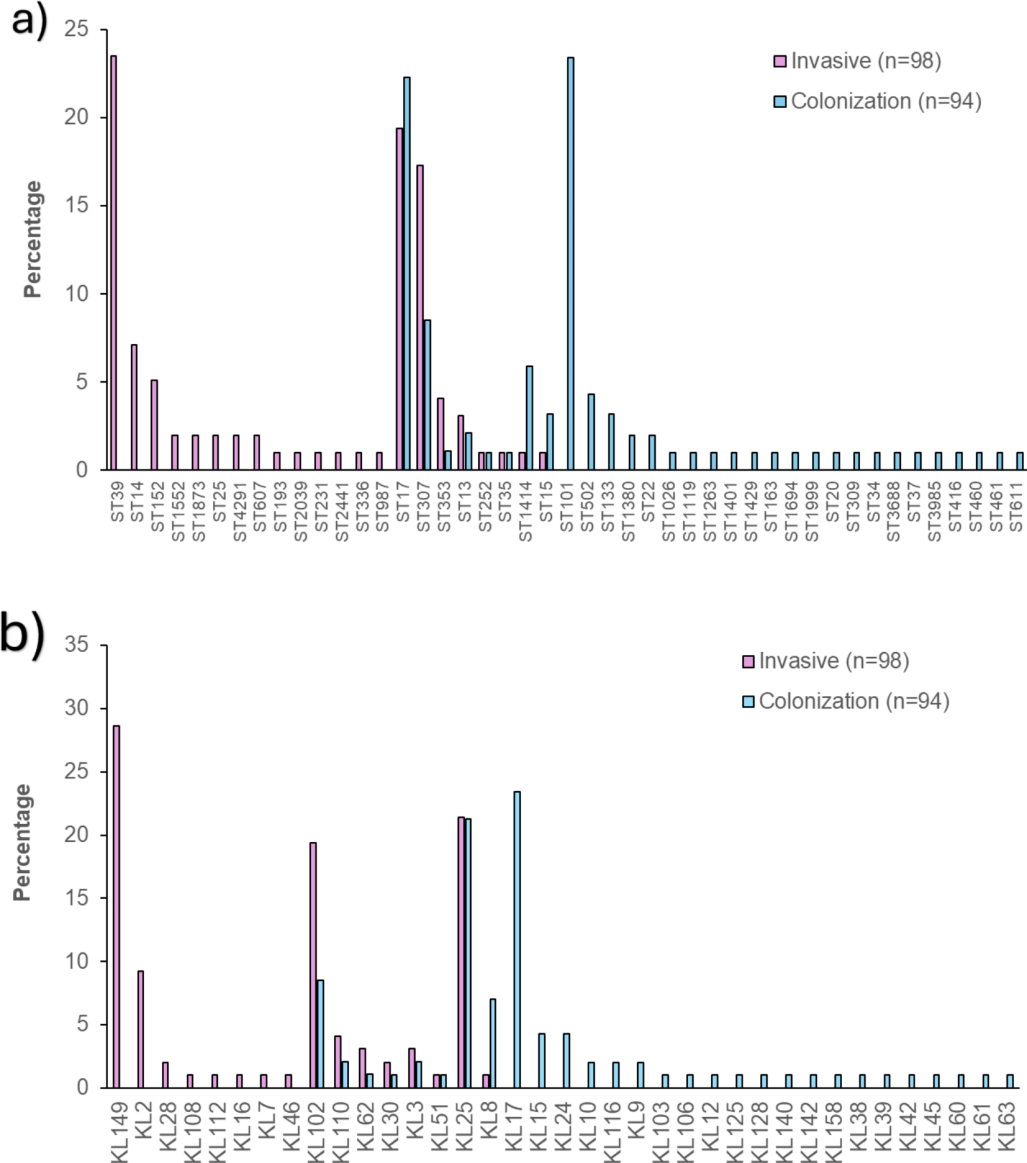

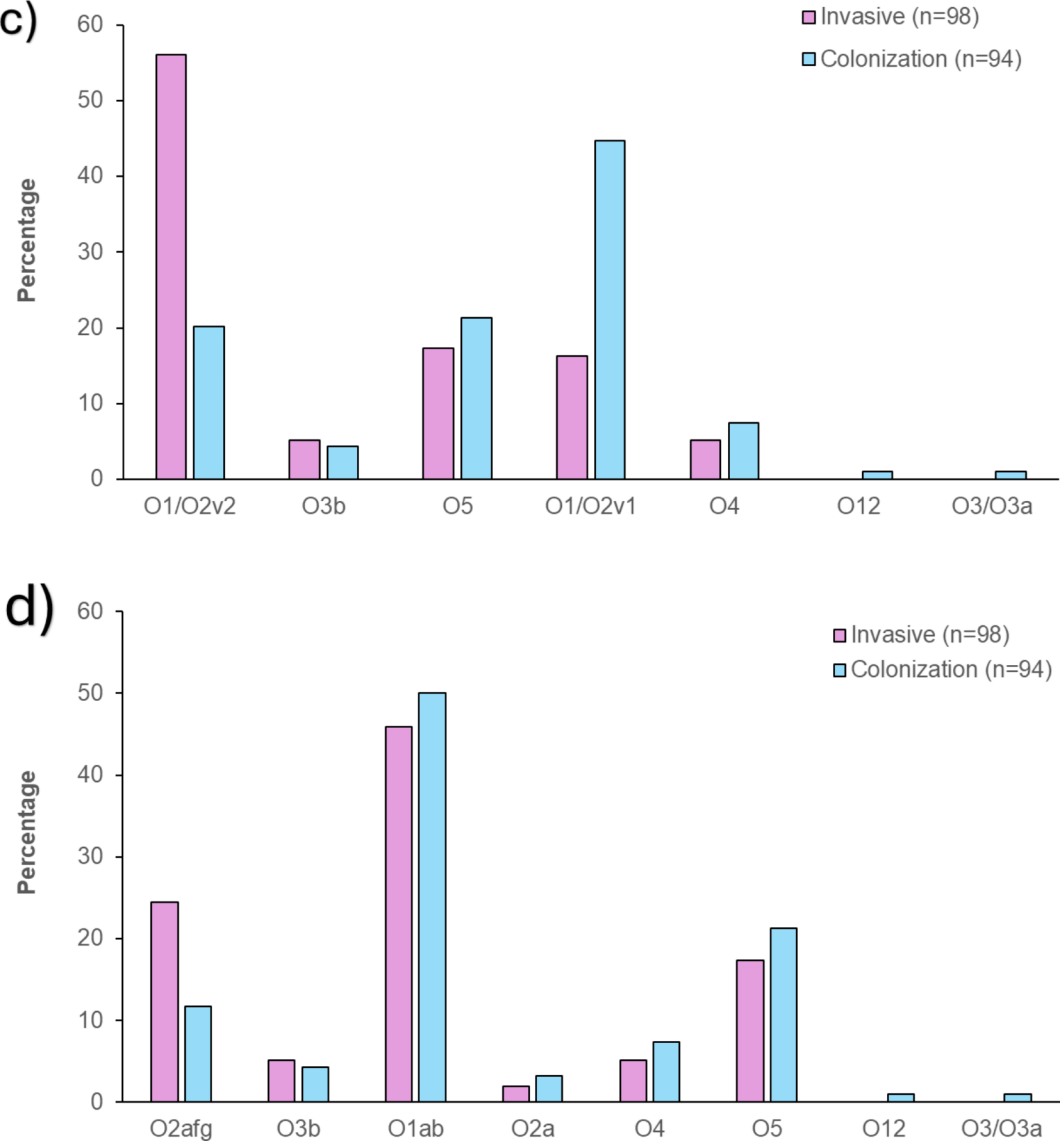
Sequence type (**a**), K-loci (**b**), O-loci (**c**), and O antigen type (**d**) distribution of *Klebsiella pneumoniae* amongst invasive and colonization isolates in South African infants up to 90 days of age.

**Fig. 4:**
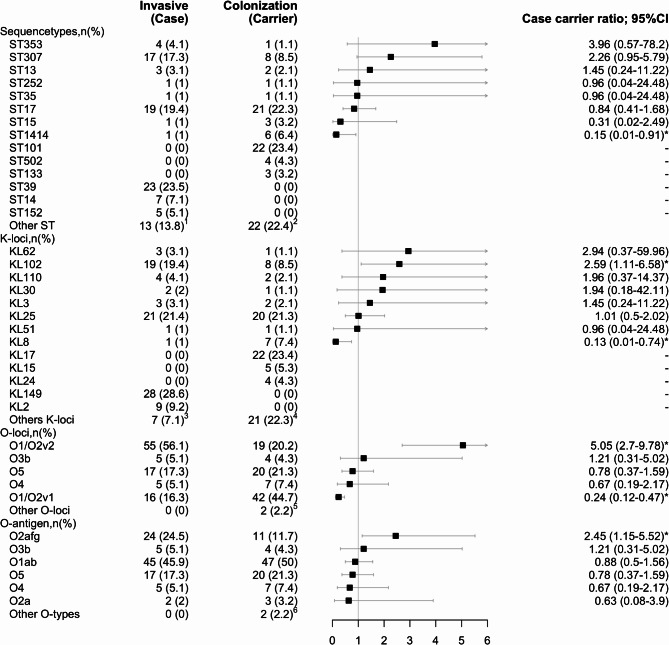
Forrest plot illustrating case carrier ratio of *Klebsiella pneumonia.* Too few instances to calculate the ratio and 95% Confidence intervals (CI). ^1^Sequence types contributing < 3 invasive disease isolates: ST1552 (*n* = 2), ST1873 (*n* = 2), ST25 (*n* = 1), ST4291 (*n* = 1), ST607 (*n* = 1), ST193 (*n* = 1), ST2039 (*n* = 1), ST231 (*n* = 1), ST2441 (*n* = 1), ST336 (*n* = 1), and ST987 (*n* = 1). ^2^Sequence types contributing < 3 colonizing isolates: ST1380 (*n* = 2), ST22 (*n* = 2), ST416 (*n* = 1), ST1026 (*n* = 1), ST1119 (*n* = 1), ST1263 (*n* = 1), ST1401 (*n* = 1), ST1429 (*n* = 1), ST63 (*n* = 1), ST1694 (*n* = 1), ST1999 (*n* = 1), ST20 (*n* = 1), ST309 (*n* = 1), ST34 (*n* = 1), ST3688 (*n* = 1), ST37 (*n* = 1), ST3985 (*n* = 1), ST37 (*n* = 1), ST3985 (*n* = 1), ST460 (*n* = 1), ST461 (*n* = 1), and ST611 (*n* = 1). ^3^K-loci contributing < 3 invasive disease isolates: KL28 (*n* = 2), KL108 (*n* = 1), KL112 (*n* = 1), KL16 (*n* = 1), KL46 (*n* = 1), and KL7 (*n* = 1). ^4^K-loci contributing < 3 colonizing isolates: KL10 (*n* = 2), KL116 (*n* = 2), KL9 (*n* = 2), KL103 (*n* = 1), KL106 (*n* = 1), KL12 (*n* = 1), KL125 (*n* = 1), KL128 (*n* = 1), KL140 (*n* = 1), KL142 (*n* = 1), KL158 (*n* = 1), KL38 (*n* = 1), KL39 (*n* = 1), KL42 (*n* = 1), KL45 (*n* = 1), KL60 (*n* = 1), KL61 (*n* = 1), and KL63 (*n* = 1). ^5^O-loci contributing < 3 isolates to the total 192 isolates: O12 (*n* = 1) and O3/O3a (*n* = 1). ^6^O-types contributing < 3 isolates to the total 192 isolates: O12 (*n* = 1) and O3/O3a (*n* = 1). *P < 0.005.

The distribution of the sequence types (ST) differed between invasive and colonizing isolates, with 22 and 31 STs identified, respectively. Eight STs were detected in both invasive and colonizing isolates, including ST17 (19%, 19/98 vs. 22%, 21/94), ST307 (17%, 17/98 vs. 9%, 8/94), ST353 (4%, 4/98 vs. 1%, 1/94), ST13 (3%,3/98 vs. 2%;2/94), ST252 (1%, 1/98 vs. 1%, 1/94), ST35 (1%, 1/98 vs. 1%, 1/94), ST1414 (1%, 1/98 vs. 6%, 6/94), and ST15 (1%, 1/98 vs. 3%, 3/94 (Fig. 3a). The CCR of ST307 (2.26; 95% CI 0.95–5.79) and ST353 (3.96; 95% CI 0.57–78.2) was > 1, while the CCR for ST1414 (0.15; 95% CI 0.01–0.91), ST15 (0.31; 95% CI 0.02–2.49), and ST17 (0.84; 95% CI 0.41–1.68) were < 1, albeit only significant for ST1414 (Fig. 4). The distribution of STs and associated CCR stratified by location centre is detailed in Supplementary Tables 1 and Supplementary Fig. 1.

Sequence types ST39, ST14, and ST152 were exclusively identified in 24% (23/98), 7% (7/98), and 5% (5/98) of the invasive isolates, with most of ST39 (87%, 20/23) and ST14 (71%, 5/7) cases isolated from the CHBAH and most of the ST152 cases isolated from TALM (75%, 3/5); Supplementary Fig. 1. Additionally, ST1552, ST1873, ST25, ST4291, ST607, ST193, ST2039, ST231, ST2441, ST336, and ST987 were also exclusively identified in the invasive isolates, albeit at a lower prevalence (1–2%) (Fig. 3a). Conversely, ST101, ST502, and ST133 were identified exclusively in 23% (22/94), 4% (4/94), and 3% (3/94), respectively, of the colonizing isolates. Additionally, ST1380, ST22, ST416, ST1026, ST1119, ST1263, ST1401, ST1429, ST63, ST1694, ST1999, ST20, ST309, ST34, ST3688, ST37, ST3985, ST37, ST3985, ST460, ST461, ST611 were exclusively identified in < 2% of the colonizing isolates (Fig. 3a).

### K locus

The K-loci distribution differed between the invasive and colonizing isolates, with 17 and 29 different K-loci identified, respectively. Eight K-loci, namely KL102 (19%, 19/98 vs. 9%, 8/94), KL110 (4%, 4/98 vs. 2%, 2/94), KL62 (3%, 3/98 vs. 1%, 1/94), KL3 (3%, 3/98 vs. 2%, 2/94), KL25 (21%, 21/98 vs. 21%, 20/94), KL30 (2%, 2/98 vs. 1%, 1/94), KL51 (1%, 1/98 vs. 1%, 1/94), and KL8 (1%, 1/98 vs. 7%, 7/94) were identified in invasive and colonizing isolates, respectively (Fig. 3b). The CCR of KL30 (1.94; 95% CI 0.18–42.11), KL110 (1.96; 95% CI 0.37–14.37), KL62 (2.94; 95% CI 0.37–59.96), and KL102 (2.59; 95% CI 1.11–6.58) was > 1, while the CCR was < 1 for KL8 (0.13; 95% CI 0.01–0.74). The CCRs were only statistically significant for KL8 and KL102 (Fig. 4). The distribution of k-loci and associated CCRs stratified by location centre is detailed in Supplementary Tables 1 and Supplementary Fig. 2.

KL149 and KL2 were identified exclusively in 29% (28/98) and 9% (9/98) of invasive isolates (Fig. 3b), respectively with most of the KL149 (79%, 22/28) and KL2 (56%, 5/9) cases identified in invasive isolates collected from the CHBAH (Supplementary Fig. 2). Additionally, KL28, KL108, KL112, KL16, KL46, and KL7 were also identified exclusively in the invasive isolates, albeit at a low frequency (1–2%) (Fig. 3b). Conversely, KL17, KL15, and KL24 were identified exclusively in 23% (22/94), 5% (5/94), and 4% (4/94) of the colonizing isolates, respectively. Additionally, KL10, KL116, KL9, KL103, KL106, KL12, KL125, KL128, KL140, KL142, KL158, KL38, KL39, KL42, KL45, KL60, KL61, and KL63 were each exclusively identified in colonization isolates at < 2% frequency (Fig. 3b).

### O locus

The prevalence of O1/O2v2 (56%, 55/98 vs. 20%, 19/94) was significantly higher in invasive compared with colonizing isolates (Fig. 3c), with a CCR of 5.05 (95% CI 2.70–9.78; Fig. 4). In contrast, the prevalence of O1/O2v1 (16%, 16/98 vs. 45%, 42/94) was significantly lower in the invasive than colonizing isolates (Fig. 3c) with a CCR of 0.24 (095% CI 0.12–0.47; Fig. 4). The distribution of O-loci and associated CCR stratified by location centre is detailed in Supplementary Tables 1 and Supplementary Fig. 3.

### O-antigen types

The prevalence of O2afg was higher in invasive (24%, 24/98) compared with colonizing (12%, 11/94) isolates (Fig. 3d), with a CCR of 2.45 (95% CI 1.15–5.52; Fig. 4). There was a similar prevalence of O5 (17%, 17/98 vs. 21%, 20/94), O1 (45%, 45/98 vs. 50%, 47/94), and O4 (5%, 5/98 vs. 7%, 7/94) in invasive compared with colonizing isolates (CCR < 1). The distribution of O-antigen and associated CCR stratified by hospital location is detailed in Supplementary Tables 1 and Supplementary Fig. 4.

### Virulence factors

**Fig. 5:**
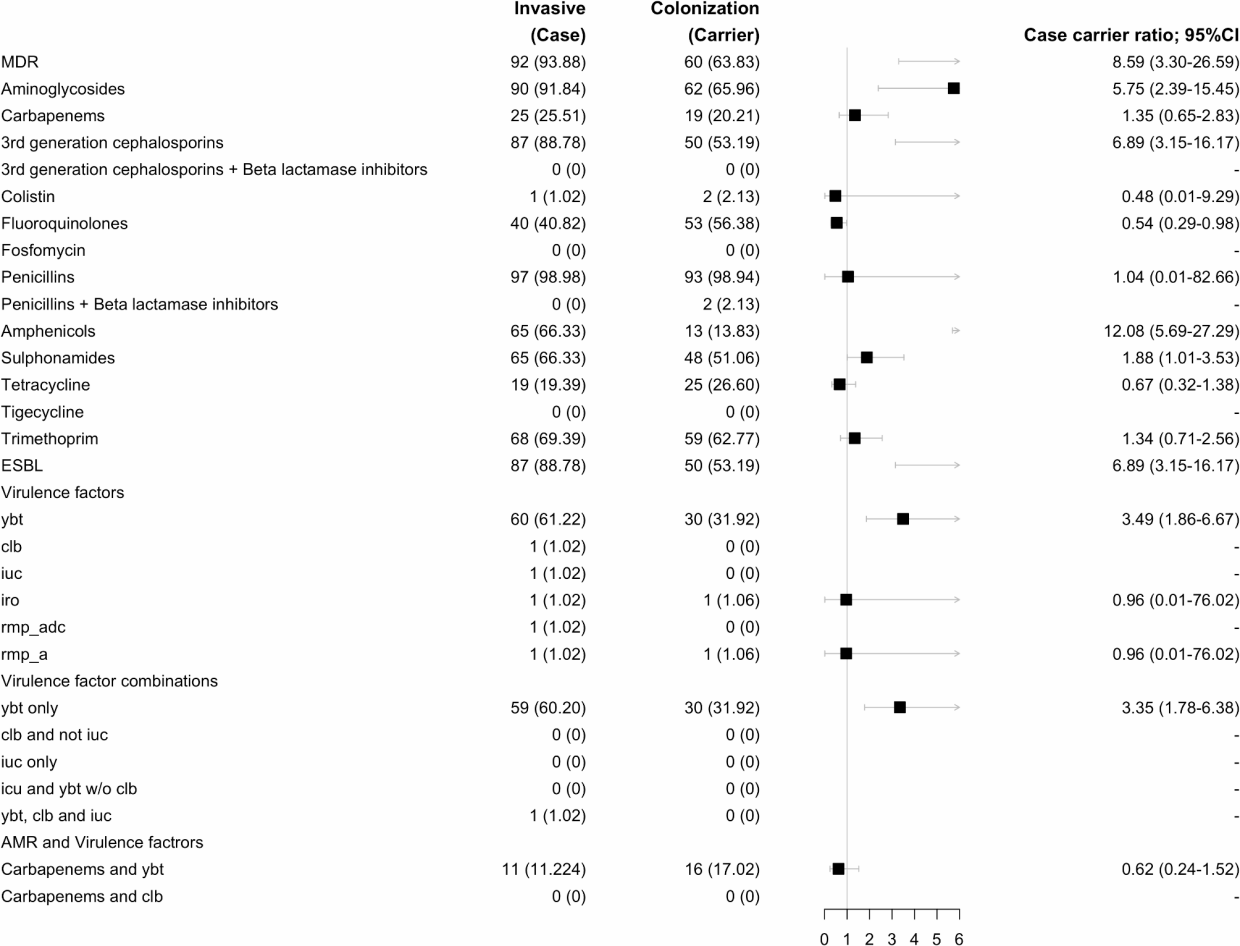
Forrest plot illustrating antimicrobial resistance and Virulence factors. *Ci* confidence interval, *clb* colibactin, *ESBL* extended spectrum β-lactamase, *iuc* aerobactin, *OR* odd ratio, *ST* sequence type, *ybt* yersiniabactin, P-values of < 0.05 are considered significant. Too few variables to calculate.

The prevalence of genes encoding for Yersiniabactin (ybt) production was higher in invasive (61%, 60/98) compared with colonizing isolates (32%, 30/94; OR 3.35; 95% CI 1.78–6.38; *p* < 0.001; Fig. 5). Salmochelin (iro) and rmpA2 were detected in only a single invasive and colonizing isolate, respectively while Colibactin (clb), aerobactin (iuc), and rmpADC were each detected exclusively in a single invasive isolate. The combination of ybt and carbapenem genes was prevalent in 11% (11/98) and 17% (16/94) of invasive and colonizing isolates, respectively.

The distribution of genes encoding for virulence was similar when stratifying by ST and K-loci; however, when stratifying by O-loci, the prevalence of genes encoding for ybt production was only significantly higher in O1/O2v1 (63%, 10/16 vs. 19%, 8/42; *p* = 0.003), O1/O2v2 (51%, 28/55 vs. 16%, 3/19; *p* = 0.008), and O4 (100%, 5/5 vs. 0%, 0/7; *p* = 0.001) invasive compared with colonizing isolates, respectively (Supplementary Table 2). 

### Antimicrobial resistance

Invasive compared with colonizing isolates had an 8.59 higher odds (95% CI 3.30–26.59; *p* < 0.001) of harbouring multi-drug resistance genes (94%, 92/98 vs. 64%, 60/94) including a 6.89 higher odds (95% CI 3.15–16.17; *p* < 0.001) of genes encoding for Extended-Spectrum Beta-Lactamase (ESBL; 89%, 87/98 vs. 53% (50/94) production (Fig. 5). The prevalence of genes encoding resistance to aminoglycosides (91%, 90/98 vs. 68%, 69/102; OR 5.75; 95% CI 2.39–15.45; *p* < 0.001), 3rd generation cephalosporins (89%, 87/98 vs. 53%, 50/94; OR 6.89; 95% CI 3.15–16.17; *p* < 0.001), and sulphonamides (66%, 65/98 vs. 51%, 48/94; OR 1.88; 95% CI 1.01–3.53; *p* = 0.040) were also higher in invasive compared with colonizing isolates. Conversely, the prevalence of genes encoding resistance to fluoroquinolones was lower in invasive disease (41%, 40/98) than in colonizing isolates (56%, 53/94; OR 0.54; 95% CI 0.29–0.98; *p* = 0.043).

When stratifying by ST, the prevalence of genes encoding carbapenems (59%, 10/17 vs. 0%, 0/8; *p* = 0.008) and tetracyclines (47%, 8/17 vs. 0%, 0/8; *p* = 0.026) resistance was higher in ST307 invasive compared with colonizing isolates, respectively (Supplementary Table 3). Conversely, the prevalence of genes encoding carbapenems (32%, 6/19 vs. 81%, 17/21; *p* = 0.003) and fluoroquinolones (21%, 4/19 vs. 71%, 15/21; *p* = 0.02) resistance was lower in ST17 invasive compared with colonizing isolates. Other differences included a higher prevalence of genes encoding 3rd generation cephalosporins resistance (100%, 19/19 vs. 67%, 14/21; *p* = 0.009) and ESBL production (100%, 19/19 vs. 14/21, 67%; *p* = 0.009) in ST17 invasive compared with colonizing isolates.

When stratifying by K-loci, the prevalence of genes encoding carbapenems (63%, 12/19 vs. 0%, 0/8; *p* = 0.003) and tetracyclines (44%, 8/19 vs. 0%, 0/8; *p* = 0.031) resistance was higher in KL102 invasive compared with colonizing isolates (Supplementary Table 3). Conversely, the prevalence of genes encoding carbapenems (28%, 6/21 vs. 85%, 17/20; *p* < 0.001) and fluoroquinolones (24%, 5/21 vs. 75%, 15/20) resistance was lower, while the prevalence of genes encoding for sulphonamides (100%, 21/21 vs. 80%, 16/20; *p* = 0.021) resistance was higher in KL25 invasive compared with colonizing isolates.

When stratifying by O-loci, invasive compared with colonizing O1/O2v2 isolates had a 23.53 higher odds (95% CI 2.54–1158.22; *p* < 0.001) of harbouring multi-drug resistance genes (98%, 54/55 vs. 68%, 13/19) including a 16.50 higher odds (95% CI 3.80–89.59; *p* < 0.001) of genes encoding for ESBL (93%, 51/55 vs. 42% (8/19) production. Furthermore, the prevalence of genes encoding for carbapenems (33%, 18/55 vs. 5%, 1/91; *p* = 0.030), third-generation cephalosporin (93%, 51/55 vs. 42%, 8/19; *p* < 0.001), and aminoglycoside (96%, 53/55 vs. 68%, 13/19; *p* = 0.003) resistance was higher in the O1/O2v2 invasive compared to colonizing isolates, respectively (Supplementary Table 3). Conversely, the prevalence of genes encoding carbapenems (35%, 6/17 vs. 85%, 17/20; *p* = 0.003) and fluoroquinolones (23%, 4/17 vs. 75%, 14/20; *p* = 0.003) resistance was lower in O5 invasive compared with colonizing isolates. Other differences included a lower prevalence of genes encoding for fluoroquinolones (19%, 3/16 vs. 67%, 28/42; *p* = 0.001) and tetracycline (13%, 2/16 vs. 52%, 22/42; *p* = 0.007) resistance in the O1/O2v1 invasive compared with colonizing isolates, and a higher prevalence of fluoroquinolones for O3b (80%, 4/5 vs. 0%, 0/4;*p* = 0.048) and O4 (80%, 4/5 vs. 0%, 0/4;*p* = 0.048) in the invasive isolates, respectively.

## Discussion

There was a notable genomic diversity of invasive disease compared with colonizing KPn strains in South African infants. There were fewer KPn strains identified among invasive cases, which exhibited a higher prevalence of MDR genes, including genes encoding for ESBL resistance production and yersiniabactin virulence compared with colonizing isolates. Notably, KL102 and O1/O2v2 strains were found to be more invasive with CCRs > 1, while ST1414, KL8, and O1/O2v1 strains were less invasive with low CCRs (< 1).

Although, we were unable to assess the CCRs for KPn strains identified exclusively in the invasive or colonizing isolates, ST39, KL149, and KL2 were detected in 9–30% of the invasive isolates only suggesting that these strains might be invasive and rarely carried. Conversely, ST10 and KL17 were found exclusively in 23% of the colonization isolates, suggesting a lower potential for causing disease. Larger and more robust studies will be needed to confirm the invasiveness of strains exclusively found in the invasive isolates. Notably, KL149 has not been reported widely and has mainly been detected in limited studies undertaken in South Africa^17,18^, Ghana^19^, and Poland^20^. Continued monitoring in our setting will thus be important to assess the potential of KL149 to increase disease burden.

The lower genomic diversity among invasive isolates compared to colonizing isolates suggests that certain KPn lineages may be more adapted to causing invasive disease; however, the capsular and lipopolysaccharide antigen alone may not be the only predictor of invasiveness. Notably, KL25 was detected in a similar proportion of invasive (21.4%) and colonization (21.3%) isolates, and is associated with invasive disease globally^21–24^. Moreover, KL25 invasive compared with colonizing isolates had a 4.08 higher odds of harbouring genes encoding for ESBL production, albeit not significant, underscoring the complexity of KPn pathogenicity. Differences in individual host susceptibility factors could influence the strain’s pathogenicity and account for the similar proportions of KL25 detected between the colonizing and invasive isolates; however, further investigations are warranted to better understand host-pathogen interactions.

Antimicrobial resistance mechanisms, including beta-lactamase production, often co-exist with virulence genes on mobile genetic elements, leading to the emergence of MDR, hypervirulent strains^25^. Notably, the invasive isolates exhibited a higher prevalence of MDR genes, including those responsible for ESBL production, as well as ybt genes that encode the siderophore yersiniabactin, which enhances iron acquisition during infection in iron-limited environments^25^. The combination of ESBL and ybt genes may have provided the invasive KPn strains with a dual advantage, allowing them to evade both host defenses and antibiotic treatment, thereby enhancing their survival and proliferation in challenging environments within the host, ultimately increasing their invasiveness.

The findings from our earlier study indicated that KPn only progressed to invasive disease in 5% of the colonized infants and that a different strain (ST2:KL9 and ST17:Kl25) caused disease compared to the one that colonized the two children (ST17:KL62 and ST307:KL102) in the cases with genomic data available^8^. Approximately 80% of the KPn isolates, across all sequenced samples included in our study, were only identified on colonizing or invasive disease isolates. This is consistent with a study in Ghana, that reported only 18% (*n* = 2/10) of the paired colonizing and bloodstream infections isolates to be the same KPn strain^10^. Other host factors, maternal carriage, or peripartum infection may be more closely linked to the development of invasive disease in the infant^7,26^ and further studies investigating the genomic relatedness of maternal colonizing KPn strains and those causing invasive neonatal disease are needed to better understand the link between maternal carriage, obstetric, and subsequent puerperal infections.

The high rates of MDR organisms, particularly ESBL-producing, detected across the invasive isolates highlight the significant challenges in treating these infections which are often associated with increased hospitalized stay and a high risk of death^27,28^. Global efforts are being directed toward developing vaccines to protect against the most invasive capsular and lipopolysaccharide KPn serotypes; however the geographic distribution of KL-antigens in complex and complicate vaccine development^7^. There are also concerns that there could be an increase in KPn disease caused by the capsular serotypes not included in the vaccine formulation after its introduction. While this study suggests that replacement by non-targeted KPn strains may be limited due to the detection of very few K-loci in both colonizing and invasive isolates, more extensive and robust studies are needed to better understand potential replacement dynamics. Nevertheless, only 41–53% of the K-loci identified in the invasive disease isolates were among the top 20 K-loci previously associated with neonatal sepsis in LMIC^2^ and Asia^29^, respectively. The wide variety of KPn strains causing childhood disease could indicate local adaptation mechanisms and distinct evolutionary pressures within different healthcare environments. Nevertheless, considering temporal and geographical variations in the dominant KPn strains will be important when formulating a universally effective vaccine, which remains a global challenge.

A limitation of our study is that it was not designed to investigate colonization and invasive disease with isolates collected from different hospitals within South Africa. The colonizing isolates were collected from one regional hospital only while the invasive isolates were collected from another 6 sites. This introduces potential bias (including regional bias) as risks for colonization and invasive disease vary with clinical care practices (i.e., intravenous lines and antibiotics exposure) that are not standardized across hospitals and accounted for in this study. Despite this limitation, phylogenetic analysis revealed that the colonizing isolates, even though collected from different hospitals, shared lineages with the invasive KpN isolates, suggesting that similar strains of KPn are circulating in different locations within South Africa. The KPn strains and clusters observed in this study may have been specific to the hospital, and it is uncertain whether the same colonizing strains would be detected elsewhere. A further limitation included that only a single colonizing and invasive isolate from each culture was selected for genomic characterization and current KPn strain present at a lower bacterial load might have been missed that could have influenced the calculated CCR. As a result, the few instances where a different KPn strain was detected at a later point could have been present from the outset. Further, newly acquired or persistent Kpn strains of a lower bacterial load may have also not been detected. Additionally, the sample size of invasive and colonizing isolates was small and may have contributed to the wide uncertainty bounds of our CCR estimates, including non-significance. Nevertheless, our study was hypothesis-generating and provides insight into South African high-risk strains potentially associated with increased invasive disease. Carefully designed case-control studies will be needed to validate the invasive potential of these KPn strains.

In conclusion, there was less genomic diversity amongst invasive disease (22 ST, 17 K-loci) than colonizing isolates (31 ST, 29 K-loci). Despite the lower diversity, the invasive KPn isolates exhibited heightened antimicrobial resistance and virulence compared with colonizing isolates. Notably, strains including KL102 and O1O2v2 had higher CCR, suggesting they may have greater potential for causing invasive disease. Further research is needed to confirm the invasiveness of KL102, O1O2v2, and other strains identified exclusively in the invasive isolates, as well as to determine the generalizability of our findings. Identifying strains with a higher invasive disease potential can inform the selection of antigens in the development of vaccines to protect against the most invasive capsular and lipopolysaccharide serotypes.

## Electronic supplementary material

Below is the link to the electronic supplementary material.

Supplementary Material 1-  Table 2 missing from link. Have uploaded an updated version with sup Table 2 now included. 
