# Supplementary material for: Genomic relatedness of colonizing and invasive disease Klebsiella pneumoniae isolates in South African infants
Source: Sci Rep. 2025 Mar 7;15:8043. doi: 10.1038/s41598-025-92517-4 (PMC11889247; doi:10.1038/s41598-025-92517-4)
Supplement: Supplementary file 1 — Supplementary Material 1- Table 2 missing from link. Have uploaded an updated version with sup Table 2 now included. [file 41598_2025_92517_MOESM1_ESM.docx]

**Supplementary:**

**Genomic relatedness of colonizing and invasive disease *Klebsiella pneumoniae* isolates in South African infants**

*Courtney P. Olwagen^1^, Alane Izu^1^, Shama Khan^1^, Lara Van der Merwe^1^, Nicholas J. Dean^1^, Fikile C. Mabena^1,6^, Stephanie Jones^1^, Gaurav Kwatra^1,2,3^, Lubomira Andrew^4^, Urvi Rajyaguru^4^, Robert G. K. Donald^4^, Raphael Simon^4^, Mohamed Said^5^, Firdose L. Nakwa^6^,* *Jeannette Wadula^7^, Renate Strehlau^8^, Anika M. van Niekerk^9^, Niree Naidoo^7^, Yogandree Ramsamy^10,11^, Sithembiso C. Velaphi^6^, Ziyaad Dangor^1^, and Shabir A. Madhi^1,12^*

**Supplementary Table 1**: Case carrier ratio of Klebsiella pneumoniae, by location centre

|  | **TMRH** | **All invasive sites** | | **CHBAH** | | **PMMH** | | **RMMCH** | | **TMHC** | |
| --- | --- | --- | --- | --- | --- | --- | --- | --- | --- | --- | --- |
|  | **Colonization** | **Invasive** | **CCR (95% CI)** | **Invasive** | **CCR (95% CI)** | **Invasive** | **CCR (95% CI)** | **Invasive** | **CCR (95% CI)** | **Invasive** | **CCR (95% CI)** |
| **Sequence types, n(%)** |  |  |  |  |  |  |  |  |  |  |  |
| ST101 | 22 (23.4) | 0 (0) | - | 0 (0) | - | 0 (0) | - | 0 (0) | - | 0 (0) | - |
| ST1026 | 1 (1.1) | 0 (0) | - | 0 (0) | - | 0 (0) | - | 0 (0) | - | 0 (0) | - |
| ST1119 | 1 (1.1) | 0 (0) | - | 0 (0) | - | 0 (0) | - | 0 (0) | - | 0 (0) | - |
| ST1263 | 1 (1.1) | 0 (0) | - | 0 (0) | - | 0 (0) | - | 0 (0) | - | 0 (0) | - |
| ST13 | 2 (2.1) | 3 (3.1) | 1.45 (0.24-11.22) | 3 (4.1) | 1.94 (0.31-15.05) | 0 (0) | - | 0 (0) | - | 0 (0) | - |
| ST133 | 3 (3.2) | 0 (0) | - | 0 (0) | - | 0 (0) | - | 0 (0) | - | 0 (0) | - |
| ST1380 | 2 (2.1) | 0 (0) | - | 0 (0) | - | 0 (0) | - | 0 (0) | - | 0 (0) | - |
| ST14 | 0 (0) | 7 (7.1) | - | 5 (6.8) | - | 0 (0) | - | 2 (22.2) | - | 0 (0) | - |
| ST1401 | 1 (1.1) | 0 (0) | - | 0 (0) | - | 0 (0) | - | 0 (0) | - | 0 (0) | - |
| ST1414 | 6 (6.4) | 1 (1) | 0.15 (0.01-0.91)* | 0 (0) | - | 1 (16.7) | 2.93 (0.14-22.61) | 0 (0) | - | 0 (0) | - |
| ST1429 | 1 (1.1) | 0 (0) | - | 0 (0) | - | 0 (0) | - | 0 (0) | - | 0 (0) | - |
| ST15 | 3 (3.2) | 1 (1) | 0.31 (0.02-2.49) | 1 (1.4) | 0.42 (0.02-3.32) | 0 (0) | - | 0 (0) | - | 0 (0) | - |
| ST152 | 0 (0) | 5 (5.1) | - | 2 (2.7) | - | 0 (0) | - | 0 (0) | - | 3 (33.3) | - |
| ST1552 | 0 (0) | 2 (2) | - | 0 (0) | - | 0 (0) | - | 2 (22.2) | - | 0 (0) | - |
| ST163 | 1 (1.1) | 0 (0) | - | 0 (0) | - | 0 (0) | - | 0 (0) | - | 0 (0) | - |
| ST1694 | 1 (1.1) | 0 (0) | - | 0 (0) | - | 0 (0) | - | 0 (0) | - | 0 (0) | - |
| ST17 | 21 (22.3) | 19 (19.4) | 0.84 (0.41-1.68) | 19 (25.7) | 1.2 (0.59-2.45) | 0 (0) | - | 0 (0) | - | 0 (0) | - |
| ST1873 | 0 (0) | 2 (2) | - | 2 (2.7) | - | 0 (0) | - | 0 (0) | - | 0 (0) | - |
| ST193 | 0 (0) | 171 (1) | - | 0 (0) | - | 0 (0) | - | 0 (0) | - | 1 (11.1) | - |
| ST1999 | 1 (1.1) | 0 (0) | - | 0 (0) | - | 0 (0) | - | 0 (0) | - | 0 (0) | - |
| ST20 | 1 (1.1) | 0 (0) | - | 0 (0) | - | 0 (0) | - | 0 (0) | - | 0 (0) | - |
| ST2039 | 0 (0) | 1 (1) | - | 1 (1.4) | - | 0 (0) | - | 0 (0) | - | 0 (0) | - |
| ST22 | 2 (2.1) | 0 (0) | - | 0 (0) | - | 0 (0) | - | 0 (0) | - | 0 (0) | - |
| ST231 | 0 (0) | 1 (1) | - | 1 (1.4) | - | 0 (0) | - | 0 (0) | - | 0 (0) | - |
| ST2441 | 0 (0) | 1 (1) | - | 1 (1.4) | - | 0 (0) | - | 0 (0) | - | 0 (0) | - |
| ST25 | 0 (0) | 2 (2) | - | 0 (0) | - | 1 (16.7) | - | 0 (0) | - | 1 (11.1) | - |
| ST252 | 1 (1.1) | 1 (1) | 0.96 (0.04-24.48) | 0 (0) | - | 0 (0) | - | 0 (0) | - | 1 (11.1) | 11.62 (0.43-313.58) |
| ST307 | 8 (8.5) | 17 (17.3) | 2.1 (0.87-5.42) | 15 (20.3) | 2.73 (1.11-7.17)* | 1 (16.7) | 2.15 (0.11-15.71) | 0 (0) | - | 1 (11.1) | 1.34 (0.07-8.81) |
| ST309 | 1 (1.1) | 0 (0) | - | 0 (0) | - | 0 (0) | - | 0 (0) | - | 0 (0) | - |
| ST336 | 0 (0) | 1 (1) | - | 0 (0) | - | 0 (0) | - | 1 (11.1) | - | 0 (0) | - |
| ST34 | 1 (1.1) | 0 (0) | - | 0 (0) | - | 0 (0) | - | 0 (0) | - | 0 (0) | - |
| ST35 | 1 (1.1) | 1 (1) | 0.96 (0.04-24.48) | 1 (1.4) | 1.27 (0.05-32.58) | 0 (0) | - | 0 (0) | - | 0 (0) | - |
| ST353 | 1 (1.1) | 4 (4.1) | 3.96 (0.57-78.2) | 0 (0) | - | 0 (0) | - | 4 (44.4) | 74.4 (9.08-1615.68)* | 0 (0) | - |
| ST3688 | 1 (1.1) | 0 (0) | - | 0 (0) | - | 0 (0) | - | 0 (0) | - | 0 (0) | - |
| ST37 | 1 (1.1) | 0 (0) | - | 0 (0) | - | 0 (0) | - | 0 (0) | - | 0 (0) | - |
| ST39 | 0 (0) | 22 (22.5) | - | 20 (27.0) | - | 3 (50) | - | 0 (0) | - | 0 (0) | - |
| ST3985 | 1 (1.1) | 0 (0) | - | 0 (0) | - | 0 (0) | - | 0 (0) | - | 0 (0) | - |
| ST416 | 1 (1.1) | 0 (0) | - | 0 (0) | - | 0 (0) | - | 0 (0) | - | 0 (0) | - |
| ST4291 | 0 (0) | 2 (2) | - | 2 (2.7) | - | 0 (0) | - | 0 (0) | - | 0 (0) | - |
| ST460 | 1 (1.1) | 0 (0) | - | 0 (0) | - | 0 (0) | - | 0 (0) | - | 0 (0) | - |
| ST461 | 1 (1.1) | 0 (0) | - | 0 (0) | - | 0 (0) | - | 0 (0) | - | 0 (0) | - |
| ST502 | 4 (4.3) | 0 (0) | - | 0 (0) | - | 0 (0) | - | 0 (0) | - | 0 (0) | - |
| ST607 | 0 (0) | 2 (2) | - | 1 (1.4) | - | 0 (0) | - | 0 (0) | - | 1 (11.1) | - |
| ST611 | 1 (1.1) | 0 (0) | - | 0 (0) | - | 0 (0) | - | 0 (0) | - | 0 (0) | - |
| ST987 | 0 (0) | 1 (1) | - | 0 (0) | - | 0 (0) | - | 0 (0) | - | 1 (11.1) | - |
| **K-loci, n(%)** |  |  |  |  |  |  |  |  |  |  |  |
| KL10 | 2 (2.1) | 0 (0) | - | 0 (0) | - | 0 (0) | - | 0 (0) | - | 0 (0) | - |
| KL102 | 8 (8.5) | 19 (19.4) | 2.59 (1.11-6.58)* | 17 (23.9) | 3.21 (1.33-8.32)* | 1 (16.7) | 2.15 (0.11-15.71) | 0 (0) | - | 1 (11.1) | 1.34 (0.07-8.81) |
| KL103 | 1 (1.1) | 0 (0) | - | 0 (0) | - | 0 (0) | - | 0 (0) | - | 0 (0) | - |
| KL106 | 1 (1.1) | 0 (0) | - | 0 (0) | - | 0 (0) | - | 0 (0) | - | 0 (0) | - |
| KL108 | 0 (0) | 1 (1) | - | 1 (1.4) | - | 0 (0) | - | 0 (0) | - | 0 (0) | - |
| KL110 | 2 (2.1) | 4 (4.1) | 1.96 (0.37-14.37) | 0 (0) | - | 0 (0) | - | 4 (44.4) | 36.8 (5.85-319.45)* | 0 (0) | - |
| KL112 | 0 (0) | 1 (1) | - | 1 (1.4) | - | 0 (0) | - | 0 (0) | - | 0 (0) | - |
| KL116 | 2 (2.1) | 0 (0) | - | 0 (0) | - | 0 (0) | - | 0 (0) | - | 0 (0) | - |
| KL12 | 1 (1.1) | 0 (0) | - | 0 (0) | - | 0 (0) | - | 0 (0) | - | 0 (0) | - |
| KL125 | 1 (1.1) | 0 (0) | - | 0 (0) | - | 0 (0) | - | 0 (0) | - | 0 (0) | - |
| KL128 | 1 (1.1) | 0 (0) | - | 0 (0) | - | 0 (0) | - | 0 (0) | - | 0 (0) | - |
| KL140 | 1 (1.1) | 0 (0) | - | 0 (0) | - | 0 (0) | - | 0 (0) | - | 0 (0) | - |
| KL142 | 1 (1.1) | 0 (0) | - | 0 (0) | - | 0 (0) | - | 0 (0) | - | 0 (0) | - |
| KL149 | 0 (0) | 28 (28.6) | - | 22 (29.7) | - | 3 (50) | - | 0 (0) | - | 3 (33.3) | - |
| KL15 | 5 (5.3) | 0 (0) | - | 0 (0) | - | 0 (0) | - | 0 (0) | - | 0 (0) | - |
| KL158 | 1 (1.1) | 0 (0) | - | 0 (0) | - | 0 (0) | - | 0 (0) | - | 0 (0) | - |
| KL16 | 0 (0) | 1 (1) | - | 1 (1.4) | - | 0 (0) | - | 0 (0) | - | 0 (0) | - |
| KL17 | 22 (23.4) | 0 (0) | - | 0 (0) | - | 0 (0) | - | 0 (0) | - | 0 (0) | - |
| KL2 | 0 (0) | 9 (9.2) | - | 5 (6.8) | - | 1 (16.7) | - | 2 (22.2) | - | 1 (11.1) | - |
| KL24 | 4 (4.3) | 0 (0) | - | 0 (0) | - | 0 (0) | - | 0 (0) | - | 0 (0) | - |
| KL25 | 20 (21.3) | 21 (21.4) | 1.01 (0.5-2.02) | 17 (23) | 1.1 (0.53-2.3) | 0 (0) | - | 3 (33.3) | 1.85 (0.37-7.69) | 1 (11.1) | 0.46 (0.02-2.74) |
| KL28 | 0 (0) | 2 (2) | - | 2 (2.7) | - | 0 (0) | - | 0 (0) | - | 0 (0) | - |
| KL3 | 2 (2.1) | 3 (3.1) | 1.45 (0.24-11.22) | 3 (4.1) | 1.94 (0.31-15.05) | 0 (0) | - | 0 (0) | - | 0 (0) | - |
| KL30 | 1 (1.1) | 2 (2) | 1.94 (0.18-42.11) | 1 (1.4) | 1.27 (0.05-32.58) | 0 (0) | - | 0 (0) | - | 1 (11.1) | 11.62 (0.43-313.58) |
| KL38 | 1 (1.1) | 0 (0) | - | 0 (0) | - | 0 (0) | - | 0 (0) | - | 0 (0) | - |
| KL39 | 1 (1.1) | 0 (0) | - | 0 (0) | - | 0 (0) | - | 0 (0) | - | 0 (0) | - |
| KL42 | 1 (1.1) | 0 (0) | - | 0 (0) | - | 0 (0) | - | 0 (0) | - | 0 (0) | - |
| KL45 | 1 (1.1) | 0 (0) | - | 0 (0) | - | 0 (0) | - | 0 (0) | - | 0 (0) | - |
| KL46 | 0 (0) | 1 (1) | - | 0 (0) | - | 0 (0) | - | 0 (0) | - | 1 (11.1) | - |
| KL51 | 1 (1.1) | 1 (1) | 0.96 (0.04-24.48) | 0 (0) | - | 0 (0) | - | 0 (0) | - | 1 (11.1) | 11.62 (0.43-313.58) |
| KL60 | 1 (1.1) | 0 (0) | - | 0 (0) | - | 0 (0) | - | 0 (0) | - | 0 (0) | - |
| KL61 | 1 (1.1) | 0 (0) | - | 0 (0) | - | 0 (0) | - | 0 (0) | - | 0 (0) | - |
| KL62 | 1 (1.1) | 3 (3.1) | 2.94 (0.37-59.96) | 3 (4.1) | 3.93 (0.49-80.36) | 0 (0) | - | 0 (0) | - | 0 (0) | - |
| KL63 | 1 (1.1) | 0 (0) | - | 0 (0) | - | 0 (0) | - | 0 (0) | - | 0 (0) | - |
| KL7 | 0 (0) | 1 (1) | - | 1 (1.4) | - | 0 (0) | - | 0 (0) | - | 0 (0) | - |
| KL8 | 7 (7.4) | 1 (1) | 0.13 (0.01-0.74)* | 0 (0) | - | 1 (16.7) | 2.49 (0.12-18.58) | 0 (0) | - | 0 (0) | - |
| KL9 | 2 (2.1) | 0 (0) | - | 0 (0) | - | 0 (0) | - | 0 (0) | - | 0 (0) | - |
| **O-loci, n(%)** |  |  |  |  |  |  |  |  |  |  |  |
| O1/O2v1 | 42 (44.7) | 16 (16.3) | 0.24 (0.12-0.47)* | 10 (13.5) | 0.19 (0.08-0.41)* | 1 (16.7) | 0.25 (0.01-1.61) | 4 (44.4) | 0.99 (0.23-3.97) | 1 (11.1) | 0.15 (0.01-0.89)* |
| O1/O2v2 | 19 (20.2) | 55 (56.1) | 5.05 (2.7-9.78)* | 46 (62.2) | 6.48 (3.31-13.17)* | 5 (83.3) | 19.74 (2.96-390.26)* | 0 (0) | - | 4 (44.4) | 3.16 (0.72-13.08) |
| O12 | 1 (1.1) | 0 (0) | - | 0 (0) | - | 0 (0) | - | 0 (0) | - | 0 (0) | - |
| O3/O3a | 1 (1.1) | 0 (0) | - | 0 (0) | - | 0 (0) | - | 0 (0) | - | 0 (0) | - |
| O3b | 4 (4.3) | 5 (5.1) | 1.21 (0.31-5.02) | 0 (0) | - | 0 (0) | - | 4 (44.4) | 18 (3.42-101.15)* | 1 (11.1) | 2.81 (0.14-22.13) |
| O4 | 7 (7.4) | 5 (5.1) | 0.67 (0.19-2.17) | 2 (2.7) | 0.35 (0.05-1.48) | 0 (0) | - | 0 (0) | - | 3 (33.3) | 6.21 (1.13-29.74)* |
| O5 | 20 (21.3) | 17 (17.3) | 0.78 (0.37-1.59) | 16 (21.6) | 1.02 (0.48-2.14) | 0 (0) | - | 1 (11.1) | 0.46 (0.02-2.74) | 0 (0) | - |
| **O-type, n (%)** |  |  |  |  |  |  |  |  |  |  |  |
| O1ab | 47 (50) | 45 (45.9) | 0.88 (0.5-1.56) | 33 (44.6) | 0.85 (0.46-1.56) | 4 (66.7) | 2 (0.37-14.93) | 4 (44.4) | 0.8 (0.19-3.2) | 4 (44.4) | 0.8 (0.19-3.2) |
| O12 | 1 (1.1) | 0 (0) | - | 0 (0) | - | 0 (0) | - | 0 (0) | - | 0 (0) | - |
| O2a | 3 (3.2) | 2 (2) | 0.63 (0.08-3.9) | 2 (2.7) | 0.84 (0.11-5.21) | 0 (0) | - | 0 (0) | - | 0 (0) | - |
| O2afg | 11 (11.7) | 24 (24.5) | 2.45 (1.15-5.52)* | 21 (28.4) | 2.99 (1.36-6.91)* | 2 (33.3) | 3.77 (0.48-21.85) | 0 (0) | - | 1 (11.1) | 0.94 (0.05-5.9) |
| O3/O3a | 1 (1.1) | 0 (0) | - | 0 (0) | - | 0 (0) | - | 0 (0) | - | 0 (0) | - |
| O3b | 4 (4.3) | 5 (5.1) | 1.21 (0.31-5.02) | 0 (0) | - | 0 (0) | - | 4 (44.4) | 18 (3.42-101.15)* | 1 (11.1) | 2.81 (0.14-22.13) |
| O4 | 7 (7.4) | 5 (5.1) | 0.67 (0.19-2.17) | 2 (2.7) | 0.35 (0.05-1.48) | 0 (0) | - | 0 (0) | - | 3 (33.3) | 6.21 (1.13-29.74)* |
| O5 | 20 (21.3) | 17 (17.3) | 0.78 (0.37-1.59) | 16 (21.6) | 1.02 (0.48-2.14) | 0 (0) | - | 1 (11.1) | 0.46 (0.02-2.74) | 0 (0) | - |

Abbreviations: CCR – case-carrier ration, CHBAH - Chris Hani Baragwanath Academic Hospital, CI – confidence intervals, TMRH - Thelle Moerane Regional Hospital, PMMCH - Prince Mshiyeni Memorial Hospital, RMMCH - Rahima Moosa Mother and Child Hospital, TALM - Tshwane Academic Laboratory Network.

As a proxy for the relative invasiveness of KPn isolates, the invasiveness was calculated by measuring the case carrier ratio of the odds (CCR) for each ST, K-locus, O-locus, and O-antigen type by dividing the number of isolates with the genetic characteristic of interest by the number of isolates without the characteristic of interest.

- Too few observations to calculate CCR

**Supplementary Figure 1**: Sequence types (ST), by location centre

**Supplementary Figure 2:** K-loci, by location centre

**Supplementary Figure 3:** O-loci, by location centre

**Supplementary Figure 5:** O-antigen type, by location centre

**Supplementary Table 2**: virulence, by sequence types (ST), K-loci, O-loci, and O-antigen type

|  | **Virulence factors** | **Colonization (n=94)** | **Invasive (n=98)** | **P-value** |  |
| --- | --- | --- | --- | --- | --- |
| **Sequence types, n (%)** | |  |  |  |  |
| ST101 | ybt | 0/22 (0) | 0/0 (0) | p>0.999 |  |
| ST101 | clb | 0/22 (0) | 0/0 (0) | p>0.999 |  |
| ST101 | iuc | 0/22 (0) | 0/0 (0) | p>0.999 |  |
| ST101 | iro | 0/22 (0) | 0/0 (0) | p>0.999 |  |
| ST101 | rmp_adc | 0/22 (0) | 0/0 (0) | p>0.999 |  |
| ST101 | rmp_a2 | 0/22 (0) | 0/0 (0) | p>0.999 |  |
| ST101 | ybt only | 0/22 (0) | 0/0 (0) | p>0.999 |  |
| ST101 | clb and not iuc | 0/22 (0) | 0/0 (0) | p>0.999 |  |
| ST101 | iuc only | 0/22 (0) | 0/0 (0) | p>0.999 |  |
| ST101 | icu and ybt w/o clb | 0/22 (0) | 0/0 (0) | p>0.999 |  |
| ST101 | ybt, clb and iuc | 0/22 (0) | 0/0 (0) | p>0.999 |  |
| ST1026 | ybt | 0/1 (0) | 0/0 (0) | p>0.999 |  |
| ST1026 | clb | 0/1 (0) | 0/0 (0) | p>0.999 |  |
| ST1026 | iuc | 0/1 (0) | 0/0 (0) | p>0.999 |  |
| ST1026 | iro | 0/1 (0) | 0/0 (0) | p>0.999 |  |
| ST1026 | rmp_adc | 0/1 (0) | 0/0 (0) | p>0.999 |  |
| ST1026 | rmp_a2 | 0/1 (0) | 0/0 (0) | p>0.999 |  |
| ST1026 | ybt only | 0/1 (0) | 0/0 (0) | p>0.999 |  |
| ST1026 | clb and not iuc | 0/1 (0) | 0/0 (0) | p>0.999 |  |
| ST1026 | iuc only | 0/1 (0) | 0/0 (0) | p>0.999 |  |
| ST1026 | icu and ybt w/o clb | 0/1 (0) | 0/0 (0) | p>0.999 |  |
| ST1026 | ybt, clb and iuc | 0/1 (0) | 0/0 (0) | p>0.999 |  |
| ST1119 | ybt | 0/1 (0) | 0/0 (0) | p>0.999 |  |
| ST1119 | clb | 0/1 (0) | 0/0 (0) | p>0.999 |  |
| ST1119 | iuc | 0/1 (0) | 0/0 (0) | p>0.999 |  |
| ST1119 | iro | 0/1 (0) | 0/0 (0) | p>0.999 |  |
| ST1119 | rmp_adc | 0/1 (0) | 0/0 (0) | p>0.999 |  |
| ST1119 | rmp_a2 | 0/1 (0) | 0/0 (0) | p>0.999 |  |
| ST1119 | ybt only | 0/1 (0) | 0/0 (0) | p>0.999 |  |
| ST1119 | clb and not iuc | 0/1 (0) | 0/0 (0) | p>0.999 |  |
| ST1119 | iuc only | 0/1 (0) | 0/0 (0) | p>0.999 |  |
| ST1119 | icu and ybt w/o clb | 0/1 (0) | 0/0 (0) | p>0.999 |  |
| ST1119 | ybt, clb and iuc | 0/1 (0) | 0/0 (0) | p>0.999 |  |
| ST1263 | ybt | 0/1 (0) | 0/0 (0) | p>0.999 |  |
| ST1263 | clb | 0/1 (0) | 0/0 (0) | p>0.999 |  |
| ST1263 | iuc | 0/1 (0) | 0/0 (0) | p>0.999 |  |
| ST1263 | iro | 0/1 (0) | 0/0 (0) | p>0.999 |  |
| ST1263 | rmp_adc | 0/1 (0) | 0/0 (0) | p>0.999 |  |
| ST1263 | rmp_a2 | 0/1 (0) | 0/0 (0) | p>0.999 |  |
| ST1263 | ybt only | 0/1 (0) | 0/0 (0) | p>0.999 |  |
| ST1263 | clb and not iuc | 0/1 (0) | 0/0 (0) | p>0.999 |  |
| ST1263 | iuc only | 0/1 (0) | 0/0 (0) | p>0.999 |  |
| ST1263 | icu and ybt w/o clb | 0/1 (0) | 0/0 (0) | p>0.999 |  |
| ST1263 | ybt, clb and iuc | 0/1 (0) | 0/0 (0) | p>0.999 |  |
| ST13 | ybt | 2/2 (100) | 3/3 (100) | p>0.999 |  |
| ST13 | clb | 0/2 (0) | 0/3 (0) | p>0.999 |  |
| ST13 | iuc | 0/2 (0) | 0/3 (0) | p>0.999 |  |
| ST13 | iro | 0/2 (0) | 0/3 (0) | p>0.999 |  |
| ST13 | rmp_adc | 0/2 (0) | 0/3 (0) | p>0.999 |  |
| ST13 | rmp_a2 | 0/2 (0) | 0/3 (0) | p>0.999 |  |
| ST13 | ybt only | 2/2 (100) | 3/3 (100) | p>0.999 |  |
| ST13 | clb and not iuc | 0/2 (0) | 0/3 (0) | p>0.999 |  |
| ST13 | iuc only | 0/2 (0) | 0/3 (0) | p>0.999 |  |
| ST13 | icu and ybt w/o clb | 0/2 (0) | 0/3 (0) | p>0.999 |  |
| ST13 | ybt, clb and iuc | 0/2 (0) | 0/3 (0) | p>0.999 |  |
| ST133 | ybt | 2/3 (66.667) | 0/0 (0) | p>0.999 |  |
| ST133 | clb | 0/3 (0) | 0/0 (0) | p>0.999 |  |
| ST133 | iuc | 0/3 (0) | 0/0 (0) | p>0.999 |  |
| ST133 | iro | 0/3 (0) | 0/0 (0) | p>0.999 |  |
| ST133 | rmp_adc | 0/3 (0) | 0/0 (0) | p>0.999 |  |
| ST133 | rmp_a2 | 0/3 (0) | 0/0 (0) | p>0.999 |  |
| ST133 | ybt only | 2/3 (66.667) | 0/0 (0) | p>0.999 |  |
| ST133 | clb and not iuc | 0/3 (0) | 0/0 (0) | p>0.999 |  |
| ST133 | iuc only | 0/3 (0) | 0/0 (0) | p>0.999 |  |
| ST133 | icu and ybt w/o clb | 0/3 (0) | 0/0 (0) | p>0.999 |  |
| ST133 | ybt, clb and iuc | 0/3 (0) | 0/0 (0) | p>0.999 |  |
| ST1380 | ybt | 0/2 (0) | 0/0 (0) | p>0.999 |  |
| ST1380 | clb | 0/2 (0) | 0/0 (0) | p>0.999 |  |
| ST1380 | iuc | 0/2 (0) | 0/0 (0) | p>0.999 |  |
| ST1380 | iro | 0/2 (0) | 0/0 (0) | p>0.999 |  |
| ST1380 | rmp_adc | 0/2 (0) | 0/0 (0) | p>0.999 |  |
| ST1380 | rmp_a2 | 0/2 (0) | 0/0 (0) | p>0.999 |  |
| ST1380 | ybt only | 0/2 (0) | 0/0 (0) | p>0.999 |  |
| ST1380 | clb and not iuc | 0/2 (0) | 0/0 (0) | p>0.999 |  |
| ST1380 | iuc only | 0/2 (0) | 0/0 (0) | p>0.999 |  |
| ST1380 | icu and ybt w/o clb | 0/2 (0) | 0/0 (0) | p>0.999 |  |
| ST1380 | ybt, clb and iuc | 0/2 (0) | 0/0 (0) | p>0.999 |  |
| ST14 | ybt | 0/0 (0) | 7/7 (100) | p>0.999 |  |
| ST14 | clb | 0/0 (0) | 0/7 (0) | p>0.999 |  |
| ST14 | iuc | 0/0 (0) | 0/7 (0) | p>0.999 |  |
| ST14 | iro | 0/0 (0) | 0/7 (0) | p>0.999 |  |
| ST14 | rmp_adc | 0/0 (0) | 0/7 (0) | p>0.999 |  |
| ST14 | rmp_a2 | 0/0 (0) | 0/7 (0) | p>0.999 |  |
| ST14 | ybt only | 0/0 (0) | 7/7 (100) | p>0.999 |  |
| ST14 | clb and not iuc | 0/0 (0) | 0/7 (0) | p>0.999 |  |
| ST14 | iuc only | 0/0 (0) | 0/7 (0) | p>0.999 |  |
| ST14 | icu and ybt w/o clb | 0/0 (0) | 0/7 (0) | p>0.999 |  |
| ST14 | ybt, clb and iuc | 0/0 (0) | 0/7 (0) | p>0.999 |  |
| ST1401 | ybt | 0/1 (0) | 0/0 (0) | p>0.999 |  |
| ST1401 | clb | 0/1 (0) | 0/0 (0) | p>0.999 |  |
| ST1401 | iuc | 0/1 (0) | 0/0 (0) | p>0.999 |  |
| ST1401 | iro | 0/1 (0) | 0/0 (0) | p>0.999 |  |
| ST1401 | rmp_adc | 0/1 (0) | 0/0 (0) | p>0.999 |  |
| ST1401 | rmp_a2 | 0/1 (0) | 0/0 (0) | p>0.999 |  |
| ST1401 | ybt only | 0/1 (0) | 0/0 (0) | p>0.999 |  |
| ST1401 | clb and not iuc | 0/1 (0) | 0/0 (0) | p>0.999 |  |
| ST1401 | iuc only | 0/1 (0) | 0/0 (0) | p>0.999 |  |
| ST1401 | icu and ybt w/o clb | 0/1 (0) | 0/0 (0) | p>0.999 |  |
| ST1401 | ybt, clb and iuc | 0/1 (0) | 0/0 (0) | p>0.999 |  |
| ST1414 | ybt | 6/6 (100) | 0/1 (0) | p=0.143 |  |
| ST1414 | clb | 0/6 (0) | 0/1 (0) | p>0.999 |  |
| ST1414 | iuc | 0/6 (0) | 0/1 (0) | p>0.999 |  |
| ST1414 | iro | 0/6 (0) | 0/1 (0) | p>0.999 |  |
| ST1414 | rmp_adc | 0/6 (0) | 0/1 (0) | p>0.999 |  |
| ST1414 | rmp_a2 | 0/6 (0) | 0/1 (0) | p>0.999 |  |
| ST1414 | ybt only | 6/6 (100) | 0/1 (0) | p=0.143 |  |
| ST1414 | clb and not iuc | 0/6 (0) | 0/1 (0) | p>0.999 |  |
| ST1414 | iuc only | 0/6 (0) | 0/1 (0) | p>0.999 |  |
| ST1414 | icu and ybt w/o clb | 0/6 (0) | 0/1 (0) | p>0.999 |  |
| ST1414 | ybt, clb and iuc | 0/6 (0) | 0/1 (0) | p>0.999 |  |
| ST1429 | ybt | 0/1 (0) | 0/0 (0) | p>0.999 |  |
| ST1429 | clb | 0/1 (0) | 0/0 (0) | p>0.999 |  |
| ST1429 | iuc | 0/1 (0) | 0/0 (0) | p>0.999 |  |
| ST1429 | iro | 0/1 (0) | 0/0 (0) | p>0.999 |  |
| ST1429 | rmp_adc | 0/1 (0) | 0/0 (0) | p>0.999 |  |
| ST1429 | rmp_a2 | 0/1 (0) | 0/0 (0) | p>0.999 |  |
| ST1429 | ybt only | 0/1 (0) | 0/0 (0) | p>0.999 |  |
| ST1429 | clb and not iuc | 0/1 (0) | 0/0 (0) | p>0.999 |  |
| ST1429 | iuc only | 0/1 (0) | 0/0 (0) | p>0.999 |  |
| ST1429 | icu and ybt w/o clb | 0/1 (0) | 0/0 (0) | p>0.999 |  |
| ST1429 | ybt, clb and iuc | 0/1 (0) | 0/0 (0) | p>0.999 |  |
| ST15 | ybt | 0/3 (0) | 1/1 (100) | p=0.25 |  |
| ST15 | clb | 0/3 (0) | 0/1 (0) | p>0.999 |  |
| ST15 | iuc | 0/3 (0) | 0/1 (0) | p>0.999 |  |
| ST15 | iro | 0/3 (0) | 0/1 (0) | p>0.999 |  |
| ST15 | rmp_adc | 0/3 (0) | 0/1 (0) | p>0.999 |  |
| ST15 | rmp_a2 | 0/3 (0) | 0/1 (0) | p>0.999 |  |
| ST15 | ybt only | 0/3 (0) | 1/1 (100) | p=0.25 |  |
| ST15 | clb and not iuc | 0/3 (0) | 0/1 (0) | p>0.999 |  |
| ST15 | iuc only | 0/3 (0) | 0/1 (0) | p>0.999 |  |
| ST15 | icu and ybt w/o clb | 0/3 (0) | 0/1 (0) | p>0.999 |  |
| ST15 | ybt, clb and iuc | 0/3 (0) | 0/1 (0) | p>0.999 |  |
| ST152 | ybt | 0/0 (0) | 5/5 (100) | p>0.999 |  |
| ST152 | clb | 0/0 (0) | 0/5 (0) | p>0.999 |  |
| ST152 | iuc | 0/0 (0) | 0/5 (0) | p>0.999 |  |
| ST152 | iro | 0/0 (0) | 0/5 (0) | p>0.999 |  |
| ST152 | rmp_adc | 0/0 (0) | 0/5 (0) | p>0.999 |  |
| ST152 | rmp_a2 | 0/0 (0) | 0/5 (0) | p>0.999 |  |
| ST152 | ybt only | 0/0 (0) | 5/5 (100) | p>0.999 |  |
| ST152 | clb and not iuc | 0/0 (0) | 0/5 (0) | p>0.999 |  |
| ST152 | iuc only | 0/0 (0) | 0/5 (0) | p>0.999 |  |
| ST152 | icu and ybt w/o clb | 0/0 (0) | 0/5 (0) | p>0.999 |  |
| ST152 | ybt, clb and iuc | 0/0 (0) | 0/5 (0) | p>0.999 |  |
| ST1552 | ybt | 0/0 (0) | 0/2 (0) | p>0.999 |  |
| ST1552 | clb | 0/0 (0) | 0/2 (0) | p>0.999 |  |
| ST1552 | iuc | 0/0 (0) | 0/2 (0) | p>0.999 |  |
| ST1552 | iro | 0/0 (0) | 0/2 (0) | p>0.999 |  |
| ST1552 | rmp_adc | 0/0 (0) | 0/2 (0) | p>0.999 |  |
| ST1552 | rmp_a2 | 0/0 (0) | 0/2 (0) | p>0.999 |  |
| ST1552 | ybt only | 0/0 (0) | 0/2 (0) | p>0.999 |  |
| ST1552 | clb and not iuc | 0/0 (0) | 0/2 (0) | p>0.999 |  |
| ST1552 | iuc only | 0/0 (0) | 0/2 (0) | p>0.999 |  |
| ST1552 | icu and ybt w/o clb | 0/0 (0) | 0/2 (0) | p>0.999 |  |
| ST1552 | ybt, clb and iuc | 0/0 (0) | 0/2 (0) | p>0.999 |  |
| ST163 | ybt | 0/1 (0) | 0/0 (0) | p>0.999 |  |
| ST163 | clb | 0/1 (0) | 0/0 (0) | p>0.999 |  |
| ST163 | iuc | 0/1 (0) | 0/0 (0) | p>0.999 |  |
| ST163 | iro | 0/1 (0) | 0/0 (0) | p>0.999 |  |
| ST163 | rmp_adc | 0/1 (0) | 0/0 (0) | p>0.999 |  |
| ST163 | rmp_a2 | 0/1 (0) | 0/0 (0) | p>0.999 |  |
| ST163 | ybt only | 0/1 (0) | 0/0 (0) | p>0.999 |  |
| ST163 | clb and not iuc | 0/1 (0) | 0/0 (0) | p>0.999 |  |
| ST163 | iuc only | 0/1 (0) | 0/0 (0) | p>0.999 |  |
| ST163 | icu and ybt w/o clb | 0/1 (0) | 0/0 (0) | p>0.999 |  |
| ST163 | ybt, clb and iuc | 0/1 (0) | 0/0 (0) | p>0.999 |  |
| ST1694 | ybt | 0/1 (0) | 0/0 (0) | p>0.999 |  |
| ST1694 | clb | 0/1 (0) | 0/0 (0) | p>0.999 |  |
| ST1694 | iuc | 0/1 (0) | 0/0 (0) | p>0.999 |  |
| ST1694 | iro | 0/1 (0) | 0/0 (0) | p>0.999 |  |
| ST1694 | rmp_adc | 0/1 (0) | 0/0 (0) | p>0.999 |  |
| ST1694 | rmp_a2 | 0/1 (0) | 0/0 (0) | p>0.999 |  |
| ST1694 | ybt only | 0/1 (0) | 0/0 (0) | p>0.999 |  |
| ST1694 | clb and not iuc | 0/1 (0) | 0/0 (0) | p>0.999 |  |
| ST1694 | iuc only | 0/1 (0) | 0/0 (0) | p>0.999 |  |
| ST1694 | icu and ybt w/o clb | 0/1 (0) | 0/0 (0) | p>0.999 |  |
| ST1694 | ybt, clb and iuc | 0/1 (0) | 0/0 (0) | p>0.999 |  |
| ST17 | ybt | 17/21 (80.952) | 12/19 (63.158) | p=0.293 |  |
| ST17 | clb | 0/21 (0) | 0/19 (0) | p>0.999 |  |
| ST17 | iuc | 0/21 (0) | 0/19 (0) | p>0.999 |  |
| ST17 | iro | 0/21 (0) | 0/19 (0) | p>0.999 |  |
| ST17 | rmp_adc | 0/21 (0) | 0/19 (0) | p>0.999 |  |
| ST17 | rmp_a2 | 0/21 (0) | 0/19 (0) | p>0.999 |  |
| ST17 | ybt only | 17/21 (80.952) | 12/19 (63.158) | p=0.293 |  |
| ST17 | clb and not iuc | 0/21 (0) | 0/19 (0) | p>0.999 |  |
| ST17 | iuc only | 0/21 (0) | 0/19 (0) | p>0.999 |  |
| ST17 | icu and ybt w/o clb | 0/21 (0) | 0/19 (0) | p>0.999 |  |
| ST17 | ybt, clb and iuc | 0/21 (0) | 0/19 (0) | p>0.999 |  |
| ST1873 | ybt | 0/0 (0) | 0/2 (0) | p>0.999 |  |
| ST1873 | clb | 0/0 (0) | 0/2 (0) | p>0.999 |  |
| ST1873 | iuc | 0/0 (0) | 0/2 (0) | p>0.999 |  |
| ST1873 | iro | 0/0 (0) | 0/2 (0) | p>0.999 |  |
| ST1873 | rmp_adc | 0/0 (0) | 0/2 (0) | p>0.999 |  |
| ST1873 | rmp_a2 | 0/0 (0) | 0/2 (0) | p>0.999 |  |
| ST1873 | ybt only | 0/0 (0) | 0/2 (0) | p>0.999 |  |
| ST1873 | clb and not iuc | 0/0 (0) | 0/2 (0) | p>0.999 |  |
| ST1873 | iuc only | 0/0 (0) | 0/2 (0) | p>0.999 |  |
| ST1873 | icu and ybt w/o clb | 0/0 (0) | 0/2 (0) | p>0.999 |  |
| ST1873 | ybt, clb and iuc | 0/0 (0) | 0/2 (0) | p>0.999 |  |
| ST193 | ybt | 0/0 (0) | 0/1 (0) | p>0.999 |  |
| ST193 | clb | 0/0 (0) | 0/1 (0) | p>0.999 |  |
| ST193 | iuc | 0/0 (0) | 0/1 (0) | p>0.999 |  |
| ST193 | iro | 0/0 (0) | 0/1 (0) | p>0.999 |  |
| ST193 | rmp_adc | 0/0 (0) | 0/1 (0) | p>0.999 |  |
| ST193 | rmp_a2 | 0/0 (0) | 0/1 (0) | p>0.999 |  |
| ST193 | ybt only | 0/0 (0) | 0/1 (0) | p>0.999 |  |
| ST193 | clb and not iuc | 0/0 (0) | 0/1 (0) | p>0.999 |  |
| ST193 | iuc only | 0/0 (0) | 0/1 (0) | p>0.999 |  |
| ST193 | icu and ybt w/o clb | 0/0 (0) | 0/1 (0) | p>0.999 |  |
| ST193 | ybt, clb and iuc | 0/0 (0) | 0/1 (0) | p>0.999 |  |
| ST1999 | ybt | 1/1 (100) | 0/0 (0) | p>0.999 |  |
| ST1999 | clb | 0/1 (0) | 0/0 (0) | p>0.999 |  |
| ST1999 | iuc | 0/1 (0) | 0/0 (0) | p>0.999 |  |
| ST1999 | iro | 0/1 (0) | 0/0 (0) | p>0.999 |  |
| ST1999 | rmp_adc | 0/1 (0) | 0/0 (0) | p>0.999 |  |
| ST1999 | rmp_a2 | 0/1 (0) | 0/0 (0) | p>0.999 |  |
| ST1999 | ybt only | 1/1 (100) | 0/0 (0) | p>0.999 |  |
| ST1999 | clb and not iuc | 0/1 (0) | 0/0 (0) | p>0.999 |  |
| ST1999 | iuc only | 0/1 (0) | 0/0 (0) | p>0.999 |  |
| ST1999 | icu and ybt w/o clb | 0/1 (0) | 0/0 (0) | p>0.999 |  |
| ST1999 | ybt, clb and iuc | 0/1 (0) | 0/0 (0) | p>0.999 |  |
| ST20 | ybt | 0/1 (0) | 0/0 (0) | p>0.999 |  |
| ST20 | clb | 0/1 (0) | 0/0 (0) | p>0.999 |  |
| ST20 | iuc | 0/1 (0) | 0/0 (0) | p>0.999 |  |
| ST20 | iro | 0/1 (0) | 0/0 (0) | p>0.999 |  |
| ST20 | rmp_adc | 0/1 (0) | 0/0 (0) | p>0.999 |  |
| ST20 | rmp_a2 | 0/1 (0) | 0/0 (0) | p>0.999 |  |
| ST20 | ybt only | 0/1 (0) | 0/0 (0) | p>0.999 |  |
| ST20 | clb and not iuc | 0/1 (0) | 0/0 (0) | p>0.999 |  |
| ST20 | iuc only | 0/1 (0) | 0/0 (0) | p>0.999 |  |
| ST20 | icu and ybt w/o clb | 0/1 (0) | 0/0 (0) | p>0.999 |  |
| ST20 | ybt, clb and iuc | 0/1 (0) | 0/0 (0) | p>0.999 |  |
| ST2039 | ybt | 0/0 (0) | 1/1 (100) | p>0.999 |  |
| ST2039 | clb | 0/0 (0) | 1/1 (100) | p>0.999 |  |
| ST2039 | iuc | 0/0 (0) | 1/1 (100) | p>0.999 |  |
| ST2039 | iro | 0/0 (0) | 1/1 (100) | p>0.999 |  |
| ST2039 | rmp_adc | 0/0 (0) | 1/1 (100) | p>0.999 |  |
| ST2039 | rmp_a2 | 0/0 (0) | 1/1 (100) | p>0.999 |  |
| ST2039 | ybt only | 0/0 (0) | 0/1 (0) | p>0.999 |  |
| ST2039 | clb and not iuc | 0/0 (0) | 0/1 (0) | p>0.999 |  |
| ST2039 | iuc only | 0/0 (0) | 0/1 (0) | p>0.999 |  |
| ST2039 | icu and ybt w/o clb | 0/0 (0) | 0/1 (0) | p>0.999 |  |
| ST2039 | ybt, clb and iuc | 0/0 (0) | 1/1 (100) | p>0.999 |  |
| ST22 | ybt | 0/2 (0) | 0/0 (0) | p>0.999 |  |
| ST22 | clb | 0/2 (0) | 0/0 (0) | p>0.999 |  |
| ST22 | iuc | 0/2 (0) | 0/0 (0) | p>0.999 |  |
| ST22 | iro | 0/2 (0) | 0/0 (0) | p>0.999 |  |
| ST22 | rmp_adc | 0/2 (0) | 0/0 (0) | p>0.999 |  |
| ST22 | rmp_a2 | 0/2 (0) | 0/0 (0) | p>0.999 |  |
| ST22 | ybt only | 0/2 (0) | 0/0 (0) | p>0.999 |  |
| ST22 | clb and not iuc | 0/2 (0) | 0/0 (0) | p>0.999 |  |
| ST22 | iuc only | 0/2 (0) | 0/0 (0) | p>0.999 |  |
| ST22 | icu and ybt w/o clb | 0/2 (0) | 0/0 (0) | p>0.999 |  |
| ST22 | ybt, clb and iuc | 0/2 (0) | 0/0 (0) | p>0.999 |  |
| ST231 | ybt | 0/0 (0) | 0/1 (0) | p>0.999 |  |
| ST231 | clb | 0/0 (0) | 0/1 (0) | p>0.999 |  |
| ST231 | iuc | 0/0 (0) | 0/1 (0) | p>0.999 |  |
| ST231 | iro | 0/0 (0) | 0/1 (0) | p>0.999 |  |
| ST231 | rmp_adc | 0/0 (0) | 0/1 (0) | p>0.999 |  |
| ST231 | rmp_a2 | 0/0 (0) | 0/1 (0) | p>0.999 |  |
| ST231 | ybt only | 0/0 (0) | 0/1 (0) | p>0.999 |  |
| ST231 | clb and not iuc | 0/0 (0) | 0/1 (0) | p>0.999 |  |
| ST231 | iuc only | 0/0 (0) | 0/1 (0) | p>0.999 |  |
| ST231 | icu and ybt w/o clb | 0/0 (0) | 0/1 (0) | p>0.999 |  |
| ST231 | ybt, clb and iuc | 0/0 (0) | 0/1 (0) | p>0.999 |  |
| ST2441 | ybt | 0/0 (0) | 0/1 (0) | p>0.999 |  |
| ST2441 | clb | 0/0 (0) | 0/1 (0) | p>0.999 |  |
| ST2441 | iuc | 0/0 (0) | 0/1 (0) | p>0.999 |  |
| ST2441 | iro | 0/0 (0) | 0/1 (0) | p>0.999 |  |
| ST2441 | rmp_adc | 0/0 (0) | 0/1 (0) | p>0.999 |  |
| ST2441 | rmp_a2 | 0/0 (0) | 0/1 (0) | p>0.999 |  |
| ST2441 | ybt only | 0/0 (0) | 0/1 (0) | p>0.999 |  |
| ST2441 | clb and not iuc | 0/0 (0) | 0/1 (0) | p>0.999 |  |
| ST2441 | iuc only | 0/0 (0) | 0/1 (0) | p>0.999 |  |
| ST2441 | icu and ybt w/o clb | 0/0 (0) | 0/1 (0) | p>0.999 |  |
| ST2441 | ybt, clb and iuc | 0/0 (0) | 0/1 (0) | p>0.999 |  |
| ST25 | ybt | 0/0 (0) | 1/2 (50) | p>0.999 |  |
| ST25 | clb | 0/0 (0) | 0/2 (0) | p>0.999 |  |
| ST25 | iuc | 0/0 (0) | 0/2 (0) | p>0.999 |  |
| ST25 | iro | 0/0 (0) | 0/2 (0) | p>0.999 |  |
| ST25 | rmp_adc | 0/0 (0) | 0/2 (0) | p>0.999 |  |
| ST25 | rmp_a2 | 0/0 (0) | 0/2 (0) | p>0.999 |  |
| ST25 | ybt only | 0/0 (0) | 1/2 (50) | p>0.999 |  |
| ST25 | clb and not iuc | 0/0 (0) | 0/2 (0) | p>0.999 |  |
| ST25 | iuc only | 0/0 (0) | 0/2 (0) | p>0.999 |  |
| ST25 | icu and ybt w/o clb | 0/0 (0) | 0/2 (0) | p>0.999 |  |
| ST25 | ybt, clb and iuc | 0/0 (0) | 0/2 (0) | p>0.999 |  |
| ST252 | ybt | 0/1 (0) | 1/1 (100) | p>0.999 |  |
| ST252 | clb | 0/1 (0) | 0/1 (0) | p>0.999 |  |
| ST252 | iuc | 0/1 (0) | 0/1 (0) | p>0.999 |  |
| ST252 | iro | 0/1 (0) | 0/1 (0) | p>0.999 |  |
| ST252 | rmp_adc | 0/1 (0) | 0/1 (0) | p>0.999 |  |
| ST252 | rmp_a2 | 0/1 (0) | 0/1 (0) | p>0.999 |  |
| ST252 | ybt only | 0/1 (0) | 1/1 (100) | p>0.999 |  |
| ST252 | clb and not iuc | 0/1 (0) | 0/1 (0) | p>0.999 |  |
| ST252 | iuc only | 0/1 (0) | 0/1 (0) | p>0.999 |  |
| ST252 | icu and ybt w/o clb | 0/1 (0) | 0/1 (0) | p>0.999 |  |
| ST252 | ybt, clb and iuc | 0/1 (0) | 0/1 (0) | p>0.999 |  |
| ST307 | ybt | 0/8 (0) | 0/17 (0) | p>0.999 |  |
| ST307 | clb | 0/8 (0) | 0/17 (0) | p>0.999 |  |
| ST307 | iuc | 0/8 (0) | 0/17 (0) | p>0.999 |  |
| ST307 | iro | 0/8 (0) | 0/17 (0) | p>0.999 |  |
| ST307 | rmp_adc | 0/8 (0) | 0/17 (0) | p>0.999 |  |
| ST307 | rmp_a2 | 0/8 (0) | 0/17 (0) | p>0.999 |  |
| ST307 | ybt only | 0/8 (0) | 0/17 (0) | p>0.999 |  |
| ST307 | clb and not iuc | 0/8 (0) | 0/17 (0) | p>0.999 |  |
| ST307 | iuc only | 0/8 (0) | 0/17 (0) | p>0.999 |  |
| ST307 | icu and ybt w/o clb | 0/8 (0) | 0/17 (0) | p>0.999 |  |
| ST307 | ybt, clb and iuc | 0/8 (0) | 0/17 (0) | p>0.999 |  |
| ST309 | ybt | 0/1 (0) | 0/0 (0) | p>0.999 |  |
| ST309 | clb | 0/1 (0) | 0/0 (0) | p>0.999 |  |
| ST309 | iuc | 0/1 (0) | 0/0 (0) | p>0.999 |  |
| ST309 | iro | 0/1 (0) | 0/0 (0) | p>0.999 |  |
| ST309 | rmp_adc | 0/1 (0) | 0/0 (0) | p>0.999 |  |
| ST309 | rmp_a2 | 0/1 (0) | 0/0 (0) | p>0.999 |  |
| ST309 | ybt only | 0/1 (0) | 0/0 (0) | p>0.999 |  |
| ST309 | clb and not iuc | 0/1 (0) | 0/0 (0) | p>0.999 |  |
| ST309 | iuc only | 0/1 (0) | 0/0 (0) | p>0.999 |  |
| ST309 | icu and ybt w/o clb | 0/1 (0) | 0/0 (0) | p>0.999 |  |
| ST309 | ybt, clb and iuc | 0/1 (0) | 0/0 (0) | p>0.999 |  |
| ST336 | ybt | 0/0 (0) | 1/1 (100) | p>0.999 |  |
| ST336 | clb | 0/0 (0) | 0/1 (0) | p>0.999 |  |
| ST336 | iuc | 0/0 (0) | 0/1 (0) | p>0.999 |  |
| ST336 | iro | 0/0 (0) | 0/1 (0) | p>0.999 |  |
| ST336 | rmp_adc | 0/0 (0) | 0/1 (0) | p>0.999 |  |
| ST336 | rmp_a2 | 0/0 (0) | 0/1 (0) | p>0.999 |  |
| ST336 | ybt only | 0/0 (0) | 1/1 (100) | p>0.999 |  |
| ST336 | clb and not iuc | 0/0 (0) | 0/1 (0) | p>0.999 |  |
| ST336 | iuc only | 0/0 (0) | 0/1 (0) | p>0.999 |  |
| ST336 | icu and ybt w/o clb | 0/0 (0) | 0/1 (0) | p>0.999 |  |
| ST336 | ybt, clb and iuc | 0/0 (0) | 0/1 (0) | p>0.999 |  |
| ST34 | ybt | 0/1 (0) | 0/0 (0) | p>0.999 |  |
| ST34 | clb | 0/1 (0) | 0/0 (0) | p>0.999 |  |
| ST34 | iuc | 0/1 (0) | 0/0 (0) | p>0.999 |  |
| ST34 | iro | 0/1 (0) | 0/0 (0) | p>0.999 |  |
| ST34 | rmp_adc | 0/1 (0) | 0/0 (0) | p>0.999 |  |
| ST34 | rmp_a2 | 0/1 (0) | 0/0 (0) | p>0.999 |  |
| ST34 | ybt only | 0/1 (0) | 0/0 (0) | p>0.999 |  |
| ST34 | clb and not iuc | 0/1 (0) | 0/0 (0) | p>0.999 |  |
| ST34 | iuc only | 0/1 (0) | 0/0 (0) | p>0.999 |  |
| ST34 | icu and ybt w/o clb | 0/1 (0) | 0/0 (0) | p>0.999 |  |
| ST34 | ybt, clb and iuc | 0/1 (0) | 0/0 (0) | p>0.999 |  |
| ST35 | ybt | 1/1 (100) | 0/1 (0) | p>0.999 |  |
| ST35 | clb | 0/1 (0) | 0/1 (0) | p>0.999 |  |
| ST35 | iuc | 0/1 (0) | 0/1 (0) | p>0.999 |  |
| ST35 | iro | 0/1 (0) | 0/1 (0) | p>0.999 |  |
| ST35 | rmp_adc | 0/1 (0) | 0/1 (0) | p>0.999 |  |
| ST35 | rmp_a2 | 0/1 (0) | 0/1 (0) | p>0.999 |  |
| ST35 | ybt only | 1/1 (100) | 0/1 (0) | p>0.999 |  |
| ST35 | clb and not iuc | 0/1 (0) | 0/1 (0) | p>0.999 |  |
| ST35 | iuc only | 0/1 (0) | 0/1 (0) | p>0.999 |  |
| ST35 | icu and ybt w/o clb | 0/1 (0) | 0/1 (0) | p>0.999 |  |
| ST35 | ybt, clb and iuc | 0/1 (0) | 0/1 (0) | p>0.999 |  |
| ST353 | ybt | 1/1 (100) | 4/4 (100) | p>0.999 |  |
| ST353 | clb | 0/1 (0) | 0/4 (0) | p>0.999 |  |
| ST353 | iuc | 0/1 (0) | 0/4 (0) | p>0.999 |  |
| ST353 | iro | 0/1 (0) | 0/4 (0) | p>0.999 |  |
| ST353 | rmp_adc | 0/1 (0) | 0/4 (0) | p>0.999 |  |
| ST353 | rmp_a2 | 0/1 (0) | 0/4 (0) | p>0.999 |  |
| ST353 | ybt only | 1/1 (100) | 4/4 (100) | p>0.999 |  |
| ST353 | clb and not iuc | 0/1 (0) | 0/4 (0) | p>0.999 |  |
| ST353 | iuc only | 0/1 (0) | 0/4 (0) | p>0.999 |  |
| ST353 | icu and ybt w/o clb | 0/1 (0) | 0/4 (0) | p>0.999 |  |
| ST353 | ybt, clb and iuc | 0/1 (0) | 0/4 (0) | p>0.999 |  |
| ST3688 | ybt | 0/1 (0) | 0/0 (0) | p>0.999 |  |
| ST3688 | clb | 0/1 (0) | 0/0 (0) | p>0.999 |  |
| ST3688 | iuc | 0/1 (0) | 0/0 (0) | p>0.999 |  |
| ST3688 | iro | 0/1 (0) | 0/0 (0) | p>0.999 |  |
| ST3688 | rmp_adc | 0/1 (0) | 0/0 (0) | p>0.999 |  |
| ST3688 | rmp_a2 | 0/1 (0) | 0/0 (0) | p>0.999 |  |
| ST3688 | ybt only | 0/1 (0) | 0/0 (0) | p>0.999 |  |
| ST3688 | clb and not iuc | 0/1 (0) | 0/0 (0) | p>0.999 |  |
| ST3688 | iuc only | 0/1 (0) | 0/0 (0) | p>0.999 |  |
| ST3688 | icu and ybt w/o clb | 0/1 (0) | 0/0 (0) | p>0.999 |  |
| ST3688 | ybt, clb and iuc | 0/1 (0) | 0/0 (0) | p>0.999 |  |
| ST37 | ybt | 0/1 (0) | 0/0 (0) | p>0.999 |  |
| ST37 | clb | 0/1 (0) | 0/0 (0) | p>0.999 |  |
| ST37 | iuc | 0/1 (0) | 0/0 (0) | p>0.999 |  |
| ST37 | iro | 0/1 (0) | 0/0 (0) | p>0.999 |  |
| ST37 | rmp_adc | 0/1 (0) | 0/0 (0) | p>0.999 |  |
| ST37 | rmp_a2 | 0/1 (0) | 0/0 (0) | p>0.999 |  |
| ST37 | ybt only | 0/1 (0) | 0/0 (0) | p>0.999 |  |
| ST37 | clb and not iuc | 0/1 (0) | 0/0 (0) | p>0.999 |  |
| ST37 | iuc only | 0/1 (0) | 0/0 (0) | p>0.999 |  |
| ST37 | icu and ybt w/o clb | 0/1 (0) | 0/0 (0) | p>0.999 |  |
| ST37 | ybt, clb and iuc | 0/1 (0) | 0/0 (0) | p>0.999 |  |
| ST39 | ybt | 0/0 (0) | 23/23 (100) | p>0.999 |  |
| ST39 | clb | 0/0 (0) | 0/23 (0) | p>0.999 |  |
| ST39 | iuc | 0/0 (0) | 0/23 (0) | p>0.999 |  |
| ST39 | iro | 0/0 (0) | 0/23 (0) | p>0.999 |  |
| ST39 | rmp_adc | 0/0 (0) | 0/23 (0) | p>0.999 |  |
| ST39 | rmp_a2 | 0/0 (0) | 0/23 (0) | p>0.999 |  |
| ST39 | ybt only | 0/0 (0) | 23/23 (100) | p>0.999 |  |
| ST39 | clb and not iuc | 0/0 (0) | 0/23 (0) | p>0.999 |  |
| ST39 | iuc only | 0/0 (0) | 0/23 (0) | p>0.999 |  |
| ST39 | icu and ybt w/o clb | 0/0 (0) | 0/23 (0) | p>0.999 |  |
| ST39 | ybt, clb and iuc | 0/0 (0) | 0/23 (0) | p>0.999 |  |
| ST3985 | ybt | 0/1 (0) | 0/0 (0) | p>0.999 |  |
| ST3985 | clb | 0/1 (0) | 0/0 (0) | p>0.999 |  |
| ST3985 | iuc | 0/1 (0) | 0/0 (0) | p>0.999 |  |
| ST3985 | iro | 1/1 (100) | 0/0 (0) | p>0.999 |  |
| ST3985 | rmp_adc | 0/1 (0) | 0/0 (0) | p>0.999 |  |
| ST3985 | rmp_a2 | 1/1 (100) | 0/0 (0) | p>0.999 |  |
| ST3985 | ybt only | 0/1 (0) | 0/0 (0) | p>0.999 |  |
| ST3985 | clb and not iuc | 0/1 (0) | 0/0 (0) | p>0.999 |  |
| ST3985 | iuc only | 0/1 (0) | 0/0 (0) | p>0.999 |  |
| ST3985 | icu and ybt w/o clb | 0/1 (0) | 0/0 (0) | p>0.999 |  |
| ST3985 | ybt, clb and iuc | 0/1 (0) | 0/0 (0) | p>0.999 |  |
| ST416 | ybt | 0/1 (0) | 0/0 (0) | p>0.999 |  |
| ST416 | clb | 0/1 (0) | 0/0 (0) | p>0.999 |  |
| ST416 | iuc | 0/1 (0) | 0/0 (0) | p>0.999 |  |
| ST416 | iro | 0/1 (0) | 0/0 (0) | p>0.999 |  |
| ST416 | rmp_adc | 0/1 (0) | 0/0 (0) | p>0.999 |  |
| ST416 | rmp_a2 | 0/1 (0) | 0/0 (0) | p>0.999 |  |
| ST416 | ybt only | 0/1 (0) | 0/0 (0) | p>0.999 |  |
| ST416 | clb and not iuc | 0/1 (0) | 0/0 (0) | p>0.999 |  |
| ST416 | iuc only | 0/1 (0) | 0/0 (0) | p>0.999 |  |
| ST416 | icu and ybt w/o clb | 0/1 (0) | 0/0 (0) | p>0.999 |  |
| ST416 | ybt, clb and iuc | 0/1 (0) | 0/0 (0) | p>0.999 |  |
| ST4291 | ybt | 0/0 (0) | 0/2 (0) | p>0.999 |  |
| ST4291 | clb | 0/0 (0) | 0/2 (0) | p>0.999 |  |
| ST4291 | iuc | 0/0 (0) | 0/2 (0) | p>0.999 |  |
| ST4291 | iro | 0/0 (0) | 0/2 (0) | p>0.999 |  |
| ST4291 | rmp_adc | 0/0 (0) | 0/2 (0) | p>0.999 |  |
| ST4291 | rmp_a2 | 0/0 (0) | 0/2 (0) | p>0.999 |  |
| ST4291 | ybt only | 0/0 (0) | 0/2 (0) | p>0.999 |  |
| ST4291 | clb and not iuc | 0/0 (0) | 0/2 (0) | p>0.999 |  |
| ST4291 | iuc only | 0/0 (0) | 0/2 (0) | p>0.999 |  |
| ST4291 | icu and ybt w/o clb | 0/0 (0) | 0/2 (0) | p>0.999 |  |
| ST4291 | ybt, clb and iuc | 0/0 (0) | 0/2 (0) | p>0.999 |  |
| ST460 | ybt | 0/1 (0) | 0/0 (0) | p>0.999 |  |
| ST460 | clb | 0/1 (0) | 0/0 (0) | p>0.999 |  |
| ST460 | iuc | 0/1 (0) | 0/0 (0) | p>0.999 |  |
| ST460 | iro | 0/1 (0) | 0/0 (0) | p>0.999 |  |
| ST460 | rmp_adc | 0/1 (0) | 0/0 (0) | p>0.999 |  |
| ST460 | rmp_a2 | 0/1 (0) | 0/0 (0) | p>0.999 |  |
| ST460 | ybt only | 0/1 (0) | 0/0 (0) | p>0.999 |  |
| ST460 | clb and not iuc | 0/1 (0) | 0/0 (0) | p>0.999 |  |
| ST460 | iuc only | 0/1 (0) | 0/0 (0) | p>0.999 |  |
| ST460 | icu and ybt w/o clb | 0/1 (0) | 0/0 (0) | p>0.999 |  |
| ST460 | ybt, clb and iuc | 0/1 (0) | 0/0 (0) | p>0.999 |  |
| ST461 | ybt | 0/1 (0) | 0/0 (0) | p>0.999 |  |
| ST461 | clb | 0/1 (0) | 0/0 (0) | p>0.999 |  |
| ST461 | iuc | 0/1 (0) | 0/0 (0) | p>0.999 |  |
| ST461 | iro | 0/1 (0) | 0/0 (0) | p>0.999 |  |
| ST461 | rmp_adc | 0/1 (0) | 0/0 (0) | p>0.999 |  |
| ST461 | rmp_a2 | 0/1 (0) | 0/0 (0) | p>0.999 |  |
| ST461 | ybt only | 0/1 (0) | 0/0 (0) | p>0.999 |  |
| ST461 | clb and not iuc | 0/1 (0) | 0/0 (0) | p>0.999 |  |
| ST461 | iuc only | 0/1 (0) | 0/0 (0) | p>0.999 |  |
| ST461 | icu and ybt w/o clb | 0/1 (0) | 0/0 (0) | p>0.999 |  |
| ST461 | ybt, clb and iuc | 0/1 (0) | 0/0 (0) | p>0.999 |  |
| ST502 | ybt | 0/4 (0) | 0/0 (0) | p>0.999 |  |
| ST502 | clb | 0/4 (0) | 0/0 (0) | p>0.999 |  |
| ST502 | iuc | 0/4 (0) | 0/0 (0) | p>0.999 |  |
| ST502 | iro | 0/4 (0) | 0/0 (0) | p>0.999 |  |
| ST502 | rmp_adc | 0/4 (0) | 0/0 (0) | p>0.999 |  |
| ST502 | rmp_a2 | 0/4 (0) | 0/0 (0) | p>0.999 |  |
| ST502 | ybt only | 0/4 (0) | 0/0 (0) | p>0.999 |  |
| ST502 | clb and not iuc | 0/4 (0) | 0/0 (0) | p>0.999 |  |
| ST502 | iuc only | 0/4 (0) | 0/0 (0) | p>0.999 |  |
| ST502 | icu and ybt w/o clb | 0/4 (0) | 0/0 (0) | p>0.999 |  |
| ST502 | ybt, clb and iuc | 0/4 (0) | 0/0 (0) | p>0.999 |  |
| ST607 | ybt | 0/0 (0) | 1/2 (50) | p>0.999 |  |
| ST607 | clb | 0/0 (0) | 0/2 (0) | p>0.999 |  |
| ST607 | iuc | 0/0 (0) | 0/2 (0) | p>0.999 |  |
| ST607 | iro | 0/0 (0) | 0/2 (0) | p>0.999 |  |
| ST607 | rmp_adc | 0/0 (0) | 0/2 (0) | p>0.999 |  |
| ST607 | rmp_a2 | 0/0 (0) | 0/2 (0) | p>0.999 |  |
| ST607 | ybt only | 0/0 (0) | 1/2 (50) | p>0.999 |  |
| ST607 | clb and not iuc | 0/0 (0) | 0/2 (0) | p>0.999 |  |
| ST607 | iuc only | 0/0 (0) | 0/2 (0) | p>0.999 |  |
| ST607 | icu and ybt w/o clb | 0/0 (0) | 0/2 (0) | p>0.999 |  |
| ST607 | ybt, clb and iuc | 0/0 (0) | 0/2 (0) | p>0.999 |  |
| ST611 | ybt | 0/1 (0) | 0/0 (0) | p>0.999 |  |
| ST611 | clb | 0/1 (0) | 0/0 (0) | p>0.999 |  |
| ST611 | iuc | 0/1 (0) | 0/0 (0) | p>0.999 |  |
| ST611 | iro | 0/1 (0) | 0/0 (0) | p>0.999 |  |
| ST611 | rmp_adc | 0/1 (0) | 0/0 (0) | p>0.999 |  |
| ST611 | rmp_a2 | 0/1 (0) | 0/0 (0) | p>0.999 |  |
| ST611 | ybt only | 0/1 (0) | 0/0 (0) | p>0.999 |  |
| ST611 | clb and not iuc | 0/1 (0) | 0/0 (0) | p>0.999 |  |
| ST611 | iuc only | 0/1 (0) | 0/0 (0) | p>0.999 |  |
| ST611 | icu and ybt w/o clb | 0/1 (0) | 0/0 (0) | p>0.999 |  |
| ST611 | ybt, clb and iuc | 0/1 (0) | 0/0 (0) | p>0.999 |  |
| ST987 | ybt | 0/0 (0) | 0/1 (0) | p>0.999 |  |
| ST987 | clb | 0/0 (0) | 0/1 (0) | p>0.999 |  |
| ST987 | iuc | 0/0 (0) | 0/1 (0) | p>0.999 |  |
| ST987 | iro | 0/0 (0) | 0/1 (0) | p>0.999 |  |
| ST987 | rmp_adc | 0/0 (0) | 0/1 (0) | p>0.999 |  |
| ST987 | rmp_a2 | 0/0 (0) | 0/1 (0) | p>0.999 |  |
| ST987 | ybt only | 0/0 (0) | 0/1 (0) | p>0.999 |  |
| ST987 | clb and not iuc | 0/0 (0) | 0/1 (0) | p>0.999 |  |
| ST987 | iuc only | 0/0 (0) | 0/1 (0) | p>0.999 |  |
| ST987 | icu and ybt w/o clb | 0/0 (0) | 0/1 (0) | p>0.999 |  |
| ST987 | ybt, clb and iuc | 0/0 (0) | 0/1 (0) | p>0.999 |  |
| **O- loci, n(%)** |  |  |  |  |  |
| O1/O2v1 | ybt | 8/42 (19.048) | 10/16 (62.5) | p=0.003 |  |
| O1/O2v1 | clb | 0/42 (0) | 1/16 (6.25) | p=0.276 |  |
| O1/O2v1 | iuc | 0/42 (0) | 1/16 (6.25) | p=0.276 |  |
| O1/O2v1 | iro | 0/42 (0) | 1/16 (6.25) | p=0.276 |  |
| O1/O2v1 | rmp_adc | 0/42 (0) | 1/16 (6.25) | p=0.276 |  |
| O1/O2v1 | rmp_a2 | 0/42 (0) | 1/16 (6.25) | p=0.276 |  |
| O1/O2v1 | ybt only | 8/42 (19.048) | 9/16 (56.25) | p=0.009 |  |
| O1/O2v1 | clb and not iuc | 0/42 (0) | 0/16 (0) | p>0.999 |  |
| O1/O2v1 | iuc only | 0/42 (0) | 0/16 (0) | p>0.999 |  |
| O1/O2v1 | icu and ybt w/o clb | 0/42 (0) | 0/16 (0) | p>0.999 |  |
| O1/O2v1 | ybt, clb and iuc | 0/42 (0) | 1/16 (6.25) | p=0.276 |  |
| O1/O2v2 | ybt | 3/19 (15.789) | 29/55 (52.727) | p=0.007 |  |
| O1/O2v2 | clb | 0/19 (0) | 0/55 (0) | p>0.999 |  |
| O1/O2v2 | iuc | 0/19 (0) | 0/55 (0) | p>0.999 |  |
| O1/O2v2 | iro | 0/19 (0) | 0/55 (0) | p>0.999 |  |
| O1/O2v2 | rmp_adc | 0/19 (0) | 0/55 (0) | p>0.999 |  |
| O1/O2v2 | rmp_a2 | 0/19 (0) | 0/55 (0) | p>0.999 |  |
| O1/O2v2 | ybt only | 3/19 (15.789) | 29/55 (52.727) | p=0.007 |  |
| O1/O2v2 | clb and not iuc | 0/19 (0) | 0/55 (0) | p>0.999 |  |
| O1/O2v2 | iuc only | 0/19 (0) | 0/55 (0) | p>0.999 |  |
| O1/O2v2 | icu and ybt w/o clb | 0/19 (0) | 0/55 (0) | p>0.999 |  |
| O1/O2v2 | ybt, clb and iuc | 0/19 (0) | 0/55 (0) | p>0.999 |  |
| O12 | ybt | 1/1 (100) | 0/0 (0) | p>0.999 |  |
| O12 | clb | 0/1 (0) | 0/0 (0) | p>0.999 |  |
| O12 | iuc | 0/1 (0) | 0/0 (0) | p>0.999 |  |
| O12 | iro | 0/1 (0) | 0/0 (0) | p>0.999 |  |
| O12 | rmp_adc | 0/1 (0) | 0/0 (0) | p>0.999 |  |
| O12 | rmp_a2 | 0/1 (0) | 0/0 (0) | p>0.999 |  |
| O12 | ybt only | 1/1 (100) | 0/0 (0) | p>0.999 |  |
| O12 | clb and not iuc | 0/1 (0) | 0/0 (0) | p>0.999 |  |
| O12 | iuc only | 0/1 (0) | 0/0 (0) | p>0.999 |  |
| O12 | icu and ybt w/o clb | 0/1 (0) | 0/0 (0) | p>0.999 |  |
| O12 | ybt, clb and iuc | 0/1 (0) | 0/0 (0) | p>0.999 |  |
| O3/O3a | ybt | 0/1 (0) | 0/0 (0) | p>0.999 |  |
| O3/O3a | clb | 0/1 (0) | 0/0 (0) | p>0.999 |  |
| O3/O3a | iuc | 0/1 (0) | 0/0 (0) | p>0.999 |  |
| O3/O3a | iro | 0/1 (0) | 0/0 (0) | p>0.999 |  |
| O3/O3a | rmp_adc | 0/1 (0) | 0/0 (0) | p>0.999 |  |
| O3/O3a | rmp_a2 | 0/1 (0) | 0/0 (0) | p>0.999 |  |
| O3/O3a | ybt only | 0/1 (0) | 0/0 (0) | p>0.999 |  |
| O3/O3a | clb and not iuc | 0/1 (0) | 0/0 (0) | p>0.999 |  |
| O3/O3a | iuc only | 0/1 (0) | 0/0 (0) | p>0.999 |  |
| O3/O3a | icu and ybt w/o clb | 0/1 (0) | 0/0 (0) | p>0.999 |  |
| O3/O3a | ybt, clb and iuc | 0/1 (0) | 0/0 (0) | p>0.999 |  |
| O3b | ybt | 1/4 (25) | 4/5 (80) | p=0.206 |  |
| O3b | clb | 0/4 (0) | 0/5 (0) | p>0.999 |  |
| O3b | iuc | 0/4 (0) | 0/5 (0) | p>0.999 |  |
| O3b | iro | 0/4 (0) | 0/5 (0) | p>0.999 |  |
| O3b | rmp_adc | 0/4 (0) | 0/5 (0) | p>0.999 |  |
| O3b | rmp_a2 | 0/4 (0) | 0/5 (0) | p>0.999 |  |
| O3b | ybt only | 1/4 (25) | 4/5 (80) | p=0.206 |  |
| O3b | clb and not iuc | 0/4 (0) | 0/5 (0) | p>0.999 |  |
| O3b | iuc only | 0/4 (0) | 0/5 (0) | p>0.999 |  |
| O3b | icu and ybt w/o clb | 0/4 (0) | 0/5 (0) | p>0.999 |  |
| O3b | ybt, clb and iuc | 0/4 (0) | 0/5 (0) | p>0.999 |  |
| O4 | ybt | 0/7 (0) | 5/5 (100) | p=0.001 |  |
| O4 | clb | 0/7 (0) | 0/5 (0) | p>0.999 |  |
| O4 | iuc | 0/7 (0) | 0/5 (0) | p>0.999 |  |
| O4 | iro | 1/7 (14.286) | 0/5 (0) | p>0.999 |  |
| O4 | rmp_adc | 0/7 (0) | 0/5 (0) | p>0.999 |  |
| O4 | rmp_a2 | 1/7 (14.286) | 0/5 (0) | p>0.999 |  |
| O4 | ybt only | 0/7 (0) | 5/5 (100) | p=0.001 |  |
| O4 | clb and not iuc | 0/7 (0) | 0/5 (0) | p>0.999 |  |
| O4 | iuc only | 0/7 (0) | 0/5 (0) | p>0.999 |  |
| O4 | icu and ybt w/o clb | 0/7 (0) | 0/5 (0) | p>0.999 |  |
| O4 | ybt, clb and iuc | 0/7 (0) | 0/5 (0) | p>0.999 |  |
| O5 | ybt | 17/20 (85) | 13/17 (76.471) | p=0.68 |  |
| O5 | clb | 0/20 (0) | 0/17 (0) | p>0.999 |  |
| O5 | iuc | 0/20 (0) | 0/17 (0) | p>0.999 |  |
| O5 | iro | 0/20 (0) | 0/17 (0) | p>0.999 |  |
| O5 | rmp_adc | 0/20 (0) | 0/17 (0) | p>0.999 |  |
| O5 | rmp_a2 | 0/20 (0) | 0/17 (0) | p>0.999 |  |
| O5 | ybt only | 17/20 (85) | 13/17 (76.471) | p=0.68 |  |
| O5 | clb and not iuc | 0/20 (0) | 0/17 (0) | p>0.999 |  |
| O5 | iuc only | 0/20 (0) | 0/17 (0) | p>0.999 |  |
| O5 | icu and ybt w/o clb | 0/20 (0) | 0/17 (0) | p>0.999 |  |
| O5 | ybt, clb and iuc | 0/20 (0) | 0/17 (0) | p>0.999 |  |
| **O-antigen type, n(%)** |  |  |  |  |  |
| O1ab | ybt | 11/47 (23.404) | 37/45 (82.222) | p<0.001 |  |
| O1ab | clb | 0/47 (0) | 1/45 (2.222) | p=0.495 |  |
| O1ab | iuc | 0/47 (0) | 1/45 (2.222) | p=0.495 |  |
| O1ab | iro | 0/47 (0) | 1/45 (2.222) | p=0.495 |  |
| O1ab | rmp_adc | 0/47 (0) | 1/45 (2.222) | p=0.495 |  |
| O1ab | rmp_a2 | 0/47 (0) | 1/45 (2.222) | p=0.495 |  |
| O1ab | ybt only | 11/47 (23.404) | 37/45 (82.222) | p<0.001 |  |
| O1ab | clb and not iuc | 0/47 (0) | 0/45 (0) | p>0.999 |  |
| O1ab | iuc only | 0/47 (0) | 0/45 (0) | p>0.999 |  |
| O1ab | icu and ybt w/o clb | 0/47 (0) | 0/45 (0) | p>0.999 |  |
| O1ab | ybt, clb and iuc | 0/47 (0) | 1/45 (2.222) | p=0.495 |  |
| O12 | ybt | 1/1 (100) | 0/0 (0) | p>0.999 |  |
| O12 | clb | 0/1 (0) | 0/0 (0) | p>0.999 |  |
| O12 | iuc | 0/1 (0) | 0/0 (0) | p>0.999 |  |
| O12 | iro | 0/1 (0) | 0/0 (0) | p>0.999 |  |
| O12 | rmp_adc | 0/1 (0) | 0/0 (0) | p>0.999 |  |
| O12 | rmp_a2 | 0/1 (0) | 0/0 (0) | p>0.999 |  |
| O12 | ybt only | 1/1 (100) | 0/0 (0) | p>0.999 |  |
| O12 | clb and not iuc | 0/1 (0) | 0/0 (0) | p>0.999 |  |
| O12 | iuc only | 0/1 (0) | 0/0 (0) | p>0.999 |  |
| O12 | icu and ybt w/o clb | 0/1 (0) | 0/0 (0) | p>0.999 |  |
| O12 | ybt, clb and iuc | 0/1 (0) | 0/0 (0) | p>0.999 |  |
| O2a | ybt | 0/3 (0) | 0/2 (0) | p>0.999 |  |
| O2a | clb | 0/3 (0) | 0/2 (0) | p>0.999 |  |
| O2a | iuc | 0/3 (0) | 0/2 (0) | p>0.999 |  |
| O2a | iro | 0/3 (0) | 0/2 (0) | p>0.999 |  |
| O2a | rmp_adc | 0/3 (0) | 0/2 (0) | p>0.999 |  |
| O2a | rmp_a2 | 0/3 (0) | 0/2 (0) | p>0.999 |  |
| O2a | ybt only | 0/3 (0) | 0/2 (0) | p>0.999 |  |
| O2a | clb and not iuc | 0/3 (0) | 0/2 (0) | p>0.999 |  |
| O2a | iuc only | 0/3 (0) | 0/2 (0) | p>0.999 |  |
| O2a | icu and ybt w/o clb | 0/3 (0) | 0/2 (0) | p>0.999 |  |
| O2a | ybt, clb and iuc | 0/3 (0) | 0/2 (0) | p>0.999 |  |
| O2afg | ybt | 0/11 (0) | 1/24 (4.167) | p>0.999 |  |
| O2afg | clb | 0/11 (0) | 0/24 (0) | p>0.999 |  |
| O2afg | iuc | 0/11 (0) | 0/24 (0) | p>0.999 |  |
| O2afg | iro | 0/11 (0) | 0/24 (0) | p>0.999 |  |
| O2afg | rmp_adc | 0/11 (0) | 0/24 (0) | p>0.999 |  |
| O2afg | rmp_a2 | 0/11 (0) | 0/24 (0) | p>0.999 |  |
| O2afg | ybt only | 0/11 (0) | 1/24 (4.167) | p>0.999 |  |
| O2afg | clb and not iuc | 0/11 (0) | 0/24 (0) | p>0.999 |  |
| O2afg | iuc only | 0/11 (0) | 0/24 (0) | p>0.999 |  |
| O2afg | icu and ybt w/o clb | 0/11 (0) | 0/24 (0) | p>0.999 |  |
| O2afg | ybt, clb and iuc | 0/11 (0) | 0/24 (0) | p>0.999 |  |
| O3/O3a | ybt | 0/1 (0) | 0/0 (0) | p>0.999 |  |
| O3/O3a | clb | 0/1 (0) | 0/0 (0) | p>0.999 |  |
| O3/O3a | iuc | 0/1 (0) | 0/0 (0) | p>0.999 |  |
| O3/O3a | iro | 0/1 (0) | 0/0 (0) | p>0.999 |  |
| O3/O3a | rmp_adc | 0/1 (0) | 0/0 (0) | p>0.999 |  |
| O3/O3a | rmp_a2 | 0/1 (0) | 0/0 (0) | p>0.999 |  |
| O3/O3a | ybt only | 0/1 (0) | 0/0 (0) | p>0.999 |  |
| O3/O3a | clb and not iuc | 0/1 (0) | 0/0 (0) | p>0.999 |  |
| O3/O3a | iuc only | 0/1 (0) | 0/0 (0) | p>0.999 |  |
| O3/O3a | icu and ybt w/o clb | 0/1 (0) | 0/0 (0) | p>0.999 |  |
| O3/O3a | ybt, clb and iuc | 0/1 (0) | 0/0 (0) | p>0.999 |  |
| O3b | ybt | 1/4 (25) | 4/5 (80) | p=0.206 |  |
| O3b | clb | 0/4 (0) | 0/5 (0) | p>0.999 |  |
| O3b | iuc | 0/4 (0) | 0/5 (0) | p>0.999 |  |
| O3b | iro | 0/4 (0) | 0/5 (0) | p>0.999 |  |
| O3b | rmp_adc | 0/4 (0) | 0/5 (0) | p>0.999 |  |
| O3b | rmp_a2 | 0/4 (0) | 0/5 (0) | p>0.999 |  |
| O3b | ybt only | 1/4 (25) | 4/5 (80) | p=0.206 |  |
| O3b | clb and not iuc | 0/4 (0) | 0/5 (0) | p>0.999 |  |
| O3b | iuc only | 0/4 (0) | 0/5 (0) | p>0.999 |  |
| O3b | icu and ybt w/o clb | 0/4 (0) | 0/5 (0) | p>0.999 |  |
| O3b | ybt, clb and iuc | 0/4 (0) | 0/5 (0) | p>0.999 |  |
| O4 | ybt | 0/7 (0) | 5/5 (100) | p=0.001 |  |
| O4 | clb | 0/7 (0) | 0/5 (0) | p>0.999 |  |
| O4 | iuc | 0/7 (0) | 0/5 (0) | p>0.999 |  |
| O4 | iro | 1/7 (14.286) | 0/5 (0) | p>0.999 |  |
| O4 | rmp_adc | 0/7 (0) | 0/5 (0) | p>0.999 |  |
| O4 | rmp_a2 | 1/7 (14.286) | 0/5 (0) | p>0.999 |  |
| O4 | ybt only | 0/7 (0) | 5/5 (100) | p=0.001 |  |
| O4 | clb and not iuc | 0/7 (0) | 0/5 (0) | p>0.999 |  |
| O4 | iuc only | 0/7 (0) | 0/5 (0) | p>0.999 |  |
| O4 | icu and ybt w/o clb | 0/7 (0) | 0/5 (0) | p>0.999 |  |
| O4 | ybt, clb and iuc | 0/7 (0) | 0/5 (0) | p>0.999 |  |
| O5 | ybt | 17/20 (85) | 13/17 (76.471) | p=0.68 |  |
| O5 | clb | 0/20 (0) | 0/17 (0) | p>0.999 |  |
| O5 | iuc | 0/20 (0) | 0/17 (0) | p>0.999 |  |
| O5 | iro | 0/20 (0) | 0/17 (0) | p>0.999 |  |
| O5 | rmp_adc | 0/20 (0) | 0/17 (0) | p>0.999 |  |
| O5 | rmp_a2 | 0/20 (0) | 0/17 (0) | p>0.999 |  |
| O5 | ybt only | 17/20 (85) | 13/17 (76.471) | p=0.68 |  |
| O5 | clb and not iuc | 0/20 (0) | 0/17 (0) | p>0.999 |  |
| O5 | iuc only | 0/20 (0) | 0/17 (0) | p>0.999 |  |
| O5 | icu and ybt w/o clb | 0/20 (0) | 0/17 (0) | p>0.999 |  |
| O5 | ybt, clb and iuc | 0/20 (0) | 0/17 (0) | p>0.999 |  |
| **K-loci, n(%)** |  |  |  |  |  |
| KL10 | ybt | 1/2 (50) | 0/0 (0) | p>0.999 |  |
| KL10 | clb | 0/2 (0) | 0/0 (0) | p>0.999 |  |
| KL10 | iuc | 0/2 (0) | 0/0 (0) | p>0.999 |  |
| KL10 | iro | 0/2 (0) | 0/0 (0) | p>0.999 |  |
| KL10 | rmp_adc | 0/2 (0) | 0/0 (0) | p>0.999 |  |
| KL10 | rmp_a2 | 0/2 (0) | 0/0 (0) | p>0.999 |  |
| KL10 | ybt only | 1/2 (50) | 0/0 (0) | p>0.999 |  |
| KL10 | clb and not iuc | 0/2 (0) | 0/0 (0) | p>0.999 |  |
| KL10 | iuc only | 0/2 (0) | 0/0 (0) | p>0.999 |  |
| KL10 | icu and ybt w/o clb | 0/2 (0) | 0/0 (0) | p>0.999 |  |
| KL10 | ybt, clb and iuc | 0/2 (0) | 0/0 (0) | p>0.999 |  |
| KL102 | ybt | 0/8 (0) | 0/19 (0) | p>0.999 |  |
| KL102 | clb | 0/8 (0) | 0/19 (0) | p>0.999 |  |
| KL102 | iuc | 0/8 (0) | 0/19 (0) | p>0.999 |  |
| KL102 | iro | 0/8 (0) | 0/19 (0) | p>0.999 |  |
| KL102 | rmp_adc | 0/8 (0) | 0/19 (0) | p>0.999 |  |
| KL102 | rmp_a2 | 0/8 (0) | 0/19 (0) | p>0.999 |  |
| KL102 | ybt only | 0/8 (0) | 0/19 (0) | p>0.999 |  |
| KL102 | clb and not iuc | 0/8 (0) | 0/19 (0) | p>0.999 |  |
| KL102 | iuc only | 0/8 (0) | 0/19 (0) | p>0.999 |  |
| KL102 | icu and ybt w/o clb | 0/8 (0) | 0/19 (0) | p>0.999 |  |
| KL102 | ybt, clb and iuc | 0/8 (0) | 0/19 (0) | p>0.999 |  |
| KL103 | ybt | 0/1 (0) | 0/0 (0) | p>0.999 |  |
| KL103 | clb | 0/1 (0) | 0/0 (0) | p>0.999 |  |
| KL103 | iuc | 0/1 (0) | 0/0 (0) | p>0.999 |  |
| KL103 | iro | 0/1 (0) | 0/0 (0) | p>0.999 |  |
| KL103 | rmp_adc | 0/1 (0) | 0/0 (0) | p>0.999 |  |
| KL103 | rmp_a2 | 0/1 (0) | 0/0 (0) | p>0.999 |  |
| KL103 | ybt only | 0/1 (0) | 0/0 (0) | p>0.999 |  |
| KL103 | clb and not iuc | 0/1 (0) | 0/0 (0) | p>0.999 |  |
| KL103 | iuc only | 0/1 (0) | 0/0 (0) | p>0.999 |  |
| KL103 | icu and ybt w/o clb | 0/1 (0) | 0/0 (0) | p>0.999 |  |
| KL103 | ybt, clb and iuc | 0/1 (0) | 0/0 (0) | p>0.999 |  |
| KL106 | ybt | 0/1 (0) | 0/0 (0) | p>0.999 |  |
| KL106 | clb | 0/1 (0) | 0/0 (0) | p>0.999 |  |
| KL106 | iuc | 0/1 (0) | 0/0 (0) | p>0.999 |  |
| KL106 | iro | 0/1 (0) | 0/0 (0) | p>0.999 |  |
| KL106 | rmp_adc | 0/1 (0) | 0/0 (0) | p>0.999 |  |
| KL106 | rmp_a2 | 0/1 (0) | 0/0 (0) | p>0.999 |  |
| KL106 | ybt only | 0/1 (0) | 0/0 (0) | p>0.999 |  |
| KL106 | clb and not iuc | 0/1 (0) | 0/0 (0) | p>0.999 |  |
| KL106 | iuc only | 0/1 (0) | 0/0 (0) | p>0.999 |  |
| KL106 | icu and ybt w/o clb | 0/1 (0) | 0/0 (0) | p>0.999 |  |
| KL106 | ybt, clb and iuc | 0/1 (0) | 0/0 (0) | p>0.999 |  |
| KL108 | ybt | 0/0 (0) | 0/1 (0) | p>0.999 |  |
| KL108 | clb | 0/0 (0) | 0/1 (0) | p>0.999 |  |
| KL108 | iuc | 0/0 (0) | 0/1 (0) | p>0.999 |  |
| KL108 | iro | 0/0 (0) | 0/1 (0) | p>0.999 |  |
| KL108 | rmp_adc | 0/0 (0) | 0/1 (0) | p>0.999 |  |
| KL108 | rmp_a2 | 0/0 (0) | 0/1 (0) | p>0.999 |  |
| KL108 | ybt only | 0/0 (0) | 0/1 (0) | p>0.999 |  |
| KL108 | clb and not iuc | 0/0 (0) | 0/1 (0) | p>0.999 |  |
| KL108 | iuc only | 0/0 (0) | 0/1 (0) | p>0.999 |  |
| KL108 | icu and ybt w/o clb | 0/0 (0) | 0/1 (0) | p>0.999 |  |
| KL108 | ybt, clb and iuc | 0/0 (0) | 0/1 (0) | p>0.999 |  |
| KL110 | ybt | 1/2 (50) | 4/4 (100) | p=0.333 |  |
| KL110 | clb | 0/2 (0) | 0/4 (0) | p>0.999 |  |
| KL110 | iuc | 0/2 (0) | 0/4 (0) | p>0.999 |  |
| KL110 | iro | 0/2 (0) | 0/4 (0) | p>0.999 |  |
| KL110 | rmp_adc | 0/2 (0) | 0/4 (0) | p>0.999 |  |
| KL110 | rmp_a2 | 0/2 (0) | 0/4 (0) | p>0.999 |  |
| KL110 | ybt only | 1/2 (50) | 4/4 (100) | p=0.333 |  |
| KL110 | clb and not iuc | 0/2 (0) | 0/4 (0) | p>0.999 |  |
| KL110 | iuc only | 0/2 (0) | 0/4 (0) | p>0.999 |  |
| KL110 | icu and ybt w/o clb | 0/2 (0) | 0/4 (0) | p>0.999 |  |
| KL110 | ybt, clb and iuc | 0/2 (0) | 0/4 (0) | p>0.999 |  |
| KL112 | ybt | 0/0 (0) | 1/1 (100) | p>0.999 |  |
| KL112 | clb | 0/0 (0) | 0/1 (0) | p>0.999 |  |
| KL112 | iuc | 0/0 (0) | 0/1 (0) | p>0.999 |  |
| KL112 | iro | 0/0 (0) | 0/1 (0) | p>0.999 |  |
| KL112 | rmp_adc | 0/0 (0) | 0/1 (0) | p>0.999 |  |
| KL112 | rmp_a2 | 0/0 (0) | 0/1 (0) | p>0.999 |  |
| KL112 | ybt only | 0/0 (0) | 1/1 (100) | p>0.999 |  |
| KL112 | clb and not iuc | 0/0 (0) | 0/1 (0) | p>0.999 |  |
| KL112 | iuc only | 0/0 (0) | 0/1 (0) | p>0.999 |  |
| KL112 | icu and ybt w/o clb | 0/0 (0) | 0/1 (0) | p>0.999 |  |
| KL112 | ybt, clb and iuc | 0/0 (0) | 0/1 (0) | p>0.999 |  |
| KL116 | ybt | 2/2 (100) | 0/0 (0) | p>0.999 |  |
| KL116 | clb | 0/2 (0) | 0/0 (0) | p>0.999 |  |
| KL116 | iuc | 0/2 (0) | 0/0 (0) | p>0.999 |  |
| KL116 | iro | 0/2 (0) | 0/0 (0) | p>0.999 |  |
| KL116 | rmp_adc | 0/2 (0) | 0/0 (0) | p>0.999 |  |
| KL116 | rmp_a2 | 0/2 (0) | 0/0 (0) | p>0.999 |  |
| KL116 | ybt only | 2/2 (100) | 0/0 (0) | p>0.999 |  |
| KL116 | clb and not iuc | 0/2 (0) | 0/0 (0) | p>0.999 |  |
| KL116 | iuc only | 0/2 (0) | 0/0 (0) | p>0.999 |  |
| KL116 | icu and ybt w/o clb | 0/2 (0) | 0/0 (0) | p>0.999 |  |
| KL116 | ybt, clb and iuc | 0/2 (0) | 0/0 (0) | p>0.999 |  |
| KL12 | ybt | 0/1 (0) | 0/0 (0) | p>0.999 |  |
| KL12 | clb | 0/1 (0) | 0/0 (0) | p>0.999 |  |
| KL12 | iuc | 0/1 (0) | 0/0 (0) | p>0.999 |  |
| KL12 | iro | 0/1 (0) | 0/0 (0) | p>0.999 |  |
| KL12 | rmp_adc | 0/1 (0) | 0/0 (0) | p>0.999 |  |
| KL12 | rmp_a2 | 0/1 (0) | 0/0 (0) | p>0.999 |  |
| KL12 | ybt only | 0/1 (0) | 0/0 (0) | p>0.999 |  |
| KL12 | clb and not iuc | 0/1 (0) | 0/0 (0) | p>0.999 |  |
| KL12 | iuc only | 0/1 (0) | 0/0 (0) | p>0.999 |  |
| KL12 | icu and ybt w/o clb | 0/1 (0) | 0/0 (0) | p>0.999 |  |
| KL12 | ybt, clb and iuc | 0/1 (0) | 0/0 (0) | p>0.999 |  |
| KL125 | ybt | 0/1 (0) | 0/0 (0) | p>0.999 |  |
| KL125 | clb | 0/1 (0) | 0/0 (0) | p>0.999 |  |
| KL125 | iuc | 0/1 (0) | 0/0 (0) | p>0.999 |  |
| KL125 | iro | 0/1 (0) | 0/0 (0) | p>0.999 |  |
| KL125 | rmp_adc | 0/1 (0) | 0/0 (0) | p>0.999 |  |
| KL125 | rmp_a2 | 0/1 (0) | 0/0 (0) | p>0.999 |  |
| KL125 | ybt only | 0/1 (0) | 0/0 (0) | p>0.999 |  |
| KL125 | clb and not iuc | 0/1 (0) | 0/0 (0) | p>0.999 |  |
| KL125 | iuc only | 0/1 (0) | 0/0 (0) | p>0.999 |  |
| KL125 | icu and ybt w/o clb | 0/1 (0) | 0/0 (0) | p>0.999 |  |
| KL125 | ybt, clb and iuc | 0/1 (0) | 0/0 (0) | p>0.999 |  |
| KL128 | ybt | 0/1 (0) | 0/0 (0) | p>0.999 |  |
| KL128 | clb | 0/1 (0) | 0/0 (0) | p>0.999 |  |
| KL128 | iuc | 0/1 (0) | 0/0 (0) | p>0.999 |  |
| KL128 | iro | 0/1 (0) | 0/0 (0) | p>0.999 |  |
| KL128 | rmp_adc | 0/1 (0) | 0/0 (0) | p>0.999 |  |
| KL128 | rmp_a2 | 0/1 (0) | 0/0 (0) | p>0.999 |  |
| KL128 | ybt only | 0/1 (0) | 0/0 (0) | p>0.999 |  |
| KL128 | clb and not iuc | 0/1 (0) | 0/0 (0) | p>0.999 |  |
| KL128 | iuc only | 0/1 (0) | 0/0 (0) | p>0.999 |  |
| KL128 | icu and ybt w/o clb | 0/1 (0) | 0/0 (0) | p>0.999 |  |
| KL128 | ybt, clb and iuc | 0/1 (0) | 0/0 (0) | p>0.999 |  |
| KL140 | ybt | 0/1 (0) | 0/0 (0) | p>0.999 |  |
| KL140 | clb | 0/1 (0) | 0/0 (0) | p>0.999 |  |
| KL140 | iuc | 0/1 (0) | 0/0 (0) | p>0.999 |  |
| KL140 | iro | 0/1 (0) | 0/0 (0) | p>0.999 |  |
| KL140 | rmp_adc | 0/1 (0) | 0/0 (0) | p>0.999 |  |
| KL140 | rmp_a2 | 0/1 (0) | 0/0 (0) | p>0.999 |  |
| KL140 | ybt only | 0/1 (0) | 0/0 (0) | p>0.999 |  |
| KL140 | clb and not iuc | 0/1 (0) | 0/0 (0) | p>0.999 |  |
| KL140 | iuc only | 0/1 (0) | 0/0 (0) | p>0.999 |  |
| KL140 | icu and ybt w/o clb | 0/1 (0) | 0/0 (0) | p>0.999 |  |
| KL140 | ybt, clb and iuc | 0/1 (0) | 0/0 (0) | p>0.999 |  |
| KL142 | ybt | 0/1 (0) | 0/0 (0) | p>0.999 |  |
| KL142 | clb | 0/1 (0) | 0/0 (0) | p>0.999 |  |
| KL142 | iuc | 0/1 (0) | 0/0 (0) | p>0.999 |  |
| KL142 | iro | 0/1 (0) | 0/0 (0) | p>0.999 |  |
| KL142 | rmp_adc | 0/1 (0) | 0/0 (0) | p>0.999 |  |
| KL142 | rmp_a2 | 0/1 (0) | 0/0 (0) | p>0.999 |  |
| KL142 | ybt only | 0/1 (0) | 0/0 (0) | p>0.999 |  |
| KL142 | clb and not iuc | 0/1 (0) | 0/0 (0) | p>0.999 |  |
| KL142 | iuc only | 0/1 (0) | 0/0 (0) | p>0.999 |  |
| KL142 | icu and ybt w/o clb | 0/1 (0) | 0/0 (0) | p>0.999 |  |
| KL142 | ybt, clb and iuc | 0/1 (0) | 0/0 (0) | p>0.999 |  |
| KL149 | ybt | 0/0 (0) | 28/28 (100) | p>0.999 |  |
| KL149 | clb | 0/0 (0) | 0/28 (0) | p>0.999 |  |
| KL149 | iuc | 0/0 (0) | 0/28 (0) | p>0.999 |  |
| KL149 | iro | 0/0 (0) | 0/28 (0) | p>0.999 |  |
| KL149 | rmp_adc | 0/0 (0) | 0/28 (0) | p>0.999 |  |
| KL149 | rmp_a2 | 0/0 (0) | 0/28 (0) | p>0.999 |  |
| KL149 | ybt only | 0/0 (0) | 28/28 (100) | p>0.999 |  |
| KL149 | clb and not iuc | 0/0 (0) | 0/28 (0) | p>0.999 |  |
| KL149 | iuc only | 0/0 (0) | 0/28 (0) | p>0.999 |  |
| KL149 | icu and ybt w/o clb | 0/0 (0) | 0/28 (0) | p>0.999 |  |
| KL149 | ybt, clb and iuc | 0/0 (0) | 0/28 (0) | p>0.999 |  |
| KL15 | ybt | 0/5 (0) | 0/0 (0) | p>0.999 |  |
| KL15 | clb | 0/5 (0) | 0/0 (0) | p>0.999 |  |
| KL15 | iuc | 0/5 (0) | 0/0 (0) | p>0.999 |  |
| KL15 | iro | 0/5 (0) | 0/0 (0) | p>0.999 |  |
| KL15 | rmp_adc | 0/5 (0) | 0/0 (0) | p>0.999 |  |
| KL15 | rmp_a2 | 0/5 (0) | 0/0 (0) | p>0.999 |  |
| KL15 | ybt only | 0/5 (0) | 0/0 (0) | p>0.999 |  |
| KL15 | clb and not iuc | 0/5 (0) | 0/0 (0) | p>0.999 |  |
| KL15 | iuc only | 0/5 (0) | 0/0 (0) | p>0.999 |  |
| KL15 | icu and ybt w/o clb | 0/5 (0) | 0/0 (0) | p>0.999 |  |
| KL15 | ybt, clb and iuc | 0/5 (0) | 0/0 (0) | p>0.999 |  |
| KL158 | ybt | 0/1 (0) | 0/0 (0) | p>0.999 |  |
| KL158 | clb | 0/1 (0) | 0/0 (0) | p>0.999 |  |
| KL158 | iuc | 0/1 (0) | 0/0 (0) | p>0.999 |  |
| KL158 | iro | 0/1 (0) | 0/0 (0) | p>0.999 |  |
| KL158 | rmp_adc | 0/1 (0) | 0/0 (0) | p>0.999 |  |
| KL158 | rmp_a2 | 0/1 (0) | 0/0 (0) | p>0.999 |  |
| KL158 | ybt only | 0/1 (0) | 0/0 (0) | p>0.999 |  |
| KL158 | clb and not iuc | 0/1 (0) | 0/0 (0) | p>0.999 |  |
| KL158 | iuc only | 0/1 (0) | 0/0 (0) | p>0.999 |  |
| KL158 | icu and ybt w/o clb | 0/1 (0) | 0/0 (0) | p>0.999 |  |
| KL158 | ybt, clb and iuc | 0/1 (0) | 0/0 (0) | p>0.999 |  |
| KL16 | ybt | 0/0 (0) | 1/1 (100) | p>0.999 |  |
| KL16 | clb | 0/0 (0) | 0/1 (0) | p>0.999 |  |
| KL16 | iuc | 0/0 (0) | 0/1 (0) | p>0.999 |  |
| KL16 | iro | 0/0 (0) | 0/1 (0) | p>0.999 |  |
| KL16 | rmp_adc | 0/0 (0) | 0/1 (0) | p>0.999 |  |
| KL16 | rmp_a2 | 0/0 (0) | 0/1 (0) | p>0.999 |  |
| KL16 | ybt only | 0/0 (0) | 1/1 (100) | p>0.999 |  |
| KL16 | clb and not iuc | 0/0 (0) | 0/1 (0) | p>0.999 |  |
| KL16 | iuc only | 0/0 (0) | 0/1 (0) | p>0.999 |  |
| KL16 | icu and ybt w/o clb | 0/0 (0) | 0/1 (0) | p>0.999 |  |
| KL16 | ybt, clb and iuc | 0/0 (0) | 0/1 (0) | p>0.999 |  |
| KL17 | ybt | 0/22 (0) | 0/0 (0) | p>0.999 |  |
| KL17 | clb | 0/22 (0) | 0/0 (0) | p>0.999 |  |
| KL17 | iuc | 0/22 (0) | 0/0 (0) | p>0.999 |  |
| KL17 | iro | 0/22 (0) | 0/0 (0) | p>0.999 |  |
| KL17 | rmp_adc | 0/22 (0) | 0/0 (0) | p>0.999 |  |
| KL17 | rmp_a2 | 0/22 (0) | 0/0 (0) | p>0.999 |  |
| KL17 | ybt only | 0/22 (0) | 0/0 (0) | p>0.999 |  |
| KL17 | clb and not iuc | 0/22 (0) | 0/0 (0) | p>0.999 |  |
| KL17 | iuc only | 0/22 (0) | 0/0 (0) | p>0.999 |  |
| KL17 | icu and ybt w/o clb | 0/22 (0) | 0/0 (0) | p>0.999 |  |
| KL17 | ybt, clb and iuc | 0/22 (0) | 0/0 (0) | p>0.999 |  |
| KL2 | ybt | 0/0 (0) | 8/9 (88.889) | p>0.999 |  |
| KL2 | clb | 0/0 (0) | 1/9 (11.111) | p>0.999 |  |
| KL2 | iuc | 0/0 (0) | 1/9 (11.111) | p>0.999 |  |
| KL2 | iro | 0/0 (0) | 1/9 (11.111) | p>0.999 |  |
| KL2 | rmp_adc | 0/0 (0) | 1/9 (11.111) | p>0.999 |  |
| KL2 | rmp_a2 | 0/0 (0) | 1/9 (11.111) | p>0.999 |  |
| KL2 | ybt only | 0/0 (0) | 7/9 (77.778) | p>0.999 |  |
| KL2 | clb and not iuc | 0/0 (0) | 0/9 (0) | p>0.999 |  |
| KL2 | iuc only | 0/0 (0) | 0/9 (0) | p>0.999 |  |
| KL2 | icu and ybt w/o clb | 0/0 (0) | 0/9 (0) | p>0.999 |  |
| KL2 | ybt, clb and iuc | 0/0 (0) | 1/9 (11.111) | p>0.999 |  |
| KL24 | ybt | 0/4 (0) | 0/0 (0) | p>0.999 |  |
| KL24 | clb | 0/4 (0) | 0/0 (0) | p>0.999 |  |
| KL24 | iuc | 0/4 (0) | 0/0 (0) | p>0.999 |  |
| KL24 | iro | 0/4 (0) | 0/0 (0) | p>0.999 |  |
| KL24 | rmp_adc | 0/4 (0) | 0/0 (0) | p>0.999 |  |
| KL24 | rmp_a2 | 0/4 (0) | 0/0 (0) | p>0.999 |  |
| KL24 | ybt only | 0/4 (0) | 0/0 (0) | p>0.999 |  |
| KL24 | clb and not iuc | 0/4 (0) | 0/0 (0) | p>0.999 |  |
| KL24 | iuc only | 0/4 (0) | 0/0 (0) | p>0.999 |  |
| KL24 | icu and ybt w/o clb | 0/4 (0) | 0/0 (0) | p>0.999 |  |
| KL24 | ybt, clb and iuc | 0/4 (0) | 0/0 (0) | p>0.999 |  |
| KL25 | ybt | 17/20 (85) | 14/21 (66.667) | p=0.277 |  |
| KL25 | clb | 0/20 (0) | 0/21 (0) | p>0.999 |  |
| KL25 | iuc | 0/20 (0) | 0/21 (0) | p>0.999 |  |
| KL25 | iro | 0/20 (0) | 0/21 (0) | p>0.999 |  |
| KL25 | rmp_adc | 0/20 (0) | 0/21 (0) | p>0.999 |  |
| KL25 | rmp_a2 | 0/20 (0) | 0/21 (0) | p>0.999 |  |
| KL25 | ybt only | 17/20 (85) | 14/21 (66.667) | p=0.277 |  |
| KL25 | clb and not iuc | 0/20 (0) | 0/21 (0) | p>0.999 |  |
| KL25 | iuc only | 0/20 (0) | 0/21 (0) | p>0.999 |  |
| KL25 | icu and ybt w/o clb | 0/20 (0) | 0/21 (0) | p>0.999 |  |
| KL25 | ybt, clb and iuc | 0/20 (0) | 0/21 (0) | p>0.999 |  |
| KL28 | ybt | 0/0 (0) | 0/2 (0) | p>0.999 |  |
| KL28 | clb | 0/0 (0) | 0/2 (0) | p>0.999 |  |
| KL28 | iuc | 0/0 (0) | 0/2 (0) | p>0.999 |  |
| KL28 | iro | 0/0 (0) | 0/2 (0) | p>0.999 |  |
| KL28 | rmp_adc | 0/0 (0) | 0/2 (0) | p>0.999 |  |
| KL28 | rmp_a2 | 0/0 (0) | 0/2 (0) | p>0.999 |  |
| KL28 | ybt only | 0/0 (0) | 0/2 (0) | p>0.999 |  |
| KL28 | clb and not iuc | 0/0 (0) | 0/2 (0) | p>0.999 |  |
| KL28 | iuc only | 0/0 (0) | 0/2 (0) | p>0.999 |  |
| KL28 | icu and ybt w/o clb | 0/0 (0) | 0/2 (0) | p>0.999 |  |
| KL28 | ybt, clb and iuc | 0/0 (0) | 0/2 (0) | p>0.999 |  |
| KL3 | ybt | 2/2 (100) | 3/3 (100) | p>0.999 |  |
| KL3 | clb | 0/2 (0) | 0/3 (0) | p>0.999 |  |
| KL3 | iuc | 0/2 (0) | 0/3 (0) | p>0.999 |  |
| KL3 | iro | 0/2 (0) | 0/3 (0) | p>0.999 |  |
| KL3 | rmp_adc | 0/2 (0) | 0/3 (0) | p>0.999 |  |
| KL3 | rmp_a2 | 0/2 (0) | 0/3 (0) | p>0.999 |  |
| KL3 | ybt only | 2/2 (100) | 3/3 (100) | p>0.999 |  |
| KL3 | clb and not iuc | 0/2 (0) | 0/3 (0) | p>0.999 |  |
| KL3 | iuc only | 0/2 (0) | 0/3 (0) | p>0.999 |  |
| KL3 | icu and ybt w/o clb | 0/2 (0) | 0/3 (0) | p>0.999 |  |
| KL3 | ybt, clb and iuc | 0/2 (0) | 0/3 (0) | p>0.999 |  |
| KL30 | ybt | 0/1 (0) | 0/2 (0) | p>0.999 |  |
| KL30 | clb | 0/1 (0) | 0/2 (0) | p>0.999 |  |
| KL30 | iuc | 0/1 (0) | 0/2 (0) | p>0.999 |  |
| KL30 | iro | 0/1 (0) | 0/2 (0) | p>0.999 |  |
| KL30 | rmp_adc | 0/1 (0) | 0/2 (0) | p>0.999 |  |
| KL30 | rmp_a2 | 0/1 (0) | 0/2 (0) | p>0.999 |  |
| KL30 | ybt only | 0/1 (0) | 0/2 (0) | p>0.999 |  |
| KL30 | clb and not iuc | 0/1 (0) | 0/2 (0) | p>0.999 |  |
| KL30 | iuc only | 0/1 (0) | 0/2 (0) | p>0.999 |  |
| KL30 | icu and ybt w/o clb | 0/1 (0) | 0/2 (0) | p>0.999 |  |
| KL30 | ybt, clb and iuc | 0/1 (0) | 0/2 (0) | p>0.999 |  |
| KL38 | ybt | 1/1 (100) | 0/0 (0) | p>0.999 |  |
| KL38 | clb | 0/1 (0) | 0/0 (0) | p>0.999 |  |
| KL38 | iuc | 0/1 (0) | 0/0 (0) | p>0.999 |  |
| KL38 | iro | 0/1 (0) | 0/0 (0) | p>0.999 |  |
| KL38 | rmp_adc | 0/1 (0) | 0/0 (0) | p>0.999 |  |
| KL38 | rmp_a2 | 0/1 (0) | 0/0 (0) | p>0.999 |  |
| KL38 | ybt only | 1/1 (100) | 0/0 (0) | p>0.999 |  |
| KL38 | clb and not iuc | 0/1 (0) | 0/0 (0) | p>0.999 |  |
| KL38 | iuc only | 0/1 (0) | 0/0 (0) | p>0.999 |  |
| KL38 | icu and ybt w/o clb | 0/1 (0) | 0/0 (0) | p>0.999 |  |
| KL38 | ybt, clb and iuc | 0/1 (0) | 0/0 (0) | p>0.999 |  |
| KL39 | ybt | 0/1 (0) | 0/0 (0) | p>0.999 |  |
| KL39 | clb | 0/1 (0) | 0/0 (0) | p>0.999 |  |
| KL39 | iuc | 0/1 (0) | 0/0 (0) | p>0.999 |  |
| KL39 | iro | 0/1 (0) | 0/0 (0) | p>0.999 |  |
| KL39 | rmp_adc | 0/1 (0) | 0/0 (0) | p>0.999 |  |
| KL39 | rmp_a2 | 0/1 (0) | 0/0 (0) | p>0.999 |  |
| KL39 | ybt only | 0/1 (0) | 0/0 (0) | p>0.999 |  |
| KL39 | clb and not iuc | 0/1 (0) | 0/0 (0) | p>0.999 |  |
| KL39 | iuc only | 0/1 (0) | 0/0 (0) | p>0.999 |  |
| KL39 | icu and ybt w/o clb | 0/1 (0) | 0/0 (0) | p>0.999 |  |
| KL39 | ybt, clb and iuc | 0/1 (0) | 0/0 (0) | p>0.999 |  |
| KL42 | ybt | 0/1 (0) | 0/0 (0) | p>0.999 |  |
| KL42 | clb | 0/1 (0) | 0/0 (0) | p>0.999 |  |
| KL42 | iuc | 0/1 (0) | 0/0 (0) | p>0.999 |  |
| KL42 | iro | 0/1 (0) | 0/0 (0) | p>0.999 |  |
| KL42 | rmp_adc | 0/1 (0) | 0/0 (0) | p>0.999 |  |
| KL42 | rmp_a2 | 0/1 (0) | 0/0 (0) | p>0.999 |  |
| KL42 | ybt only | 0/1 (0) | 0/0 (0) | p>0.999 |  |
| KL42 | clb and not iuc | 0/1 (0) | 0/0 (0) | p>0.999 |  |
| KL42 | iuc only | 0/1 (0) | 0/0 (0) | p>0.999 |  |
| KL42 | icu and ybt w/o clb | 0/1 (0) | 0/0 (0) | p>0.999 |  |
| KL42 | ybt, clb and iuc | 0/1 (0) | 0/0 (0) | p>0.999 |  |
| KL45 | ybt | 0/1 (0) | 0/0 (0) | p>0.999 |  |
| KL45 | clb | 0/1 (0) | 0/0 (0) | p>0.999 |  |
| KL45 | iuc | 0/1 (0) | 0/0 (0) | p>0.999 |  |
| KL45 | iro | 1/1 (100) | 0/0 (0) | p>0.999 |  |
| KL45 | rmp_adc | 0/1 (0) | 0/0 (0) | p>0.999 |  |
| KL45 | rmp_a2 | 1/1 (100) | 0/0 (0) | p>0.999 |  |
| KL45 | ybt only | 0/1 (0) | 0/0 (0) | p>0.999 |  |
| KL45 | clb and not iuc | 0/1 (0) | 0/0 (0) | p>0.999 |  |
| KL45 | iuc only | 0/1 (0) | 0/0 (0) | p>0.999 |  |
| KL45 | icu and ybt w/o clb | 0/1 (0) | 0/0 (0) | p>0.999 |  |
| KL45 | ybt, clb and iuc | 0/1 (0) | 0/0 (0) | p>0.999 |  |
| KL46 | ybt | 0/0 (0) | 0/1 (0) | p>0.999 |  |
| KL46 | clb | 0/0 (0) | 0/1 (0) | p>0.999 |  |
| KL46 | iuc | 0/0 (0) | 0/1 (0) | p>0.999 |  |
| KL46 | iro | 0/0 (0) | 0/1 (0) | p>0.999 |  |
| KL46 | rmp_adc | 0/0 (0) | 0/1 (0) | p>0.999 |  |
| KL46 | rmp_a2 | 0/0 (0) | 0/1 (0) | p>0.999 |  |
| KL46 | ybt only | 0/0 (0) | 0/1 (0) | p>0.999 |  |
| KL46 | clb and not iuc | 0/0 (0) | 0/1 (0) | p>0.999 |  |
| KL46 | iuc only | 0/0 (0) | 0/1 (0) | p>0.999 |  |
| KL46 | icu and ybt w/o clb | 0/0 (0) | 0/1 (0) | p>0.999 |  |
| KL46 | ybt, clb and iuc | 0/0 (0) | 0/1 (0) | p>0.999 |  |
| KL51 | ybt | 0/1 (0) | 1/1 (100) | p>0.999 |  |
| KL51 | clb | 0/1 (0) | 0/1 (0) | p>0.999 |  |
| KL51 | iuc | 0/1 (0) | 0/1 (0) | p>0.999 |  |
| KL51 | iro | 0/1 (0) | 0/1 (0) | p>0.999 |  |
| KL51 | rmp_adc | 0/1 (0) | 0/1 (0) | p>0.999 |  |
| KL51 | rmp_a2 | 0/1 (0) | 0/1 (0) | p>0.999 |  |
| KL51 | ybt only | 0/1 (0) | 1/1 (100) | p>0.999 |  |
| KL51 | clb and not iuc | 0/1 (0) | 0/1 (0) | p>0.999 |  |
| KL51 | iuc only | 0/1 (0) | 0/1 (0) | p>0.999 |  |
| KL51 | icu and ybt w/o clb | 0/1 (0) | 0/1 (0) | p>0.999 |  |
| KL51 | ybt, clb and iuc | 0/1 (0) | 0/1 (0) | p>0.999 |  |
| KL60 | ybt | 0/1 (0) | 0/0 (0) | p>0.999 |  |
| KL60 | clb | 0/1 (0) | 0/0 (0) | p>0.999 |  |
| KL60 | iuc | 0/1 (0) | 0/0 (0) | p>0.999 |  |
| KL60 | iro | 0/1 (0) | 0/0 (0) | p>0.999 |  |
| KL60 | rmp_adc | 0/1 (0) | 0/0 (0) | p>0.999 |  |
| KL60 | rmp_a2 | 0/1 (0) | 0/0 (0) | p>0.999 |  |
| KL60 | ybt only | 0/1 (0) | 0/0 (0) | p>0.999 |  |
| KL60 | clb and not iuc | 0/1 (0) | 0/0 (0) | p>0.999 |  |
| KL60 | iuc only | 0/1 (0) | 0/0 (0) | p>0.999 |  |
| KL60 | icu and ybt w/o clb | 0/1 (0) | 0/0 (0) | p>0.999 |  |
| KL60 | ybt, clb and iuc | 0/1 (0) | 0/0 (0) | p>0.999 |  |
| KL61 | ybt | 0/1 (0) | 0/0 (0) | p>0.999 |  |
| KL61 | clb | 0/1 (0) | 0/0 (0) | p>0.999 |  |
| KL61 | iuc | 0/1 (0) | 0/0 (0) | p>0.999 |  |
| KL61 | iro | 0/1 (0) | 0/0 (0) | p>0.999 |  |
| KL61 | rmp_adc | 0/1 (0) | 0/0 (0) | p>0.999 |  |
| KL61 | rmp_a2 | 0/1 (0) | 0/0 (0) | p>0.999 |  |
| KL61 | ybt only | 0/1 (0) | 0/0 (0) | p>0.999 |  |
| KL61 | clb and not iuc | 0/1 (0) | 0/0 (0) | p>0.999 |  |
| KL61 | iuc only | 0/1 (0) | 0/0 (0) | p>0.999 |  |
| KL61 | icu and ybt w/o clb | 0/1 (0) | 0/0 (0) | p>0.999 |  |
| KL61 | ybt, clb and iuc | 0/1 (0) | 0/0 (0) | p>0.999 |  |
| KL62 | ybt | 0/1 (0) | 0/3 (0) | p>0.999 |  |
| KL62 | clb | 0/1 (0) | 0/3 (0) | p>0.999 |  |
| KL62 | iuc | 0/1 (0) | 0/3 (0) | p>0.999 |  |
| KL62 | iro | 0/1 (0) | 0/3 (0) | p>0.999 |  |
| KL62 | rmp_adc | 0/1 (0) | 0/3 (0) | p>0.999 |  |
| KL62 | rmp_a2 | 0/1 (0) | 0/3 (0) | p>0.999 |  |
| KL62 | ybt only | 0/1 (0) | 0/3 (0) | p>0.999 |  |
| KL62 | clb and not iuc | 0/1 (0) | 0/3 (0) | p>0.999 |  |
| KL62 | iuc only | 0/1 (0) | 0/3 (0) | p>0.999 |  |
| KL62 | icu and ybt w/o clb | 0/1 (0) | 0/3 (0) | p>0.999 |  |
| KL62 | ybt, clb and iuc | 0/1 (0) | 0/3 (0) | p>0.999 |  |
| KL63 | ybt | 0/1 (0) | 0/0 (0) | p>0.999 |  |
| KL63 | clb | 0/1 (0) | 0/0 (0) | p>0.999 |  |
| KL63 | iuc | 0/1 (0) | 0/0 (0) | p>0.999 |  |
| KL63 | iro | 0/1 (0) | 0/0 (0) | p>0.999 |  |
| KL63 | rmp_adc | 0/1 (0) | 0/0 (0) | p>0.999 |  |
| KL63 | rmp_a2 | 0/1 (0) | 0/0 (0) | p>0.999 |  |
| KL63 | ybt only | 0/1 (0) | 0/0 (0) | p>0.999 |  |
| KL63 | clb and not iuc | 0/1 (0) | 0/0 (0) | p>0.999 |  |
| KL63 | iuc only | 0/1 (0) | 0/0 (0) | p>0.999 |  |
| KL63 | icu and ybt w/o clb | 0/1 (0) | 0/0 (0) | p>0.999 |  |
| KL63 | ybt, clb and iuc | 0/1 (0) | 0/0 (0) | p>0.999 |  |
| KL7 | ybt | 0/0 (0) | 0/1 (0) | p>0.999 |  |
| KL7 | clb | 0/0 (0) | 0/1 (0) | p>0.999 |  |
| KL7 | iuc | 0/0 (0) | 0/1 (0) | p>0.999 |  |
| KL7 | iro | 0/0 (0) | 0/1 (0) | p>0.999 |  |
| KL7 | rmp_adc | 0/0 (0) | 0/1 (0) | p>0.999 |  |
| KL7 | rmp_a2 | 0/0 (0) | 0/1 (0) | p>0.999 |  |
| KL7 | ybt only | 0/0 (0) | 0/1 (0) | p>0.999 |  |
| KL7 | clb and not iuc | 0/0 (0) | 0/1 (0) | p>0.999 |  |
| KL7 | iuc only | 0/0 (0) | 0/1 (0) | p>0.999 |  |
| KL7 | icu and ybt w/o clb | 0/0 (0) | 0/1 (0) | p>0.999 |  |
| KL7 | ybt, clb and iuc | 0/0 (0) | 0/1 (0) | p>0.999 |  |
| KL8 | ybt | 6/7 (85.714) | 0/1 (0) | p=0.25 |  |
| KL8 | clb | 0/7 (0) | 0/1 (0) | p>0.999 |  |
| KL8 | iuc | 0/7 (0) | 0/1 (0) | p>0.999 |  |
| KL8 | iro | 0/7 (0) | 0/1 (0) | p>0.999 |  |
| KL8 | rmp_adc | 0/7 (0) | 0/1 (0) | p>0.999 |  |
| KL8 | rmp_a2 | 0/7 (0) | 0/1 (0) | p>0.999 |  |
| KL8 | ybt only | 6/7 (85.714) | 0/1 (0) | p=0.25 |  |
| KL8 | clb and not iuc | 0/7 (0) | 0/1 (0) | p>0.999 |  |
| KL8 | iuc only | 0/7 (0) | 0/1 (0) | p>0.999 |  |
| KL8 | icu and ybt w/o clb | 0/7 (0) | 0/1 (0) | p>0.999 |  |
| KL8 | ybt, clb and iuc | 0/7 (0) | 0/1 (0) | p>0.999 |  |
| KL9 | ybt | 0/2 (0) | 0/0 (0) | p>0.999 |  |
| KL9 | clb | 0/2 (0) | 0/0 (0) | p>0.999 |  |
| KL9 | iuc | 0/2 (0) | 0/0 (0) | p>0.999 |  |
| KL9 | iro | 0/2 (0) | 0/0 (0) | p>0.999 |  |
| KL9 | rmp_adc | 0/2 (0) | 0/0 (0) | p>0.999 |  |
| KL9 | rmp_a2 | 0/2 (0) | 0/0 (0) | p>0.999 |  |
| KL9 | ybt only | 0/2 (0) | 0/0 (0) | p>0.999 |  |
| KL9 | clb and not iuc | 0/2 (0) | 0/0 (0) | p>0.999 |  |
| KL9 | iuc only | 0/2 (0) | 0/0 (0) | p>0.999 |  |
| KL9 | icu and ybt w/o clb | 0/2 (0) | 0/0 (0) | p>0.999 |  |
| KL9 | ybt, clb and iuc | 0/2 (0) | 0/0 (0) | p>0.999 |  |
|  |  |  |  |  |  |

Abbreviations: clb = colibactin, iuc = aerobactin, OR = odd ratio, ST = sequence type, ybt = yersiniabactin.

P-values of <0.05 are considered significant

**Supplementary Table 3** Antimicrobial resistance, by Sequence type, K-loci, O-loci, and O-antigen

|  |  | **Colonization (n=94)** | **Invasive (n=98)** | **P-value** |
| --- | --- | --- | --- | --- |
| **Sequence types, n (%)** |  |  |  |  |
| ST101 | Aminoglycosides | 21/22 (95.455) | 0/0 (0) | p>0.999 |
| ST101 | Penicillins | 22/22 (100) | 0/0 (0) | p>0.999 |
| ST101 | Cephalosporins | 20/22 (90.909) | 0/0 (0) | p>0.999 |
| ST101 | Carbapenems | 1/22 (4.545) | 0/0 (0) | p>0.999 |
| ST101 | Polymyxin | 1/22 (4.545) | 0/0 (0) | p>0.999 |
| ST101 | Fluoroquinolones | 20/22 (90.909) | 0/0 (0) | p>0.999 |
| ST101 | Sulfonamides | 21/22 (95.455) | 0/0 (0) | p>0.999 |
| ST101 | Tetracyclines | 22/22 (100) | 0/0 (0) | p>0.999 |
| ST101 | ESBL | 20/22 (90.909) | 0/0 (0) | p>0.999 |
| ST101 | MDR | 21/22 (95.455) | 0/0 (0) | p>0.999 |
| ST1026 | Aminoglycosides | 1/1 (100) | 0/0 (0) | p>0.999 |
| ST1026 | Penicillins | 1/1 (100) | 0/0 (0) | p>0.999 |
| ST1026 | Cephalosporins | 1/1 (100) | 0/0 (0) | p>0.999 |
| ST1026 | Carbapenems | 0/1 (0) | 0/0 (0) | p>0.999 |
| ST1026 | Polymyxin | 0/1 (0) | 0/0 (0) | p>0.999 |
| ST1026 | Fluoroquinolones | 0/1 (0) | 0/0 (0) | p>0.999 |
| ST1026 | Sulfonamides | 1/1 (100) | 0/0 (0) | p>0.999 |
| ST1026 | Tetracyclines | 0/1 (0) | 0/0 (0) | p>0.999 |
| ST1026 | ESBL | 1/1 (100) | 0/0 (0) | p>0.999 |
| ST1026 | MDR | 1/1 (100) | 0/0 (0) | p>0.999 |
| ST1119 | Aminoglycosides | 1/1 (100) | 0/0 (0) | p>0.999 |
| ST1119 | Penicillins | 1/1 (100) | 0/0 (0) | p>0.999 |
| ST1119 | Cephalosporins | 0/1 (0) | 0/0 (0) | p>0.999 |
| ST1119 | Carbapenems | 0/1 (0) | 0/0 (0) | p>0.999 |
| ST1119 | Polymyxin | 0/1 (0) | 0/0 (0) | p>0.999 |
| ST1119 | Fluoroquinolones | 0/1 (0) | 0/0 (0) | p>0.999 |
| ST1119 | Sulfonamides | 0/1 (0) | 0/0 (0) | p>0.999 |
| ST1119 | Tetracyclines | 0/1 (0) | 0/0 (0) | p>0.999 |
| ST1119 | ESBL | 0/1 (0) | 0/0 (0) | p>0.999 |
| ST1119 | MDR | 0/1 (0) | 0/0 (0) | p>0.999 |
| ST1263 | Aminoglycosides | 0/1 (0) | 0/0 (0) | p>0.999 |
| ST1263 | Penicillins | 0/1 (0) | 0/0 (0) | p>0.999 |
| ST1263 | Cephalosporins | 0/1 (0) | 0/0 (0) | p>0.999 |
| ST1263 | Carbapenems | 0/1 (0) | 0/0 (0) | p>0.999 |
| ST1263 | Polymyxin | 0/1 (0) | 0/0 (0) | p>0.999 |
| ST1263 | Fluoroquinolones | 0/1 (0) | 0/0 (0) | p>0.999 |
| ST1263 | Sulfonamides | 0/1 (0) | 0/0 (0) | p>0.999 |
| ST1263 | Tetracyclines | 0/1 (0) | 0/0 (0) | p>0.999 |
| ST1263 | ESBL | 0/1 (0) | 0/0 (0) | p>0.999 |
| ST1263 | MDR | 0/1 (0) | 0/0 (0) | p>0.999 |
| ST13 | Aminoglycosides | 2/2 (100) | 3/3 (100) | p>0.999 |
| ST13 | Penicillins | 2/2 (100) | 3/3 (100) | p>0.999 |
| ST13 | Cephalosporins | 0/2 (0) | 3/3 (100) | p=0.1 |
| ST13 | Carbapenems | 0/2 (0) | 0/3 (0) | p>0.999 |
| ST13 | Polymyxin | 0/2 (0) | 0/3 (0) | p>0.999 |
| ST13 | Fluoroquinolones | 0/2 (0) | 1/3 (33.333) | p>0.999 |
| ST13 | Sulfonamides | 2/2 (100) | 3/3 (100) | p>0.999 |
| ST13 | Tetracyclines | 2/2 (100) | 1/3 (33.333) | p=0.4 |
| ST13 | ESBL | 0/2 (0) | 3/3 (100) | p=0.1 |
| ST13 | MDR | 2/2 (100) | 3/3 (100) | p>0.999 |
| ST133 | Aminoglycosides | 3/3 (100) | 0/0 (0) | p>0.999 |
| ST133 | Penicillins | 3/3 (100) | 0/0 (0) | p>0.999 |
| ST133 | Cephalosporins | 2/3 (66.667) | 0/0 (0) | p>0.999 |
| ST133 | Carbapenems | 0/3 (0) | 0/0 (0) | p>0.999 |
| ST133 | Polymyxin | 0/3 (0) | 0/0 (0) | p>0.999 |
| ST133 | Fluoroquinolones | 2/3 (66.667) | 0/0 (0) | p>0.999 |
| ST133 | Sulfonamides | 2/3 (66.667) | 0/0 (0) | p>0.999 |
| ST133 | Tetracyclines | 0/3 (0) | 0/0 (0) | p>0.999 |
| ST133 | ESBL | 2/3 (66.667) | 0/0 (0) | p>0.999 |
| ST133 | MDR | 2/3 (66.667) | 0/0 (0) | p>0.999 |
| ST1380 | Aminoglycosides | 0/2 (0) | 0/0 (0) | p>0.999 |
| ST1380 | Penicillins | 2/2 (100) | 0/0 (0) | p>0.999 |
| ST1380 | Cephalosporins | 0/2 (0) | 0/0 (0) | p>0.999 |
| ST1380 | Carbapenems | 0/2 (0) | 0/0 (0) | p>0.999 |
| ST1380 | Polymyxin | 0/2 (0) | 0/0 (0) | p>0.999 |
| ST1380 | Fluoroquinolones | 0/2 (0) | 0/0 (0) | p>0.999 |
| ST1380 | Sulfonamides | 0/2 (0) | 0/0 (0) | p>0.999 |
| ST1380 | Tetracyclines | 0/2 (0) | 0/0 (0) | p>0.999 |
| ST1380 | ESBL | 0/2 (0) | 0/0 (0) | p>0.999 |
| ST1380 | MDR | 0/2 (0) | 0/0 (0) | p>0.999 |
| ST14 | Aminoglycosides | 0/0 (0) | 6/7 (85.714) | p>0.999 |
| ST14 | Penicillins | 0/0 (0) | 7/7 (100) | p>0.999 |
| ST14 | Cephalosporins | 0/0 (0) | 7/7 (100) | p>0.999 |
| ST14 | Carbapenems | 0/0 (0) | 0/7 (0) | p>0.999 |
| ST14 | Polymyxin | 0/0 (0) | 1/7 (14.286) | p>0.999 |
| ST14 | Fluoroquinolones | 0/0 (0) | 0/7 (0) | p>0.999 |
| ST14 | Sulfonamides | 0/0 (0) | 7/7 (100) | p>0.999 |
| ST14 | Tetracyclines | 0/0 (0) | 0/7 (0) | p>0.999 |
| ST14 | ESBL | 0/0 (0) | 7/7 (100) | p>0.999 |
| ST14 | MDR | 0/0 (0) | 7/7 (100) | p>0.999 |
| ST1401 | Aminoglycosides | 0/1 (0) | 0/0 (0) | p>0.999 |
| ST1401 | Penicillins | 1/1 (100) | 0/0 (0) | p>0.999 |
| ST1401 | Cephalosporins | 0/1 (0) | 0/0 (0) | p>0.999 |
| ST1401 | Carbapenems | 0/1 (0) | 0/0 (0) | p>0.999 |
| ST1401 | Polymyxin | 0/1 (0) | 0/0 (0) | p>0.999 |
| ST1401 | Fluoroquinolones | 0/1 (0) | 0/0 (0) | p>0.999 |
| ST1401 | Sulfonamides | 0/1 (0) | 0/0 (0) | p>0.999 |
| ST1401 | Tetracyclines | 0/1 (0) | 0/0 (0) | p>0.999 |
| ST1401 | ESBL | 0/1 (0) | 0/0 (0) | p>0.999 |
| ST1401 | MDR | 0/1 (0) | 0/0 (0) | p>0.999 |
| ST1414 | Aminoglycosides | 6/6 (100) | 1/1 (100) | p>0.999 |
| ST1414 | Penicillins | 6/6 (100) | 1/1 (100) | p>0.999 |
| ST1414 | Cephalosporins | 6/6 (100) | 1/1 (100) | p>0.999 |
| ST1414 | Carbapenems | 0/6 (0) | 0/1 (0) | p>0.999 |
| ST1414 | Polymyxin | 0/6 (0) | 0/1 (0) | p>0.999 |
| ST1414 | Fluoroquinolones | 6/6 (100) | 1/1 (100) | p>0.999 |
| ST1414 | Sulfonamides | 6/6 (100) | 1/1 (100) | p>0.999 |
| ST1414 | Tetracyclines | 0/6 (0) | 0/1 (0) | p>0.999 |
| ST1414 | ESBL | 6/6 (100) | 1/1 (100) | p>0.999 |
| ST1414 | MDR | 6/6 (100) | 1/1 (100) | p>0.999 |
| ST1429 | Aminoglycosides | 0/1 (0) | 0/0 (0) | p>0.999 |
| ST1429 | Penicillins | 1/1 (100) | 0/0 (0) | p>0.999 |
| ST1429 | Cephalosporins | 0/1 (0) | 0/0 (0) | p>0.999 |
| ST1429 | Carbapenems | 0/1 (0) | 0/0 (0) | p>0.999 |
| ST1429 | Polymyxin | 0/1 (0) | 0/0 (0) | p>0.999 |
| ST1429 | Fluoroquinolones | 0/1 (0) | 0/0 (0) | p>0.999 |
| ST1429 | Sulfonamides | 0/1 (0) | 0/0 (0) | p>0.999 |
| ST1429 | Tetracyclines | 0/1 (0) | 0/0 (0) | p>0.999 |
| ST1429 | ESBL | 0/1 (0) | 0/0 (0) | p>0.999 |
| ST1429 | MDR | 0/1 (0) | 0/0 (0) | p>0.999 |
| ST15 | Aminoglycosides | 0/3 (0) | 1/1 (100) | p=0.25 |
| ST15 | Penicillins | 3/3 (100) | 1/1 (100) | p>0.999 |
| ST15 | Cephalosporins | 0/3 (0) | 1/1 (100) | p=0.25 |
| ST15 | Carbapenems | 0/3 (0) | 0/1 (0) | p>0.999 |
| ST15 | Polymyxin | 0/3 (0) | 0/1 (0) | p>0.999 |
| ST15 | Fluoroquinolones | 0/3 (0) | 1/1 (100) | p=0.25 |
| ST15 | Sulfonamides | 3/3 (100) | 1/1 (100) | p>0.999 |
| ST15 | Tetracyclines | 0/3 (0) | 1/1 (100) | p=0.25 |
| ST15 | ESBL | 0/3 (0) | 1/1 (100) | p=0.25 |
| ST15 | MDR | 0/3 (0) | 1/1 (100) | p=0.25 |
| ST152 | Aminoglycosides | 0/0 (0) | 4/5 (80) | p>0.999 |
| ST152 | Penicillins | 0/0 (0) | 5/5 (100) | p>0.999 |
| ST152 | Cephalosporins | 0/0 (0) | 4/5 (80) | p>0.999 |
| ST152 | Carbapenems | 0/0 (0) | 2/5 (40) | p>0.999 |
| ST152 | Polymyxin | 0/0 (0) | 0/5 (0) | p>0.999 |
| ST152 | Fluoroquinolones | 0/0 (0) | 4/5 (80) | p>0.999 |
| ST152 | Sulfonamides | 0/0 (0) | 4/5 (80) | p>0.999 |
| ST152 | Tetracyclines | 0/0 (0) | 2/5 (40) | p>0.999 |
| ST152 | ESBL | 0/0 (0) | 4/5 (80) | p>0.999 |
| ST152 | MDR | 0/0 (0) | 4/5 (80) | p>0.999 |
| ST1552 | Aminoglycosides | 0/0 (0) | 2/2 (100) | p>0.999 |
| ST1552 | Penicillins | 0/0 (0) | 2/2 (100) | p>0.999 |
| ST1552 | Cephalosporins | 0/0 (0) | 0/2 (0) | p>0.999 |
| ST1552 | Carbapenems | 0/0 (0) | 0/2 (0) | p>0.999 |
| ST1552 | Polymyxin | 0/0 (0) | 0/2 (0) | p>0.999 |
| ST1552 | Fluoroquinolones | 0/0 (0) | 0/2 (0) | p>0.999 |
| ST1552 | Sulfonamides | 0/0 (0) | 2/2 (100) | p>0.999 |
| ST1552 | Tetracyclines | 0/0 (0) | 0/2 (0) | p>0.999 |
| ST1552 | ESBL | 0/0 (0) | 0/2 (0) | p>0.999 |
| ST1552 | MDR | 0/0 (0) | 2/2 (100) | p>0.999 |
| ST163 | Aminoglycosides | 0/1 (0) | 0/0 (0) | p>0.999 |
| ST163 | Penicillins | 1/1 (100) | 0/0 (0) | p>0.999 |
| ST163 | Cephalosporins | 0/1 (0) | 0/0 (0) | p>0.999 |
| ST163 | Carbapenems | 0/1 (0) | 0/0 (0) | p>0.999 |
| ST163 | Polymyxin | 0/1 (0) | 0/0 (0) | p>0.999 |
| ST163 | Fluoroquinolones | 0/1 (0) | 0/0 (0) | p>0.999 |
| ST163 | Sulfonamides | 0/1 (0) | 0/0 (0) | p>0.999 |
| ST163 | Tetracyclines | 0/1 (0) | 0/0 (0) | p>0.999 |
| ST163 | ESBL | 0/1 (0) | 0/0 (0) | p>0.999 |
| ST163 | MDR | 0/1 (0) | 0/0 (0) | p>0.999 |
| ST1694 | Aminoglycosides | 0/1 (0) | 0/0 (0) | p>0.999 |
| ST1694 | Penicillins | 1/1 (100) | 0/0 (0) | p>0.999 |
| ST1694 | Cephalosporins | 0/1 (0) | 0/0 (0) | p>0.999 |
| ST1694 | Carbapenems | 0/1 (0) | 0/0 (0) | p>0.999 |
| ST1694 | Polymyxin | 0/1 (0) | 0/0 (0) | p>0.999 |
| ST1694 | Fluoroquinolones | 0/1 (0) | 0/0 (0) | p>0.999 |
| ST1694 | Sulfonamides | 0/1 (0) | 0/0 (0) | p>0.999 |
| ST1694 | Tetracyclines | 0/1 (0) | 0/0 (0) | p>0.999 |
| ST1694 | ESBL | 0/1 (0) | 0/0 (0) | p>0.999 |
| ST1694 | MDR | 0/1 (0) | 0/0 (0) | p>0.999 |
| ST17 | Aminoglycosides | 18/21 (85.714) | 19/19 (100) | p=0.233 |
| ST17 | Penicillins | 21/21 (100) | 19/19 (100) | p>0.999 |
| ST17 | Cephalosporins | 14/21 (66.667) | 19/19 (100) | p=0.009 |
| ST17 | Carbapenems | 17/21 (80.952) | 6/19 (31.579) | p=0.003 |
| ST17 | Polymyxin | 0/21 (0) | 0/19 (0) | p>0.999 |
| ST17 | Fluoroquinolones | 15/21 (71.429) | 4/19 (21.053) | p=0.002 |
| ST17 | Sulfonamides | 15/21 (71.429) | 16/19 (84.211) | p=0.457 |
| ST17 | Tetracyclines | 1/21 (4.762) | 0/19 (0) | p>0.999 |
| ST17 | ESBL | 14/21 (66.667) | 19/19 (100) | p=0.009 |
| ST17 | MDR | 18/21 (85.714) | 19/19 (100) | p=0.233 |
| ST1873 | Aminoglycosides | 0/0 (0) | 0/2 (0) | p>0.999 |
| ST1873 | Penicillins | 0/0 (0) | 2/2 (100) | p>0.999 |
| ST1873 | Cephalosporins | 0/0 (0) | 0/2 (0) | p>0.999 |
| ST1873 | Carbapenems | 0/0 (0) | 0/2 (0) | p>0.999 |
| ST1873 | Polymyxin | 0/0 (0) | 0/2 (0) | p>0.999 |
| ST1873 | Fluoroquinolones | 0/0 (0) | 0/2 (0) | p>0.999 |
| ST1873 | Sulfonamides | 0/0 (0) | 0/2 (0) | p>0.999 |
| ST1873 | Tetracyclines | 0/0 (0) | 0/2 (0) | p>0.999 |
| ST1873 | ESBL | 0/0 (0) | 0/2 (0) | p>0.999 |
| ST1873 | MDR | 0/0 (0) | 0/2 (0) | p>0.999 |
| ST193 | Aminoglycosides | 0/0 (0) | 1/1 (100) | p>0.999 |
| ST193 | Penicillins | 0/0 (0) | 1/1 (100) | p>0.999 |
| ST193 | Cephalosporins | 0/0 (0) | 1/1 (100) | p>0.999 |
| ST193 | Carbapenems | 0/0 (0) | 0/1 (0) | p>0.999 |
| ST193 | Polymyxin | 0/0 (0) | 0/1 (0) | p>0.999 |
| ST193 | Fluoroquinolones | 0/0 (0) | 1/1 (100) | p>0.999 |
| ST193 | Sulfonamides | 0/0 (0) | 1/1 (100) | p>0.999 |
| ST193 | Tetracyclines | 0/0 (0) | 1/1 (100) | p>0.999 |
| ST193 | ESBL | 0/0 (0) | 1/1 (100) | p>0.999 |
| ST193 | MDR | 0/0 (0) | 1/1 (100) | p>0.999 |
| ST1999 | Aminoglycosides | 0/1 (0) | 0/0 (0) | p>0.999 |
| ST1999 | Penicillins | 1/1 (100) | 0/0 (0) | p>0.999 |
| ST1999 | Cephalosporins | 0/1 (0) | 0/0 (0) | p>0.999 |
| ST1999 | Carbapenems | 0/1 (0) | 0/0 (0) | p>0.999 |
| ST1999 | Polymyxin | 0/1 (0) | 0/0 (0) | p>0.999 |
| ST1999 | Fluoroquinolones | 0/1 (0) | 0/0 (0) | p>0.999 |
| ST1999 | Sulfonamides | 0/1 (0) | 0/0 (0) | p>0.999 |
| ST1999 | Tetracyclines | 0/1 (0) | 0/0 (0) | p>0.999 |
| ST1999 | ESBL | 0/1 (0) | 0/0 (0) | p>0.999 |
| ST1999 | MDR | 0/1 (0) | 0/0 (0) | p>0.999 |
| ST20 | Aminoglycosides | 0/1 (0) | 0/0 (0) | p>0.999 |
| ST20 | Penicillins | 1/1 (100) | 0/0 (0) | p>0.999 |
| ST20 | Cephalosporins | 0/1 (0) | 0/0 (0) | p>0.999 |
| ST20 | Carbapenems | 0/1 (0) | 0/0 (0) | p>0.999 |
| ST20 | Polymyxin | 1/1 (100) | 0/0 (0) | p>0.999 |
| ST20 | Fluoroquinolones | 0/1 (0) | 0/0 (0) | p>0.999 |
| ST20 | Sulfonamides | 0/1 (0) | 0/0 (0) | p>0.999 |
| ST20 | Tetracyclines | 0/1 (0) | 0/0 (0) | p>0.999 |
| ST20 | ESBL | 0/1 (0) | 0/0 (0) | p>0.999 |
| ST20 | MDR | 0/1 (0) | 0/0 (0) | p>0.999 |
| ST2039 | Aminoglycosides | 0/0 (0) | 0/1 (0) | p>0.999 |
| ST2039 | Penicillins | 0/0 (0) | 1/1 (100) | p>0.999 |
| ST2039 | Cephalosporins | 0/0 (0) | 0/1 (0) | p>0.999 |
| ST2039 | Carbapenems | 0/0 (0) | 0/1 (0) | p>0.999 |
| ST2039 | Polymyxin | 0/0 (0) | 0/1 (0) | p>0.999 |
| ST2039 | Fluoroquinolones | 0/0 (0) | 0/1 (0) | p>0.999 |
| ST2039 | Sulfonamides | 0/0 (0) | 0/1 (0) | p>0.999 |
| ST2039 | Tetracyclines | 0/0 (0) | 0/1 (0) | p>0.999 |
| ST2039 | ESBL | 0/0 (0) | 0/1 (0) | p>0.999 |
| ST2039 | MDR | 0/0 (0) | 0/1 (0) | p>0.999 |
| ST22 | Aminoglycosides | 1/2 (50) | 0/0 (0) | p>0.999 |
| ST22 | Penicillins | 2/2 (100) | 0/0 (0) | p>0.999 |
| ST22 | Cephalosporins | 0/2 (0) | 0/0 (0) | p>0.999 |
| ST22 | Carbapenems | 0/2 (0) | 0/0 (0) | p>0.999 |
| ST22 | Polymyxin | 0/2 (0) | 0/0 (0) | p>0.999 |
| ST22 | Fluoroquinolones | 1/2 (50) | 0/0 (0) | p>0.999 |
| ST22 | Sulfonamides | 1/2 (50) | 0/0 (0) | p>0.999 |
| ST22 | Tetracyclines | 0/2 (0) | 0/0 (0) | p>0.999 |
| ST22 | ESBL | 0/2 (0) | 0/0 (0) | p>0.999 |
| ST22 | MDR | 1/2 (50) | 0/0 (0) | p>0.999 |
| ST231 | Aminoglycosides | 0/0 (0) | 1/1 (100) | p>0.999 |
| ST231 | Penicillins | 0/0 (0) | 1/1 (100) | p>0.999 |
| ST231 | Cephalosporins | 0/0 (0) | 1/1 (100) | p>0.999 |
| ST231 | Carbapenems | 0/0 (0) | 1/1 (100) | p>0.999 |
| ST231 | Polymyxin | 0/0 (0) | 0/1 (0) | p>0.999 |
| ST231 | Fluoroquinolones | 0/0 (0) | 1/1 (100) | p>0.999 |
| ST231 | Sulfonamides | 0/0 (0) | 1/1 (100) | p>0.999 |
| ST231 | Tetracyclines | 0/0 (0) | 0/1 (0) | p>0.999 |
| ST231 | ESBL | 0/0 (0) | 1/1 (100) | p>0.999 |
| ST231 | MDR | 0/0 (0) | 1/1 (100) | p>0.999 |
| ST2441 | Aminoglycosides | 0/0 (0) | 0/1 (0) | p>0.999 |
| ST2441 | Penicillins | 0/0 (0) | 1/1 (100) | p>0.999 |
| ST2441 | Cephalosporins | 0/0 (0) | 0/1 (0) | p>0.999 |
| ST2441 | Carbapenems | 0/0 (0) | 0/1 (0) | p>0.999 |
| ST2441 | Polymyxin | 0/0 (0) | 0/1 (0) | p>0.999 |
| ST2441 | Fluoroquinolones | 0/0 (0) | 0/1 (0) | p>0.999 |
| ST2441 | Sulfonamides | 0/0 (0) | 0/1 (0) | p>0.999 |
| ST2441 | Tetracyclines | 0/0 (0) | 0/1 (0) | p>0.999 |
| ST2441 | ESBL | 0/0 (0) | 0/1 (0) | p>0.999 |
| ST2441 | MDR | 0/0 (0) | 0/1 (0) | p>0.999 |
| ST25 | Aminoglycosides | 0/0 (0) | 2/2 (100) | p>0.999 |
| ST25 | Penicillins | 0/0 (0) | 1/2 (50) | p>0.999 |
| ST25 | Cephalosporins | 0/0 (0) | 1/2 (50) | p>0.999 |
| ST25 | Carbapenems | 0/0 (0) | 1/2 (50) | p>0.999 |
| ST25 | Polymyxin | 0/0 (0) | 0/2 (0) | p>0.999 |
| ST25 | Fluoroquinolones | 0/0 (0) | 0/2 (0) | p>0.999 |
| ST25 | Sulfonamides | 0/0 (0) | 2/2 (100) | p>0.999 |
| ST25 | Tetracyclines | 0/0 (0) | 0/2 (0) | p>0.999 |
| ST25 | ESBL | 0/0 (0) | 1/2 (50) | p>0.999 |
| ST25 | MDR | 0/0 (0) | 2/2 (100) | p>0.999 |
| ST252 | Aminoglycosides | 1/1 (100) | 1/1 (100) | p>0.999 |
| ST252 | Penicillins | 1/1 (100) | 1/1 (100) | p>0.999 |
| ST252 | Cephalosporins | 0/1 (0) | 1/1 (100) | p>0.999 |
| ST252 | Carbapenems | 1/1 (100) | 0/1 (0) | p>0.999 |
| ST252 | Polymyxin | 0/1 (0) | 0/1 (0) | p>0.999 |
| ST252 | Fluoroquinolones | 1/1 (100) | 0/1 (0) | p>0.999 |
| ST252 | Sulfonamides | 1/1 (100) | 1/1 (100) | p>0.999 |
| ST252 | Tetracyclines | 0/1 (0) | 1/1 (100) | p>0.999 |
| ST252 | ESBL | 0/1 (0) | 1/1 (100) | p>0.999 |
| ST252 | MDR | 1/1 (100) | 1/1 (100) | p>0.999 |
| ST307 | Aminoglycosides | 8/8 (100) | 16/17 (94.118) | p>0.999 |
| ST307 | Penicillins | 8/8 (100) | 17/17 (100) | p>0.999 |
| ST307 | Cephalosporins | 7/8 (87.5) | 16/17 (94.118) | p>0.999 |
| ST307 | Carbapenems | 0/8 (0) | 10/17 (58.824) | p-0.008 |
| ST307 | Fluoroquinolones | 8/8 (100) | 17/17 (100) | p>0.999 |
| ST307 | Sulfonamides | 7/8 (87.5) | 16/17 (94.118) | p>0.999 |
| ST307 | Tetracyclines | 0/8 (0) | 8/17 (47.059) | p=0.026 |
| ST307 | ESBL | 7/8 (87.5) | 16/17 (94.12) | p>0.999 |
| ST307 | MDR | 8/8 (100) | 17/17 (100) | p>0.999 |
| ST309 | Aminoglycosides | 0/1 (0) | 0/0 (0) | p>0.999 |
| ST309 | Penicillins | 1/1 (100) | 0/0 (0) | p>0.999 |
| ST309 | Cephalosporins | 0/1 (0) | 0/0 (0) | p>0.999 |
| ST309 | Carbapenems | 0/1 (0) | 0/0 (0) | p>0.999 |
| ST309 | Polymyxin | 0/1 (0) | 0/0 (0) | p>0.999 |
| ST309 | Fluoroquinolones | 0/1 (0) | 0/0 (0) | p>0.999 |
| ST309 | Sulfonamides | 0/1 (0) | 0/0 (0) | p>0.999 |
| ST309 | Tetracyclines | 0/1 (0) | 0/0 (0) | p>0.999 |
| ST309 | ESBL | 0/1 (0) | 0/0 (0) | p>0.999 |
| ST309 | MDR | 0/1 (0) | 0/0 (0) | p>0.999 |
| ST336 | Aminoglycosides | 0/0 (0) | 1/1 (100) | p>0.999 |
| ST336 | Penicillins | 0/0 (0) | 1/1 (100) | p>0.999 |
| ST336 | Cephalosporins | 0/0 (0) | 1/1 (100) | p>0.999 |
| ST336 | Carbapenems | 0/0 (0) | 0/1 (0) | p>0.999 |
| ST336 | Polymyxin | 0/0 (0) | 0/1 (0) | p>0.999 |
| ST336 | Fluoroquinolones | 0/0 (0) | 0/1 (0) | p>0.999 |
| ST336 | Sulfonamides | 0/0 (0) | 1/1 (100) | p>0.999 |
| ST336 | Tetracyclines | 0/0 (0) | 0/1 (0) | p>0.999 |
| ST336 | ESBL | 0/0 (0) | 1/1 (100) | p>0.999 |
| ST336 | MDR | 0/0 (0) | 1/1 (100) | p>0.999 |
| ST34 | Aminoglycosides | 0/1 (0) | 0/0 (0) | p>0.999 |
| ST34 | Penicillins | 1/1 (100) | 0/0 (0) | p>0.999 |
| ST34 | Cephalosporins | 0/1 (0) | 0/0 (0) | p>0.999 |
| ST34 | Carbapenems | 0/1 (0) | 0/0 (0) | p>0.999 |
| ST34 | Polymyxin | 0/1 (0) | 0/0 (0) | p>0.999 |
| ST34 | Fluoroquinolones | 0/1 (0) | 0/0 (0) | p>0.999 |
| ST34 | Sulfonamides | 0/1 (0) | 0/0 (0) | p>0.999 |
| ST34 | Tetracyclines | 0/1 (0) | 0/0 (0) | p>0.999 |
| ST34 | ESBL | 0/1 (0) | 0/0 (0) | p>0.999 |
| ST34 | MDR | 0/1 (0) | 0/0 (0) | p>0.999 |
| ST35 | Aminoglycosides | 0/1 (0) | 1/1 (100) | p>0.999 |
| ST35 | Penicillins | 1/1 (100) | 1/1 (100) | p>0.999 |
| ST35 | Cephalosporins | 0/1 (0) | 0/1 (0) | p>0.999 |
| ST35 | Carbapenems | 0/1 (0) | 0/1 (0) | p>0.999 |
| ST35 | Polymyxin | 0/1 (0) | 0/1 (0) | p>0.999 |
| ST35 | Fluoroquinolones | 0/1 (0) | 0/1 (0) | p>0.999 |
| ST35 | Sulfonamides | 0/1 (0) | 1/1 (100) | p>0.999 |
| ST35 | Tetracyclines | 0/1 (0) | 0/1 (0) | p>0.999 |
| ST35 | ESBL | 0/1 (0) | 0/1 (0) | p>0.999 |
| ST35 | MDR | 0/1 (0) | 1/1 (100) | p>0.999 |
| ST353 | Aminoglycosides | 0/1 (0) | 4/4 (100) | p=0.2 |
| ST353 | Penicillins | 1/1 (100) | 4/4 (100) | p>0.999 |
| ST353 | Cephalosporins | 0/1 (0) | 4/4 (100) | p=0.2 |
| ST353 | Carbapenems | 0/1 (0) | 0/4 (0) | p>0.999 |
| ST353 | Polymyxin | 0/1 (0) | 0/4 (0) | p>0.999 |
| ST353 | Fluoroquinolones | 0/1 (0) | 4/4 (100) | p=0.2 |
| ST353 | Sulfonamides | 0/1 (0) | 4/4 (100) | p=0.2 |
| ST353 | Tetracyclines | 0/1 (0) | 4/4 (100) | p=0.2 |
| ST353 | ESBL | 0/1 (0) | 4/4 (100) | p=0.2 |
| ST353 | MDR | 0/1 (0) | 4/4 (100) | p=0.2 |
| ST3688 | Aminoglycosides | 0/1 (0) | 0/0 (0) | p>0.999 |
| ST3688 | Penicillins | 1/1 (100) | 0/0 (0) | p>0.999 |
| ST3688 | Cephalosporins | 0/1 (0) | 0/0 (0) | p>0.999 |
| ST3688 | Carbapenems | 0/1 (0) | 0/0 (0) | p>0.999 |
| ST3688 | Polymyxin | 0/1 (0) | 0/0 (0) | p>0.999 |
| ST3688 | Fluoroquinolones | 0/1 (0) | 0/0 (0) | p>0.999 |
| ST3688 | Sulfonamides | 0/1 (0) | 0/0 (0) | p>0.999 |
| ST3688 | Tetracyclines | 0/1 (0) | 0/0 (0) | p>0.999 |
| ST3688 | ESBL | 0/1 (0) | 0/0 (0) | p>0.999 |
| ST3688 | MDR | 0/1 (0) | 0/0 (0) | p>0.999 |
| ST37 | Aminoglycosides | 0/1 (0) | 0/0 (0) | p>0.999 |
| ST37 | Penicillins | 1/1 (100) | 0/0 (0) | p>0.999 |
| ST37 | Cephalosporins | 0/1 (0) | 0/0 (0) | p>0.999 |
| ST37 | Carbapenems | 0/1 (0) | 0/0 (0) | p>0.999 |
| ST37 | Polymyxin | 0/1 (0) | 0/0 (0) | p>0.999 |
| ST37 | Fluoroquinolones | 0/1 (0) | 0/0 (0) | p>0.999 |
| ST37 | Sulfonamides | 0/1 (0) | 0/0 (0) | p>0.999 |
| ST37 | Tetracyclines | 0/1 (0) | 0/0 (0) | p>0.999 |
| ST37 | ESBL | 0/1 (0) | 0/0 (0) | p>0.999 |
| ST37 | MDR | 0/1 (0) | 0/0 (0) | p>0.999 |
| ST39 | Aminoglycosides | 0/0 (0) | 23/23 (100) | p>0.999 |
| ST39 | Penicillins | 0/0 (0) | 23/23 (100) | p>0.999 |
| ST39 | Cephalosporins | 0/0 (0) | 23/23 (100) | p>0.999 |
| ST39 | Carbapenems | 0/0 (0) | 23/23 (100) | p>0.999 |
| ST39 | Fluoroquinolones | 0/0 (0) | 4/23 (17.39) | p>0.999 |
| ST39 | Sulfonamides | 0/0 (0) | 4/23 (17.39) | p>0.999 |
| ST39 | Tetracyclines | 0/0 (0) | 0/23 (0) | p>0.999 |
| ST39 | ESBL | 0/0 (0) | 23/23 (100) | p>0.999 |
| ST39 | MDR | 0/0 (0) | 23/23 (100) | p>0.999 |
| ST3985 | Aminoglycosides | 0/1 (0) | 0/0 (0) | p>0.999 |
| ST3985 | Penicillins | 1/1 (100) | 0/0 (0) | p>0.999 |
| ST3985 | Cephalosporins | 0/1 (0) | 0/0 (0) | p>0.999 |
| ST3985 | Carbapenems | 0/1 (0) | 0/0 (0) | p>0.999 |
| ST3985 | Polymyxin | 0/1 (0) | 0/0 (0) | p>0.999 |
| ST3985 | Fluoroquinolones | 0/1 (0) | 0/0 (0) | p>0.999 |
| ST3985 | Sulfonamides | 0/1 (0) | 0/0 (0) | p>0.999 |
| ST3985 | Tetracyclines | 0/1 (0) | 0/0 (0) | p>0.999 |
| ST3985 | ESBL | 0/1 (0) | 0/0 (0) | p>0.999 |
| ST3985 | MDR | 0/1 (0) | 0/0 (0) | p>0.999 |
| ST416 | Aminoglycosides | 0/1 (0) | 0/0 (0) | p>0.999 |
| ST416 | Penicillins | 1/1 (100) | 0/0 (0) | p>0.999 |
| ST416 | Cephalosporins | 0/1 (0) | 0/0 (0) | p>0.999 |
| ST416 | Carbapenems | 0/1 (0) | 0/0 (0) | p>0.999 |
| ST416 | Polymyxin | 0/1 (0) | 0/0 (0) | p>0.999 |
| ST416 | Fluoroquinolones | 0/1 (0) | 0/0 (0) | p>0.999 |
| ST416 | Sulfonamides | 0/1 (0) | 0/0 (0) | p>0.999 |
| ST416 | Tetracyclines | 0/1 (0) | 0/0 (0) | p>0.999 |
| ST416 | ESBL | 0/1 (0) | 0/0 (0) | p>0.999 |
| ST416 | MDR | 0/1 (0) | 0/0 (0) | p>0.999 |
| ST4291 | Aminoglycosides | 0/0 (0) | 2/2 (100) | p>0.999 |
| ST4291 | Penicillins | 0/0 (0) | 2/2 (100) | p>0.999 |
| ST4291 | Cephalosporins | 0/0 (0) | 2/2 (100) | p>0.999 |
| ST4291 | Carbapenems | 0/0 (0) | 2/2 (100) | p>0.999 |
| ST4291 | Polymyxin | 0/0 (0) | 0/2 (0) | p>0.999 |
| ST4291 | Fluoroquinolones | 0/0 (0) | 2/2 (100) | p>0.999 |
| ST4291 | Sulfonamides | 0/0 (0) | 2/2 (100) | p>0.999 |
| ST4291 | Tetracyclines | 0/0 (0) | 0/2 (0) | p>0.999 |
| ST4291 | ESBL | 0/0 (0) | 2/2 (100) | p>0.999 |
| ST4291 | MDR | 0/0 (0) | 2/2 (100) | p>0.999 |
| ST460 | Aminoglycosides | 0/1 (0) | 0/0 (0) | p>0.999 |
| ST460 | Penicillins | 1/1 (100) | 0/0 (0) | p>0.999 |
| ST460 | Cephalosporins | 0/1 (0) | 0/0 (0) | p>0.999 |
| ST460 | Carbapenems | 0/1 (0) | 0/0 (0) | p>0.999 |
| ST460 | Polymyxin | 0/1 (0) | 0/0 (0) | p>0.999 |
| ST460 | Fluoroquinolones | 0/1 (0) | 0/0 (0) | p>0.999 |
| ST460 | Sulfonamides | 0/1 (0) | 0/0 (0) | p>0.999 |
| ST460 | Tetracyclines | 0/1 (0) | 0/0 (0) | p>0.999 |
| ST460 | ESBL | 0/1 (0) | 0/0 (0) | p>0.999 |
| ST460 | MDR | 0/1 (0) | 0/0 (0) | p>0.999 |
| ST461 | Aminoglycosides | 0/1 (0) | 0/0 (0) | p>0.999 |
| ST461 | Penicillins | 1/1 (100) | 0/0 (0) | p>0.999 |
| ST461 | Cephalosporins | 0/1 (0) | 0/0 (0) | p>0.999 |
| ST461 | Carbapenems | 0/1 (0) | 0/0 (0) | p>0.999 |
| ST461 | Polymyxin | 0/1 (0) | 0/0 (0) | p>0.999 |
| ST461 | Fluoroquinolones | 0/1 (0) | 0/0 (0) | p>0.999 |
| ST461 | Sulfonamides | 0/1 (0) | 0/0 (0) | p>0.999 |
| ST461 | Tetracyclines | 0/1 (0) | 0/0 (0) | p>0.999 |
| ST461 | ESBL | 0/1 (0) | 0/0 (0) | p>0.999 |
| ST461 | MDR | 0/1 (0) | 0/0 (0) | p>0.999 |
| ST502 | Aminoglycosides | 0/4 (0) | 0/0 (0) | p>0.999 |
| ST502 | Penicillins | 4/4 (100) | 0/0 (0) | p>0.999 |
| ST502 | Cephalosporins | 0/4 (0) | 0/0 (0) | p>0.999 |
| ST502 | Carbapenems | 0/4 (0) | 0/0 (0) | p>0.999 |
| ST502 | Polymyxin | 0/4 (0) | 0/0 (0) | p>0.999 |
| ST502 | Fluoroquinolones | 0/4 (0) | 0/0 (0) | p>0.999 |
| ST502 | Sulfonamides | 0/4 (0) | 0/0 (0) | p>0.999 |
| ST502 | Tetracyclines | 0/4 (0) | 0/0 (0) | p>0.999 |
| ST502 | ESBL | 0/4 (0) | 0/0 (0) | p>0.999 |
| ST502 | MDR | 0/4 (0) | 0/0 (0) | p>0.999 |
| ST607 | Aminoglycosides | 0/0 (0) | 2/2 (100) | p>0.999 |
| ST607 | Penicillins | 0/0 (0) | 2/2 (100) | p>0.999 |
| ST607 | Cephalosporins | 0/0 (0) | 2/2 (100) | p>0.999 |
| ST607 | Carbapenems | 0/0 (0) | 0/2 (0) | p>0.999 |
| ST607 | Polymyxin | 0/0 (0) | 0/2 (0) | p>0.999 |
| ST607 | Fluoroquinolones | 0/0 (0) | 1/2 (50) | p>0.999 |
| ST607 | Sulfonamides | 0/0 (0) | 2/2 (100) | p>0.999 |
| ST607 | Tetracyclines | 0/0 (0) | 1/2 (50) | p>0.999 |
| ST607 | ESBL | 0/0 (0) | 2/2 (100) | p>0.999 |
| ST607 | MDR | 0/0 (0) | 2/2 (100) | p>0.999 |
| ST611 | Aminoglycosides | 0/1 (0) | 0/0 (0) | p>0.999 |
| ST611 | Penicillins | 1/1 (100) | 0/0 (0) | p>0.999 |
| ST611 | Cephalosporins | 0/1 (0) | 0/0 (0) | p>0.999 |
| ST611 | Carbapenems | 0/1 (0) | 0/0 (0) | p>0.999 |
| ST611 | Polymyxin | 0/1 (0) | 0/0 (0) | p>0.999 |
| ST611 | Fluoroquinolones | 0/1 (0) | 0/0 (0) | p>0.999 |
| ST611 | Sulfonamides | 0/1 (0) | 0/0 (0) | p>0.999 |
| ST611 | Tetracyclines | 0/1 (0) | 0/0 (0) | p>0.999 |
| ST611 | ESBL | 0/1 (0) | 0/0 (0) | p>0.999 |
| ST611 | MDR | 0/1 (0) | 0/0 (0) | p>0.999 |
| ST987 | Aminoglycosides | 0/0 (0) | 0/1 (0) | p>0.999 |
| ST987 | Penicillins | 0/0 (0) | 1/1 (100) | p>0.999 |
| ST987 | Cephalosporins | 0/0 (0) | 0/1 (0) | p>0.999 |
| ST987 | Carbapenems | 0/0 (0) | 0/1 (0) | p>0.999 |
| ST987 | Polymyxin | 0/0 (0) | 0/1 (0) | p>0.999 |
| ST987 | Fluoroquinolones | 0/0 (0) | 0/1 (0) | p>0.999 |
| ST987 | Sulfonamides | 0/0 (0) | 0/1 (0) | p>0.999 |
| ST987 | Tetracyclines | 0/0 (0) | 0/1 (0) | p>0.999 |
| ST987 | ESBL | 0/0 (0) | 0/1 (0) | p>0.999 |
| ST987 | MDR | 0/0 (0) | 0/1 (0) | p>0.999 |
| **O- loci, n (%)** |  |  |  |  |
| O1/O2v1 | Aminoglycosides | 30/42 (71.429) | 12/16 (75) | p>0.999 |
| O1/O2v1 | Penicillins | 42/42 (100) | 16/16 (100) | p>0.999 |
| O1/O2v1 | Cephalosporins | 28/42 (66.667) | 11/16 (68.75) | p>0.999 |
| O1/O2v1 | Carbapenems | 1/42 (2.381) | 0/16 (0) | p>0.999 |
| O1/O2v1 | Polymyxin | 2/42 (4.762) | 1/16 (6.25) | p>0.999 |
| O1/O2v1 | Fluoroquinolones | 28/42 (66.667) | 3/16 (18.75) | p=0.001 |
| O1/O2v1 | Sulfonamides | 32/42 (76.19) | 13/16 (81.25) | p>0.999 |
| O1/O2v1 | Tetracyclines | 22/42 (52.381) | 2/16 (12.5) | p=0.007 |
| O1/O2v1 | ESBL | 28/42 (66.667) | 11/16 (68.75) | p>0.999 |
| O1/O2v1 | MDR | 29/42 (69.048) | 13/16 (81.25) | p=0.514 |
| O1/O2v2 | Aminoglycosides | 13/19 (68.421) | 53/55 (96.364) | p=0.003 |
| O1/O2v2 | Penicillins | 19/19 (100) | 54/55 (98.182) | p>0.999 |
| O1/O2v2 | Cephalosporins | 8/19 (42.105) | 51/55 (92.727) | p<0.001 |
| O1/O2v2 | Carbapenems | 1/19 (5.263) | 17/55 (30.909) | p=0.03 |
| O1/O2v2 | Polymyxin | 0/19 (0) | 0/55 (0) | p>0.999 |
| O1/O2v2 | Fluoroquinolones | 10/19 (52.632) | 25/55 (45.455) | p=0.606 |
| O1/O2v2 | Sulfonamides | 12/19 (63.158) | 30/55 (54.545) | p=0.597 |
| O1/O2v2 | Tetracyclines | 2/19 (10.526) | 11/55 (20) | p=0.494 |
| O1/O2v2 | ESBL | 8/19 (42.105) | 51/55 (92.727) | p<0.001 |
| O1/O2v2 | MDR | 13/19 (68.421) | 54/55 (98.182) | p<0.001 |
| O12 | Aminoglycosides | 0/1 (0) | 0/0 (0) | p>0.999 |
| O12 | Penicillins | 1/1 (100) | 0/0 (0) | p>0.999 |
| O12 | Cephalosporins | 0/1 (0) | 0/0 (0) | p>0.999 |
| O12 | Carbapenems | 0/1 (0) | 0/0 (0) | p>0.999 |
| O12 | Polymyxin | 0/1 (0) | 0/0 (0) | p>0.999 |
| O12 | Fluoroquinolones | 0/1 (0) | 0/0 (0) | p>0.999 |
| O12 | Sulfonamides | 0/1 (0) | 0/0 (0) | p>0.999 |
| O12 | Tetracyclines | 0/1 (0) | 0/0 (0) | p>0.999 |
| O12 | ESBL | 0/1 (0) | 0/0 (0) | p>0.999 |
| O12 | MDR | 0/1 (0) | 0/0 (0) | p>0.999 |
| O3/O3a | Aminoglycosides | 0/1 (0) | 0/0 (0) | p>0.999 |
| O3/O3a | Penicillins | 0/1 (0) | 0/0 (0) | p>0.999 |
| O3/O3a | Cephalosporins | 0/1 (0) | 0/0 (0) | p>0.999 |
| O3/O3a | Carbapenems | 0/1 (0) | 0/0 (0) | p>0.999 |
| O3/O3a | Polymyxin | 0/1 (0) | 0/0 (0) | p>0.999 |
| O3/O3a | Fluoroquinolones | 0/1 (0) | 0/0 (0) | p>0.999 |
| O3/O3a | Sulfonamides | 0/1 (0) | 0/0 (0) | p>0.999 |
| O3/O3a | Tetracyclines | 0/1 (0) | 0/0 (0) | p>0.999 |
| O3/O3a | ESBL | 0/1 (0) | 0/0 (0) | p>0.999 |
| O3/O3a | MDR | 0/1 (0) | 0/0 (0) | p>0.999 |
| O3b | Aminoglycosides | 1/4 (25) | 4/5 (80) | p=0.206 |
| O3b | Penicillins | 4/4 (100) | 5/5 (100) | p>0.999 |
| O3b | Cephalosporins | 0/4 (0) | 4/5 (80) | p=0.048 |
| O3b | Carbapenems | 0/4 (0) | 0/5 (0) | p>0.999 |
| O3b | Polymyxin | 0/4 (0) | 0/5 (0) | p>0.999 |
| O3b | Fluoroquinolones | 0/4 (0) | 4/5 (80) | p=0.048 |
| O3b | Sulfonamides | 0/4 (0) | 4/5 (80) | p=0.048 |
| O3b | Tetracyclines | 0/4 (0) | 4/5 (80) | p=0.048 |
| O3b | ESBL | 0/4 (0) | 4/5 (80) | p=0.048 |
| O3b | MDR | 0/4 (0) | 4/5 (80) | p=0.048 |
| O4 | Aminoglycosides | 0/7 (0) | 4/5 (80) | p=0.01 |
| O4 | Penicillins | 7/7 (100) | 5/5 (100) | p>0.999 |
| O4 | Cephalosporins | 0/7 (0) | 4/5 (80) | p=0.01 |
| O4 | Carbapenems | 0/7 (0) | 2/5 (40) | p=0.152 |
| O4 | Polymyxin | 0/7 (0) | 0/5 (0) | p>0.999 |
| O4 | Fluoroquinolones | 0/7 (0) | 4/5 (80) | p=0.01 |
| O4 | Sulfonamides | 0/7 (0) | 4/5 (80) | p=0.01 |
| O4 | Tetracyclines | 0/7 (0) | 2/5 (40) | p=0.152 |
| O4 | ESBL | 0/7 (0) | 4/5 (80) | p=0.01 |
| O4 | MDR | 0/7 (0) | 4/5 (80) | p=0.01 |
| O5 | Aminoglycosides | 18/20 (90) | 17/17 (100) | p=0.489 |
| O5 | Penicillins | 20/20 (100) | 17/17 (100) | p>0.999 |
| O5 | Cephalosporins | 14/20 (70) | 17/17 (100) | p=0.022 |
| O5 | Carbapenems | 17/20 (85) | 6/17 (35.294) | p=0.003 |
| O5 | Polymyxin | 0/20 (0) | 0/17 (0) | p>0.999 |
| O5 | Fluoroquinolones | 15/20 (75) | 4/17 (23.529) | p=0.003 |
| O5 | Sulfonamides | 15/20 (75) | 17/17 (100) | p=0.05 |
| O5 | Tetracyclines | 1/20 (5) | 0/17 (0) | p>0.999 |
| O5 | ESBL | 14/20 (70) | 17/17 (100) | p=0.022 |
| O5 | MDR | 18/20 (90) | 17/17 (100) | p=0.489 |
| **O-type, n (%)** |  |  |  |  |
| O1ab | Aminoglycosides | 33/47 (70.213) | 43/45 (95.556) | p=0.002 |
| O1ab | Penicillins | 47/47 (100) | 45/45 (100) | p>0.999 |
| O1ab | Cephalosporins | 28/47 (59.574) | 40/45 (88.889) | p=0.002 |
| O1ab | Carbapenems | 2/47 (4.255) | 6/45 (13.333) | p=0.158 |
| O1ab | Polymyxin | 2/47 (4.255) | 1/46 (2.174) | p>0.999 |
| O1ab | Fluoroquinolones | 29/47 (61.702) | 10/45 (22.222) | p<0.001 |
| O1ab | Sulfonamides | 35/47 (74.468) | 23/45 (51.111) | p=0.053 |
| O1ab | Tetracyclines | 24/47 (51.064) | 5/45 (11.111) | p<0.001 |
| O1ab | ESBL | 28/47 (59.574) | 40/45 (88.889) | p=0.002 |
| O1ab | MDR | 32/47 (68.085) | 44/45 (97.778) | p<0.001 |
| O12 | Aminoglycosides | 0/1 (0) | 0/0 (0) | p>0.999 |
| O12 | Penicillins | 1/1 (100) | 0/0 (0) | p>0.999 |
| O12 | Cephalosporins | 0/1 (0) | 0/0 (0) | p>0.999 |
| O12 | Carbapenems | 0/1 (0) | 0/0 (0) | p>0.999 |
| O12 | Polymyxin | 0/1 (0) | 0/0 (0) | p>0.999 |
| O12 | Fluoroquinolones | 0/1 (0) | 0/0 (0) | p>0.999 |
| O12 | Sulfonamides | 0/1 (0) | 0/0 (0) | p>0.999 |
| O12 | Tetracyclines | 0/1 (0) | 0/0 (0) | p>0.999 |
| O12 | ESBL | 0/1 (0) | 0/0 (0) | p>0.999 |
| O12 | MDR | 0/1 (0) | 0/0 (0) | p>0.999 |
| O2a | Aminoglycosides | 0/3 (0) | 0/2 (0) | p>0.999 |
| O2a | Penicillins | 3/3 (100) | 2/2 (100) | p>0.999 |
| O2a | Cephalosporins | 0/3 (0) | 0/2 (0) | p>0.999 |
| O2a | Carbapenems | 0/3 (0) | 0/2 (0) | p>0.999 |
| O2a | Polymyxin | 0/3 (0) | 0/2 (0) | p>0.999 |
| O2a | Fluoroquinolones | 0/3 (0) | 0/2 (0) | p>0.999 |
| O2a | Sulfonamides | 0/3 (0) | 0/2 (0) | p>0.999 |
| O2a | Tetracyclines | 0/3 (0) | 0/2 (0) | p>0.999 |
| O2a | ESBL | 0/3 (0) | 0/2 (0) | p>0.999 |
| O2a | MDR | 0/3 (0) | 0/2 (0) | p>0.999 |
| O2afg | Aminoglycosides | 10/11 (90.909) | 22/24 (91.667) | p>0.999 |
| O2afg | Penicillins | 11/11 (100) | 23/24 (95.833) | p>0.999 |
| O2afg | Cephalosporins | 8/11 (72.727) | 22/24 (91.667) | p=0.3 |
| O2afg | Carbapenems | 0/11 (0) | 12/24 (50) | p=0.006 |
| O2afg | Polymyxin | 0/11 (0) | 0/24 (0) | p>0.999 |
| O2afg | Fluoroquinolones | 9/11 (81.818) | 19/24 (79.167) | p>0.999 |
| O2afg | Sulfonamides | 9/11 (81.818) | 23/24 (95.833) | p>0.999 |
| O2afg | Tetracyclines | 0/11 (0) | 8/24 (33.333) | p=0.034 |
| O2afg | ESBL | 8/11 (72.727) | 22/24 (91.667) | p=0.3 |
| O2afg | MDR | 10/11 (90.909) | 23/24 (95.833) | p>0.999 |
| O3/O3a | Aminoglycosides | 0/1 (0) | 0/0 (0) | p>0.999 |
| O3/O3a | Penicillins | 0/1 (0) | 0/0 (0) | p>0.999 |
| O3/O3a | Cephalosporins | 0/1 (0) | 0/0 (0) | p>0.999 |
| O3/O3a | Carbapenems | 0/1 (0) | 0/0 (0) | p>0.999 |
| O3/O3a | Polymyxin | 0/1 (0) | 0/0 (0) | p>0.999 |
| O3/O3a | Fluoroquinolones | 0/1 (0) | 0/0 (0) | p>0.999 |
| O3/O3a | Sulfonamides | 0/1 (0) | 0/0 (0) | p>0.999 |
| O3/O3a | Tetracyclines | 0/1 (0) | 0/0 (0) | p>0.999 |
| O3/O3a | ESBL | 0/1 (0) | 0/0 (0) | p>0.999 |
| O3/O3a | MDR | 0/1 (0) | 0/0 (0) | p>0.999 |
| O3b | Aminoglycosides | 1/4 (25) | 4/5 (80) | p=0.206 |
| O3b | Penicillins | 4/4 (100) | 5/5 (100) | p>0.999 |
| O3b | Cephalosporins | 0/4 (0) | 4/5 (80) | p=0.048 |
| O3b | Carbapenems | 0/4 (0) | 0/5 (0) | p>0.999 |
| O3b | Polymyxin | 0/4 (0) | 0/5 (0) | p>0.999 |
| O3b | Fluoroquinolones | 0/4 (0) | 4/5 (80) | p=0.048 |
| O3b | Sulfonamides | 0/4 (0) | 4/5 (80) | p=0.048 |
| O3b | Tetracyclines | 0/4 (0) | 4/5 (80) | p=0.048 |
| O3b | ESBL | 0/4 (0) | 4/5 (80) | p=0.048 |
| O3b | MDR | 0/4 (0) | 4/5 (80) | p=0.048 |
| O4 | Aminoglycosides | 0/7 (0) | 4/5 (80) | p=0.01 |
| O4 | Penicillins | 7/7 (100) | 5/5 (100) | p>0.999 |
| O4 | Cephalosporins | 0/7 (0) | 4/5 (80) | p=0.01 |
| O4 | Carbapenems | 0/7 (0) | 2/5 (40) | p=0.152 |
| O4 | Polymyxin | 0/7 (0) | 0/5 (0) | p>0.999 |
| O4 | Fluoroquinolones | 0/7 (0) | 4/5 (80) | p=0.01 |
| O4 | Sulfonamides | 0/7 (0) | 4/5 (80) | p=0.01 |
| O4 | Tetracyclines | 0/7 (0) | 2/5 (40) | p=0.152 |
| O4 | ESBL | 0/7 (0) | 4/5 (80) | p=0.01 |
| O4 | MDR | 0/7 (0) | 4/5 (80) | p=0.01 |
| O5 | Aminoglycosides | 18/20 (90) | 17/17 (100) | p=0.489 |
| O5 | Penicillins | 20/20 (100) | 17/17 (100) | p>0.999 |
| O5 | Cephalosporins | 14/20 (70) | 17/17 (100) | p=0.022 |
| O5 | Carbapenems | 17/20 (85) | 6/17 (35.294) | p=0.003 |
| O5 | Polymyxin | 0/20 (0) | 0/17 (0) | p>0.999 |
| O5 | Fluoroquinolones | 15/20 (75) | 4/17 (23.529) | p=0.003 |
| O5 | Sulfonamides | 15/20 (75) | 17/17 (100) | p=0.05 |
| O5 | Tetracyclines | 1/20 (5) | 0/17 (0) | p>0.999 |
| O5 | ESBL | 14/20 (70) | 17/17 (100) | p=0.022 |
| O5 | MDR | 18/20 (90) | 17/17 (100) | p=0.489 |
| **K-loci, n (%)** |  |  |  |  |
| KL10 | Aminoglycosides | 0/2 (0) | 0/0 (0) | p>0.999 |
| KL10 | Penicillins | 1/2 (50) | 0/0 (0) | p>0.999 |
| KL10 | Cephalosporins | 0/2 (0) | 0/0 (0) | p>0.999 |
| KL10 | Carbapenems | 0/2 (0) | 0/0 (0) | p>0.999 |
| KL10 | Polymyxin | 0/2 (0) | 0/0 (0) | p>0.999 |
| KL10 | Fluoroquinolones | 0/2 (0) | 0/0 (0) | p>0.999 |
| KL10 | Sulfonamides | 0/2 (0) | 0/0 (0) | p>0.999 |
| KL10 | Tetracyclines | 0/2 (0) | 0/0 (0) | p>0.999 |
| KL10 | ESBL | 0/2 (0) | 0/0 (0) | p>0.999 |
| KL10 | MDR | 0/2 (0) | 0/0 (0) | p>0.999 |
| KL102 | Aminoglycosides | 8/8 (100) | 18/19 (94.737) | p>0.999 |
| KL102 | Penicillins | 8/8 (100) | 19/19 (100) | p>0.999 |
| KL102 | Cephalosporins | 7/8 (87.5) | 18/19 (94.737) | P=0.068 |
| KL102 | Carbapenems | 0/8 (0) | 12/19 (63.158) | P=0.003 |
| KL102 | Polymyxin | 0/8 (0) | 0/19 (0) | p>0.999 |
| KL102 | Fluoroquinolones | 8/8 (100) | 19/19 (100) | p>0.999 |
| KL102 | Sulfonamides | 7/8 (87.5) | 17/19 (89.474) | P=0.513 |
| KL102 | Tetracyclines | 0/8 (0) | 8/19 (42.105) | p=0.061 |
| KL102 | ESBL | 7/8 (87.5) | 18/19 (94.737) | P=0.513 |
| KL102 | MDR | 8/8 (100) | 19/19 (100) | p>0.999 |
| KL103 | Aminoglycosides | 1/1 (100) | 0/0 (0) | p>0.999 |
| KL103 | Penicillins | 1/1 (100) | 0/0 (0) | p>0.999 |
| KL103 | Cephalosporins | 0/1 (0) | 0/0 (0) | p>0.999 |
| KL103 | Carbapenems | 0/1 (0) | 0/0 (0) | p>0.999 |
| KL103 | Polymyxin | 0/1 (0) | 0/0 (0) | p>0.999 |
| KL103 | Fluoroquinolones | 0/1 (0) | 0/0 (0) | p>0.999 |
| KL103 | Sulfonamides | 0/1 (0) | 0/0 (0) | p>0.999 |
| KL103 | Tetracyclines | 0/1 (0) | 0/0 (0) | p>0.999 |
| KL103 | ESBL | 0/1 (0) | 0/0 (0) | p>0.999 |
| KL103 | MDR | 0/1 (0) | 0/0 (0) | p>0.999 |
| KL106 | Aminoglycosides | 0/1 (0) | 0/0 (0) | p>0.999 |
| KL106 | Penicillins | 1/1 (100) | 0/0 (0) | p>0.999 |
| KL106 | Cephalosporins | 0/1 (0) | 0/0 (0) | p>0.999 |
| KL106 | Carbapenems | 0/1 (0) | 0/0 (0) | p>0.999 |
| KL106 | Polymyxin | 0/1 (0) | 0/0 (0) | p>0.999 |
| KL106 | Fluoroquinolones | 0/1 (0) | 0/0 (0) | p>0.999 |
| KL106 | Sulfonamides | 0/1 (0) | 0/0 (0) | p>0.999 |
| KL106 | Tetracyclines | 0/1 (0) | 0/0 (0) | p>0.999 |
| KL106 | ESBL | 0/1 (0) | 0/0 (0) | p>0.999 |
| KL106 | MDR | 0/1 (0) | 0/0 (0) | p>0.999 |
| KL108 | Aminoglycosides | 0/0 (0) | 1/1 (100) | p>0.999 |
| KL108 | Penicillins | 0/0 (0) | 1/1 (100) | p>0.999 |
| KL108 | Cephalosporins | 0/0 (0) | 0/1 (0) | p>0.999 |
| KL108 | Carbapenems | 0/0 (0) | 0/1 (0) | p>0.999 |
| KL108 | Polymyxin | 0/0 (0) | 0/1 (0) | p>0.999 |
| KL108 | Fluoroquinolones | 0/0 (0) | 0/1 (0) | p>0.999 |
| KL108 | Sulfonamides | 0/0 (0) | 1/1 (100) | p>0.999 |
| KL108 | Tetracyclines | 0/0 (0) | 0/1 (0) | p>0.999 |
| KL108 | ESBL | 0/0 (0) | 0/1 (0) | p>0.999 |
| KL108 | MDR | 0/0 (0) | 1/1 (100) | p>0.999 |
| KL110 | Aminoglycosides | 1/2 (50) | 4/4 (100) | p=0.333 |
| KL110 | Penicillins | 2/2 (100) | 4/4 (100) | p>0.999 |
| KL110 | Cephalosporins | 0/2 (0) | 4/4 (100) | p=0.067 |
| KL110 | Carbapenems | 0/2 (0) | 0/4 (0) | p>0.999 |
| KL110 | Polymyxin | 0/2 (0) | 0/4 (0) | p>0.999 |
| KL110 | Fluoroquinolones | 0/2 (0) | 4/4 (100) | p=0.067 |
| KL110 | Sulfonamides | 0/2 (0) | 4/4 (100) | p=0.067 |
| KL110 | Tetracyclines | 0/2 (0) | 4/4 (100) | p=0.067 |
| KL110 | ESBL | 0/2 (0) | 4/4 (100) | p=0.067 |
| KL110 | MDR | 0/2 (0) | 4/4 (100) | p=0.067 |
| KL112 | Aminoglycosides | 0/0 (0) | 1/1 (100) | p>0.999 |
| KL112 | Penicillins | 0/0 (0) | 1/1 (100) | p>0.999 |
| KL112 | Cephalosporins | 0/0 (0) | 1/1 (100) | p>0.999 |
| KL112 | Carbapenems | 0/0 (0) | 0/1 (0) | p>0.999 |
| KL112 | Polymyxin | 0/0 (0) | 0/1 (0) | p>0.999 |
| KL112 | Fluoroquinolones | 0/0 (0) | 1/1 (100) | p>0.999 |
| KL112 | Sulfonamides | 0/0 (0) | 1/1 (100) | p>0.999 |
| KL112 | Tetracyclines | 0/0 (0) | 1/1 (100) | p>0.999 |
| KL112 | ESBL | 0/0 (0) | 1/1 (100) | p>0.999 |
| KL112 | MDR | 0/0 (0) | 1/1 (100) | p>0.999 |
| KL116 | Aminoglycosides | 2/2 (100) | 0/0 (0) | p>0.999 |
| KL116 | Penicillins | 2/2 (100) | 0/0 (0) | p>0.999 |
| KL116 | Cephalosporins | 2/2 (100) | 0/0 (0) | p>0.999 |
| KL116 | Carbapenems | 0/2 (0) | 0/0 (0) | p>0.999 |
| KL116 | Polymyxin | 0/2 (0) | 0/0 (0) | p>0.999 |
| KL116 | Fluoroquinolones | 2/2 (100) | 0/0 (0) | p>0.999 |
| KL116 | Sulfonamides | 2/2 (100) | 0/0 (0) | p>0.999 |
| KL116 | Tetracyclines | 0/2 (0) | 0/0 (0) | p>0.999 |
| KL116 | ESBL | 2/2 (100) | 0/0 (0) | p>0.999 |
| KL116 | MDR | 2/2 (100) | 0/0 (0) | p>0.999 |
| KL12 | Aminoglycosides | 0/1 (0) | 0/0 (0) | p>0.999 |
| KL12 | Penicillins | 1/1 (100) | 0/0 (0) | p>0.999 |
| KL12 | Cephalosporins | 0/1 (0) | 0/0 (0) | p>0.999 |
| KL12 | Carbapenems | 0/1 (0) | 0/0 (0) | p>0.999 |
| KL12 | Polymyxin | 0/1 (0) | 0/0 (0) | p>0.999 |
| KL12 | Fluoroquinolones | 0/1 (0) | 0/0 (0) | p>0.999 |
| KL12 | Sulfonamides | 0/1 (0) | 0/0 (0) | p>0.999 |
| KL12 | Tetracyclines | 0/1 (0) | 0/0 (0) | p>0.999 |
| KL12 | ESBL | 0/1 (0) | 0/0 (0) | p>0.999 |
| KL12 | MDR | 0/1 (0) | 0/0 (0) | p>0.999 |
| KL125 | Aminoglycosides | 0/1 (0) | 0/0 (0) | p>0.999 |
| KL125 | Penicillins | 1/1 (100) | 0/0 (0) | p>0.999 |
| KL125 | Cephalosporins | 0/1 (0) | 0/0 (0) | p>0.999 |
| KL125 | Carbapenems | 0/1 (0) | 0/0 (0) | p>0.999 |
| KL125 | Polymyxin | 0/1 (0) | 0/0 (0) | p>0.999 |
| KL125 | Fluoroquinolones | 0/1 (0) | 0/0 (0) | p>0.999 |
| KL125 | Sulfonamides | 0/1 (0) | 0/0 (0) | p>0.999 |
| KL125 | Tetracyclines | 0/1 (0) | 0/0 (0) | p>0.999 |
| KL125 | ESBL | 0/1 (0) | 0/0 (0) | p>0.999 |
| KL125 | MDR | 0/1 (0) | 0/0 (0) | p>0.999 |
| KL128 | Aminoglycosides | 0/1 (0) | 0/0 (0) | p>0.999 |
| KL128 | Penicillins | 1/1 (100) | 0/0 (0) | p>0.999 |
| KL128 | Cephalosporins | 0/1 (0) | 0/0 (0) | p>0.999 |
| KL128 | Carbapenems | 0/1 (0) | 0/0 (0) | p>0.999 |
| KL128 | Polymyxin | 0/1 (0) | 0/0 (0) | p>0.999 |
| KL128 | Fluoroquinolones | 0/1 (0) | 0/0 (0) | p>0.999 |
| KL128 | Sulfonamides | 0/1 (0) | 0/0 (0) | p>0.999 |
| KL128 | Tetracyclines | 0/1 (0) | 0/0 (0) | p>0.999 |
| KL128 | ESBL | 0/1 (0) | 0/0 (0) | p>0.999 |
| KL128 | MDR | 0/1 (0) | 0/0 (0) | p>0.999 |
| KL140 | Aminoglycosides | 0/1 (0) | 0/0 (0) | p>0.999 |
| KL140 | Penicillins | 1/1 (100) | 0/0 (0) | p>0.999 |
| KL140 | Cephalosporins | 0/1 (0) | 0/0 (0) | p>0.999 |
| KL140 | Carbapenems | 0/1 (0) | 0/0 (0) | p>0.999 |
| KL140 | Polymyxin | 0/1 (0) | 0/0 (0) | p>0.999 |
| KL140 | Fluoroquinolones | 0/1 (0) | 0/0 (0) | p>0.999 |
| KL140 | Sulfonamides | 0/1 (0) | 0/0 (0) | p>0.999 |
| KL140 | Tetracyclines | 0/1 (0) | 0/0 (0) | p>0.999 |
| KL140 | ESBL | 0/1 (0) | 0/0 (0) | p>0.999 |
| KL140 | MDR | 0/1 (0) | 0/0 (0) | p>0.999 |
| KL142 | Aminoglycosides | 0/1 (0) | 0/0 (0) | p>0.999 |
| KL142 | Penicillins | 1/1 (100) | 0/0 (0) | p>0.999 |
| KL142 | Cephalosporins | 0/1 (0) | 0/0 (0) | p>0.999 |
| KL142 | Carbapenems | 0/1 (0) | 0/0 (0) | p>0.999 |
| KL142 | Polymyxin | 0/1 (0) | 0/0 (0) | p>0.999 |
| KL142 | Fluoroquinolones | 0/1 (0) | 0/0 (0) | p>0.999 |
| KL142 | Sulfonamides | 0/1 (0) | 0/0 (0) | p>0.999 |
| KL142 | Tetracyclines | 0/1 (0) | 0/0 (0) | p>0.999 |
| KL142 | ESBL | 0/1 (0) | 0/0 (0) | p>0.999 |
| KL142 | MDR | 0/1 (0) | 0/0 (0) | p>0.999 |
| KL149 | Aminoglycosides | 0/0 (0) | 27/28 (96.429) | p>0.999 |
| KL149 | Penicillins | 0/0 (0) | 28/28 (100) | p>0.999 |
| KL149 | Cephalosporins | 0/0 (0) | 27/28 (96.429) | p>0.999 |
| KL149 | Carbapenems | 0/0 (0) | 6/28 (21.429) | p>0.999 |
| KL149 | Polymyxin | 0/0 (0) | 0/28 (0) | p>0.999 |
| KL149 | Fluoroquinolones | 0/0 (0) | 8/28 (28.571) | p>0.999 |
| KL149 | Sulfonamides | 0/0 (0) | 8/28 (28.571) | p>0.999 |
| KL149 | Tetracyclines | 0/0 (0) | 2/28 (7.143) | p>0.999 |
| KL149 | ESBL | 0/0 (0) | 27/28 (96.429) | p>0.999 |
| KL149 | MDR | 0/0 (0) | 27/28 (96.429) | p>0.999 |
| KL15 | Aminoglycosides | 0/5 (0) | 0/0 (0) | p>0.999 |
| KL15 | Penicillins | 5/5 (100) | 0/0 (0) | p>0.999 |
| KL15 | Cephalosporins | 0/5 (0) | 0/0 (0) | p>0.999 |
| KL15 | Carbapenems | 0/5 (0) | 0/0 (0) | p>0.999 |
| KL15 | Polymyxin | 0/5 (0) | 0/0 (0) | p>0.999 |
| KL15 | Fluoroquinolones | 0/5 (0) | 0/0 (0) | p>0.999 |
| KL15 | Sulfonamides | 0/5 (0) | 0/0 (0) | p>0.999 |
| KL15 | Tetracyclines | 0/5 (0) | 0/0 (0) | p>0.999 |
| KL15 | ESBL | 0/5 (0) | 0/0 (0) | p>0.999 |
| KL15 | MDR | 0/5 (0) | 0/0 (0) | p>0.999 |
| KL158 | Aminoglycosides | 0/1 (0) | 0/0 (0) | p>0.999 |
| KL158 | Penicillins | 1/1 (100) | 0/0 (0) | p>0.999 |
| KL158 | Cephalosporins | 0/1 (0) | 0/0 (0) | p>0.999 |
| KL158 | Carbapenems | 0/1 (0) | 0/0 (0) | p>0.999 |
| KL158 | Polymyxin | 0/1 (0) | 0/0 (0) | p>0.999 |
| KL158 | Fluoroquinolones | 0/1 (0) | 0/0 (0) | p>0.999 |
| KL158 | Sulfonamides | 0/1 (0) | 0/0 (0) | p>0.999 |
| KL158 | Tetracyclines | 0/1 (0) | 0/0 (0) | p>0.999 |
| KL158 | ESBL | 0/1 (0) | 0/0 (0) | p>0.999 |
| KL158 | MDR | 0/1 (0) | 0/0 (0) | p>0.999 |
| KL16 | Aminoglycosides | 0/0 (0) | 0/1 (0) | p>0.999 |
| KL16 | Penicillins | 0/0 (0) | 1/1 (100) | p>0.999 |
| KL16 | Cephalosporins | 0/0 (0) | 1/1 (100) | p>0.999 |
| KL16 | Carbapenems | 0/0 (0) | 0/1 (0) | p>0.999 |
| KL16 | Polymyxin | 0/0 (0) | 0/1 (0) | p>0.999 |
| KL16 | Fluoroquinolones | 0/0 (0) | 0/1 (0) | p>0.999 |
| KL16 | Sulfonamides | 0/0 (0) | 1/1 (100) | p>0.999 |
| KL16 | Tetracyclines | 0/0 (0) | 0/1 (0) | p>0.999 |
| KL16 | ESBL | 0/0 (0) | 1/1 (100) | p>0.999 |
| KL16 | MDR | 0/0 (0) | 1/1 (100) | p>0.999 |
| KL17 | Aminoglycosides | 21/22 (95.455) | 0/0 (0) | p>0.999 |
| KL17 | Penicillins | 22/22 (100) | 0/0 (0) | p>0.999 |
| KL17 | Cephalosporins | 20/22 (90.909) | 0/0 (0) | p>0.999 |
| KL17 | Carbapenems | 1/22 (4.545) | 0/0 (0) | p>0.999 |
| KL17 | Polymyxin | 1/22 (4.545) | 0/0 (0) | p>0.999 |
| KL17 | Fluoroquinolones | 20/22 (90.909) | 0/0 (0) | p>0.999 |
| KL17 | Sulfonamides | 21/22 (95.455) | 0/0 (0) | p>0.999 |
| KL17 | Tetracyclines | 22/22 (100) | 0/0 (0) | p>0.999 |
| KL17 | ESBL | 20/22 (90.909) | 0/0 (0) | p>0.999 |
| KL17 | MDR | 21/22 (95.455) | 0/0 (0) | p>0.999 |
| KL2 | Aminoglycosides | 0/0 (0) | 8/9 (88.889) | p>0.999 |
| KL2 | Penicillins | 0/0 (0) | 8/9 (88.889) | p>0.999 |
| KL2 | Cephalosporins | 0/0 (0) | 7/9 (77.778) | p>0.999 |
| KL2 | Carbapenems | 0/0 (0) | 1/9 (11.111) | p>0.999 |
| KL2 | Polymyxin | 0/0 (0) | 1/9 (11.111) | p>0.999 |
| KL2 | Fluoroquinolones | 0/0 (0) | 0/9 (0) | p>0.999 |
| KL2 | Sulfonamides | 0/0 (0) | 8/9 (88.889) | p>0.999 |
| KL2 | Tetracyclines | 0/0 (0) | 0/9 (0) | p>0.999 |
| KL2 | ESBL | 0/0 (0) | 7/9 (77.778) | p>0.999 |
| KL2 | MDR | 0/0 (0) | 8/9 (88.889) | p>0.999 |
| KL24 | Aminoglycosides | 0/4 (0) | 0/0 (0) | p>0.999 |
| KL24 | Penicillins | 4/4 (100) | 0/0 (0) | p>0.999 |
| KL24 | Cephalosporins | 0/4 (0) | 0/0 (0) | p>0.999 |
| KL24 | Carbapenems | 0/4 (0) | 0/0 (0) | p>0.999 |
| KL24 | Polymyxin | 0/4 (0) | 0/0 (0) | p>0.999 |
| KL24 | Fluoroquinolones | 0/4 (0) | 0/0 (0) | p>0.999 |
| KL24 | Sulfonamides | 3/4 (75) | 0/0 (0) | p>0.999 |
| KL24 | Tetracyclines | 0/4 (0) | 0/0 (0) | p>0.999 |
| KL24 | ESBL | 0/4 (0) | 0/0 (0) | p>0.999 |
| KL24 | MDR | 0/4 (0) | 0/0 (0) | p>0.999 |
| KL25 | Aminoglycosides | 18/20 (90) | 21/21 (100) | p=0.232 |
| KL25 | Penicillins | 20/20 (100) | 21/21 (100) | p>0.999 |
| KL25 | Cephalosporins | 14/20 (70) | 19/21 (90.476) | p=0.13 |
| KL25 | Carbapenems | 17/20 (85) | 6/21 (28.571) | p<0.001 |
| KL25 | Polymyxin | 0/20 (0) | 0/21 (0) | p>0.999 |
| KL25 | Fluoroquinolones | 15/20 (75) | 5/21 (23.81) | p=0.002 |
| KL25 | Sulfonamides | 15/20 (75) | 21/21 (100) | p=0.021 |
| KL25 | Tetracyclines | 1/20 (5) | 1/21 (4.762) | p>0.999 |
| KL25 | ESBL | 14/20 (70) | 19/21 (90.476) | p=0.13 |
| KL25 | MDR | 18/20 (90) | 21/21 (100) | p=0.232 |
| KL28 | Aminoglycosides | 0/0 (0) | 0/2 (0) | p>0.999 |
| KL28 | Penicillins | 0/0 (0) | 2/2 (100) | p>0.999 |
| KL28 | Cephalosporins | 0/0 (0) | 0/2 (0) | p>0.999 |
| KL28 | Carbapenems | 0/0 (0) | 0/2 (0) | p>0.999 |
| KL28 | Polymyxin | 0/0 (0) | 0/2 (0) | p>0.999 |
| KL28 | Fluoroquinolones | 0/0 (0) | 0/2 (0) | p>0.999 |
| KL28 | Sulfonamides | 0/0 (0) | 0/2 (0) | p>0.999 |
| KL28 | Tetracyclines | 0/0 (0) | 0/2 (0) | p>0.999 |
| KL28 | ESBL | 0/0 (0) | 0/2 (0) | p>0.999 |
| KL28 | MDR | 0/0 (0) | 0/2 (0) | p>0.999 |
| KL3 | Aminoglycosides | 2/2 (100) | 3/3 (100) | p>0.999 |
| KL3 | Penicillins | 2/2 (100) | 3/3 (100) | p>0.999 |
| KL3 | Cephalosporins | 0/2 (0) | 3/3 (100) | p=0.1 |
| KL3 | Carbapenems | 0/2 (0) | 0/3 (0) | p>0.999 |
| KL3 | Polymyxin | 0/2 (0) | 0/3 (0) | p>0.999 |
| KL3 | Fluoroquinolones | 0/2 (0) | 1/3 (33.333) | p>0.999 |
| KL3 | Sulfonamides | 2/2 (100) | 3/3 (100) | p>0.999 |
| KL3 | Tetracyclines | 2/2 (100) | 1/3 (33.333) | p=0.4 |
| KL3 | ESBL | 0/2 (0) | 3/3 (100) | p=0.1 |
| KL3 | MDR | 2/2 (100) | 3/3 (100) | p>0.999 |
| KL30 | Aminoglycosides | 0/1 (0) | 2/2 (100) | p=0.333 |
| KL30 | Penicillins | 1/1 (100) | 2/2 (100) | p>0.999 |
| KL30 | Cephalosporins | 0/1 (0) | 2/2 (100) | p=0.333 |
| KL30 | Carbapenems | 0/1 (0) | 1/2 (50) | p>0.999 |
| KL30 | Polymyxin | 0/1 (0) | 0/2 (0) | p>0.999 |
| KL30 | Fluoroquinolones | 0/1 (0) | 2/2 (100) | p=0.333 |
| KL30 | Sulfonamides | 0/1 (0) | 2/2 (100) | p=0.333 |
| KL30 | Tetracyclines | 0/1 (0) | 1/2 (50) | p>0.999 |
| KL30 | ESBL | 0/1 (0) | 2/2 (100) | p=0.333 |
| KL30 | MDR | 0/1 (0) | 2/2 (100) | p=0.333 |
| KL38 | Aminoglycosides | 0/1 (0) | 0/0 (0) | p>0.999 |
| KL38 | Penicillins | 1/1 (100) | 0/0 (0) | p>0.999 |
| KL38 | Cephalosporins | 0/1 (0) | 0/0 (0) | p>0.999 |
| KL38 | Carbapenems | 0/1 (0) | 0/0 (0) | p>0.999 |
| KL38 | Polymyxin | 0/1 (0) | 0/0 (0) | p>0.999 |
| KL38 | Fluoroquinolones | 0/1 (0) | 0/0 (0) | p>0.999 |
| KL38 | Sulfonamides | 0/1 (0) | 0/0 (0) | p>0.999 |
| KL38 | Tetracyclines | 0/1 (0) | 0/0 (0) | p>0.999 |
| KL38 | ESBL | 0/1 (0) | 0/0 (0) | p>0.999 |
| KL38 | MDR | 0/1 (0) | 0/0 (0) | p>0.999 |
| KL39 | Aminoglycosides | 0/1 (0) | 0/0 (0) | p>0.999 |
| KL39 | Penicillins | 1/1 (100) | 0/0 (0) | p>0.999 |
| KL39 | Cephalosporins | 0/1 (0) | 0/0 (0) | p>0.999 |
| KL39 | Carbapenems | 0/1 (0) | 0/0 (0) | p>0.999 |
| KL39 | Polymyxin | 1/1 (100) | 0/0 (0) | p>0.999 |
| KL39 | Fluoroquinolones | 0/1 (0) | 0/0 (0) | p>0.999 |
| KL39 | Sulfonamides | 0/1 (0) | 0/0 (0) | p>0.999 |
| KL39 | Tetracyclines | 0/1 (0) | 0/0 (0) | p>0.999 |
| KL39 | ESBL | 0/1 (0) | 0/0 (0) | p>0.999 |
| KL39 | MDR | 0/1 (0) | 0/0 (0) | p>0.999 |
| KL42 | Aminoglycosides | 0/1 (0) | 0/0 (0) | p>0.999 |
| KL42 | Penicillins | 1/1 (100) | 0/0 (0) | p>0.999 |
| KL42 | Cephalosporins | 0/1 (0) | 0/0 (0) | p>0.999 |
| KL42 | Carbapenems | 0/1 (0) | 0/0 (0) | p>0.999 |
| KL42 | Polymyxin | 0/1 (0) | 0/0 (0) | p>0.999 |
| KL42 | Fluoroquinolones | 0/1 (0) | 0/0 (0) | p>0.999 |
| KL42 | Sulfonamides | 0/1 (0) | 0/0 (0) | p>0.999 |
| KL42 | Tetracyclines | 0/1 (0) | 0/0 (0) | p>0.999 |
| KL42 | ESBL | 0/1 (0) | 0/0 (0) | p>0.999 |
| KL42 | MDR | 0/1 (0) | 0/0 (0) | p>0.999 |
| KL45 | Aminoglycosides | 0/1 (0) | 0/0 (0) | p>0.999 |
| KL45 | Penicillins | 1/1 (100) | 0/0 (0) | p>0.999 |
| KL45 | Cephalosporins | 0/1 (0) | 0/0 (0) | p>0.999 |
| KL45 | Carbapenems | 0/1 (0) | 0/0 (0) | p>0.999 |
| KL45 | Polymyxin | 0/1 (0) | 0/0 (0) | p>0.999 |
| KL45 | Fluoroquinolones | 0/1 (0) | 0/0 (0) | p>0.999 |
| KL45 | Sulfonamides | 0/1 (0) | 0/0 (0) | p>0.999 |
| KL45 | Tetracyclines | 0/1 (0) | 0/0 (0) | p>0.999 |
| KL45 | ESBL | 0/1 (0) | 0/0 (0) | p>0.999 |
| KL45 | MDR | 0/1 (0) | 0/0 (0) | p>0.999 |
| KL46 | Aminoglycosides | 0/0 (0) | 0/1 (0) | p>0.999 |
| KL46 | Penicillins | 0/0 (0) | 1/1 (100) | p>0.999 |
| KL46 | Cephalosporins | 0/0 (0) | 0/1 (0) | p>0.999 |
| KL46 | Carbapenems | 0/0 (0) | 0/1 (0) | p>0.999 |
| KL46 | Polymyxin | 0/0 (0) | 0/1 (0) | p>0.999 |
| KL46 | Fluoroquinolones | 0/0 (0) | 0/1 (0) | p>0.999 |
| KL46 | Sulfonamides | 0/0 (0) | 0/1 (0) | p>0.999 |
| KL46 | Tetracyclines | 0/0 (0) | 0/1 (0) | p>0.999 |
| KL46 | ESBL | 0/0 (0) | 0/1 (0) | p>0.999 |
| KL46 | MDR | 0/0 (0) | 0/1 (0) | p>0.999 |
| KL51 | Aminoglycosides | 1/1 (100) | 1/1 (100) | p>0.999 |
| KL51 | Penicillins | 1/1 (100) | 1/1 (100) | p>0.999 |
| KL51 | Cephalosporins | 0/1 (0) | 1/1 (100) | p>0.999 |
| KL51 | Carbapenems | 1/1 (100) | 0/1 (0) | p>0.999 |
| KL51 | Polymyxin | 0/1 (0) | 0/1 (0) | p>0.999 |
| KL51 | Fluoroquinolones | 1/1 (100) | 0/1 (0) | p>0.999 |
| KL51 | Sulfonamides | 1/1 (100) | 1/1 (100) | p>0.999 |
| KL51 | Tetracyclines | 0/1 (0) | 1/1 (100) | p>0.999 |
| KL51 | ESBL | 0/1 (0) | 1/1 (100) | p>0.999 |
| KL51 | MDR | 1/1 (100) | 1/1 (100) | p>0.999 |
| KL60 | Aminoglycosides | 0/1 (0) | 0/0 (0) | p>0.999 |
| KL60 | Penicillins | 1/1 (100) | 0/0 (0) | p>0.999 |
| KL60 | Cephalosporins | 0/1 (0) | 0/0 (0) | p>0.999 |
| KL60 | Carbapenems | 0/1 (0) | 0/0 (0) | p>0.999 |
| KL60 | Polymyxin | 0/1 (0) | 0/0 (0) | p>0.999 |
| KL60 | Fluoroquinolones | 0/1 (0) | 0/0 (0) | p>0.999 |
| KL60 | Sulfonamides | 0/1 (0) | 0/0 (0) | p>0.999 |
| KL60 | Tetracyclines | 0/1 (0) | 0/0 (0) | p>0.999 |
| KL60 | ESBL | 0/1 (0) | 0/0 (0) | p>0.999 |
| KL60 | MDR | 0/1 (0) | 0/0 (0) | p>0.999 |
| KL61 | Aminoglycosides | 0/1 (0) | 0/0 (0) | p>0.999 |
| KL61 | Penicillins | 1/1 (100) | 0/0 (0) | p>0.999 |
| KL61 | Cephalosporins | 0/1 (0) | 0/0 (0) | p>0.999 |
| KL61 | Carbapenems | 0/1 (0) | 0/0 (0) | p>0.999 |
| KL61 | Polymyxin | 0/1 (0) | 0/0 (0) | p>0.999 |
| KL61 | Fluoroquinolones | 0/1 (0) | 0/0 (0) | p>0.999 |
| KL61 | Sulfonamides | 0/1 (0) | 0/0 (0) | p>0.999 |
| KL61 | Tetracyclines | 0/1 (0) | 0/0 (0) | p>0.999 |
| KL61 | ESBL | 0/1 (0) | 0/0 (0) | p>0.999 |
| KL61 | MDR | 0/1 (0) | 0/0 (0) | p>0.999 |
| KL62 | Aminoglycosides | 0/1 (0) | 3/3 (100) | p=0.25 |
| KL62 | Penicillins | 1/1 (100) | 3/3 (100) | p>0.999 |
| KL62 | Cephalosporins | 0/1 (0) | 3/3 (100) | p=0.25 |
| KL62 | Carbapenems | 0/1 (0) | 0/3 (0) | p>0.999 |
| KL62 | Polymyxin | 0/1 (0) | 0/3 (0) | p>0.999 |
| KL62 | Fluoroquinolones | 0/1 (0) | 0/3 (0) | p>0.999 |
| KL62 | Sulfonamides | 0/1 (0) | 0/3 (0) | p>0.999 |
| KL62 | Tetracyclines | 0/1 (0) | 0/3 (0) | p>0.999 |
| KL62 | ESBL | 0/1 (0) | 3/3 (100) | p=0.25 |
| KL62 | MDR | 0/1 (0) | 3/3 (100) | p=0.25 |
| KL63 | Aminoglycosides | 0/1 (0) | 0/0 (0) | p>0.999 |
| KL63 | Penicillins | 1/1 (100) | 0/0 (0) | p>0.999 |
| KL63 | Cephalosporins | 0/1 (0) | 0/0 (0) | p>0.999 |
| KL63 | Carbapenems | 0/1 (0) | 0/0 (0) | p>0.999 |
| KL63 | Polymyxin | 0/1 (0) | 0/0 (0) | p>0.999 |
| KL63 | Fluoroquinolones | 0/1 (0) | 0/0 (0) | p>0.999 |
| KL63 | Sulfonamides | 0/1 (0) | 0/0 (0) | p>0.999 |
| KL63 | Tetracyclines | 0/1 (0) | 0/0 (0) | p>0.999 |
| KL63 | ESBL | 0/1 (0) | 0/0 (0) | p>0.999 |
| KL63 | MDR | 0/1 (0) | 0/0 (0) | p>0.999 |
| KL7 | Aminoglycosides | 0/0 (0) | 0/1 (0) | p>0.999 |
| KL7 | Penicillins | 0/0 (0) | 1/1 (100) | p>0.999 |
| KL7 | Cephalosporins | 0/0 (0) | 0/1 (0) | p>0.999 |
| KL7 | Carbapenems | 0/0 (0) | 0/1 (0) | p>0.999 |
| KL7 | Polymyxin | 0/0 (0) | 0/1 (0) | p>0.999 |
| KL7 | Fluoroquinolones | 0/0 (0) | 0/1 (0) | p>0.999 |
| KL7 | Sulfonamides | 0/0 (0) | 0/1 (0) | p>0.999 |
| KL7 | Tetracyclines | 0/0 (0) | 0/1 (0) | p>0.999 |
| KL7 | ESBL | 0/0 (0) | 0/1 (0) | p>0.999 |
| KL7 | MDR | 0/0 (0) | 0/1 (0) | p>0.999 |
| KL8 | Aminoglycosides | 7/7 (100) | 1/1 (100) | p>0.999 |
| KL8 | Penicillins | 7/7 (100) | 1/1 (100) | p>0.999 |
| KL8 | Cephalosporins | 7/7 (100) | 1/1 (100) | p>0.999 |
| KL8 | Carbapenems | 0/7 (0) | 0/1 (0) | p>0.999 |
| KL8 | Polymyxin | 0/7 (0) | 0/1 (0) | p>0.999 |
| KL8 | Fluoroquinolones | 6/7 (85.714) | 1/1 (100) | p>0.999 |
| KL8 | Sulfonamides | 7/7 (100) | 1/1 (100) | p>0.999 |
| KL8 | Tetracyclines | 0/7 (0) | 0/1 (0) | p>0.999 |
| KL8 | ESBL | 7/7 (100) | 1/1 (100) | p>0.999 |
| KL8 | MDR | 7/7 (100) | 1/1 (100) | p>0.999 |
| KL9 | Aminoglycosides | 1/2 (50) | 0/0 (0) | p>0.999 |
| KL9 | Penicillins | 2/2 (100) | 0/0 (0) | p>0.999 |
| KL9 | Cephalosporins | 0/2 (0) | 0/0 (0) | p>0.999 |
| KL9 | Carbapenems | 0/2 (0) | 0/0 (0) | p>0.999 |
| KL9 | Polymyxin | 0/2 (0) | 0/0 (0) | p>0.999 |
| KL9 | Fluoroquinolones | 1/2 (50) | 0/0 (0) | p>0.999 |
| KL9 | Sulfonamides | 1/2 (50) | 0/0 (0) | p>0.999 |
| KL9 | Tetracyclines | 0/2 (0) | 0/0 (0) | p>0.999 |
| KL9 | ESBL | 0/2 (0) | 0/0 (0) | p>0.999 |
| KL9 | MDR | 1/2 (50) | 0/0 (0) | p>0.999 |

Abbreviations: ESBL extended spectrum β-lactamase, MDR – Multi-drug resistance, ST = sequence type.

P-values of 0.05 are considered significant
